# Design, synthesis, and evaluation of novel coumarin-dithiocarbamate derivatives (IDs) as anti-colorectal cancer agents

**DOI:** 10.1080/14756366.2021.1875458

**Published:** 2021-02-08

**Authors:** Heping Zhu, Shilong Ying, Bingluo Zhou, Xinyang Hu, Xiao Liang, Wangyu Li, Dungai Wang, Hongchuan Jin, Yuanjiang Pan

**Affiliations:** a Department of Chemistry, Zhejiang University, Hangzhou, P. R. China; b Laboratory of Cancer Biology, Key Lab of Biotherapy in Zhejiang, Sir Run Run Shaw Hospital, Medical School of Zhejiang University, Hangzhou, P. R. China

**Keywords:** Colorectal cancer, coumarin-dithiocarbamate derivatives, bromodomain-containing protein 4, antitumor activities

## Abstract

Colorectal cancer (CRC) is a common malignant tumour of human digestive tract. The high mortality rate of CRC is closely related to the limitations of existing treatments. Thus, there is an urgent need to search for new anti-CRC agents. In this work, twenty novel coumarin-dithiocarbamate derivatives (**IDs**) were designed, synthesized and evaluated *in vitro*. The results suggest that the most active compound **ID-11** effectively inhibited the proliferation of CRC cell lines while shown little impact on normal colon epithelial cells. Mechanism studies revealed that **ID-11** displayed bromodomain-containing protein 4 inhibitory activity, and induced G2/M phase arrest, apoptosis as well as decreased the expression levels of the key genes such as *c-Myc* and *Bcl-2* in CRC cell lines. Moreover, the ADMET properties prediction results shown that **ID-11** possess well metabolic characteristics without obvious toxicities. Our data demonstrated that compound **ID-11** may be a promising anti-CRC agent and deserved for further development.

## 1. Introduction

Colorectal cancer (CRC) is the third most commonly diagnosed cancer worldwide. In 2018, nearly 1.8 million new cases occurred and 881 000 patients died^1^. It was predicted that the number of deaths for both colon and rectal cancer would increase by 60.0 and 71.5% in all countries until 2035, respectively^2^. Like other types of cancer, CRC is the result of accumulation of genetic and epigenetic mutations in specific oncogenes and/or tumour suppressor genes. Besides familial CRC (about 10–30%), CRC often occurred in patients with a defined hereditary syndrome (about 5%), sporadic adenomatous colorectal polyps (about 75%) and extensive ulcerative colitis (about 4%)^3^. Currently, surgical section combined with radiotherapy and chemotherapy still be the main treatments for CRC. However, drug resistance and the severe side effects of these therapies are inevitable, the quality of life of patients would be severely affected. Although some new treatments such as immunotherapy have emerged in recent years, the high costs of which would be a burden for most patients^4–7^. Therefore, exploring for new anti-CRC agents is of great significance.

The bromodomain-containing protein 4 (BRD4) is one of the most important member of BET (bromodomain and extra-terminal domain) family of transcriptional regulatory proteins, which specially recognise acetylated lysine residues on histones with its bromodomains and recruits transcriptional regulatory complexes to acetylated chromatin.^8^ Previous studies have shown that the expression disorder of BRD4 is associated with the progression of many cancers including CRC^8^^,^^9^. As BRD4 affects the transcription process of some key oncogenes such as *c-Myc*, inhibiting the activity of BRD4 may produce potential anticancer effect^10^. Compounds such as **(+)-JQ1**^11^, which specifically designed as a BRD4 inhibitor, had been proved to own powerful anticancer activities. Thus, targeting BRD4 represents a promising therapeutic strategy against CRC.

**Figure 1.**
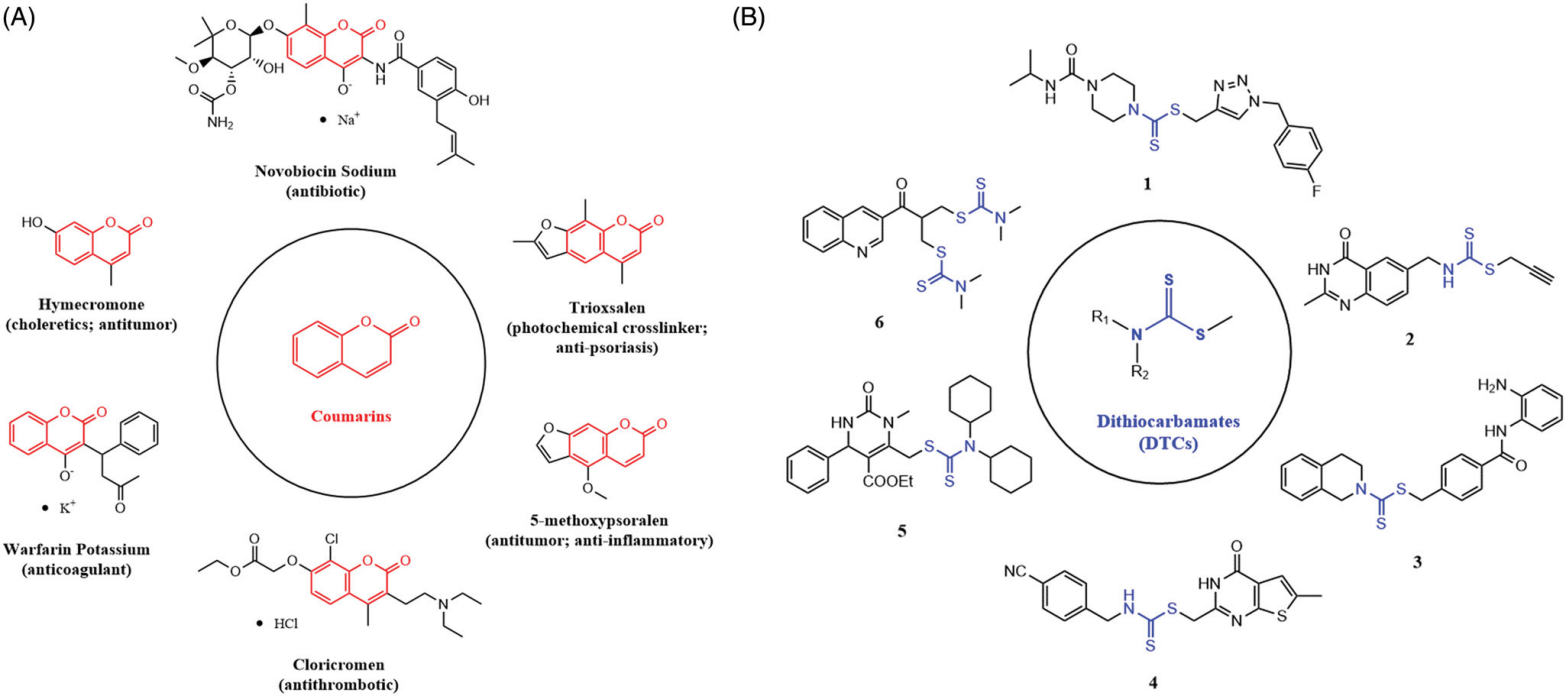
Drugs containing coumarin core (A) and antitumor agents containing dithiocarbamate fragment (B).

Over the past few decades, natural products play an important role in the discovery of anticancer drugs^12–14^. Coumarin (benzopyran-2-one, Figure 1) is a classic natural product original from plants, bacteria and fungi^15^^,^^16^. The scaffold of coumarin had been widely used in drug design, especially in anticancer drugs^17–19^ (Figure 1). Actually, the targets of different anticancer coumarins vary much from each other, mainly involved carbonic anhydrase (isoforms IX and XII)^20–23^, STAT3^24^, tubulin^25^, topoisomerase II^26^, and BRD4^27^, etc. Dithiocarbamates (DTCs) is another important pharmacophore, which often be incorporated into the structure of antitumor agents, such as compound **1**^28^ (against MCF-7, MGC-823, SMMC-7721 and EC-9706 cell lines), compound **2**^29^ (against A549, MCF-7, Hela, HT-29 and HCT-116 cell lines), compound **3**^30^ (against A375, MGC-803, Hela, SMMC-7721, H1299 and HCT-116 cell lines), compound **4**^31^ (against A549 and HCT-116 cell lines), compound **5**^32^ (against leukaemia, non-small cell lung cancer, melanoma, etc.) and compound **6**^33^ (against H460, HepG2, MCF-7 cell lines, etc.) (Figure 1). Likewise, anticancer agents containing DTCs fragment also have different targets, including carbonic anhydrase (isoforms IX and XII)^34^^,^^35^, VEGFR-2^36^ and FAK^37^, etc.

**Figure 2.**
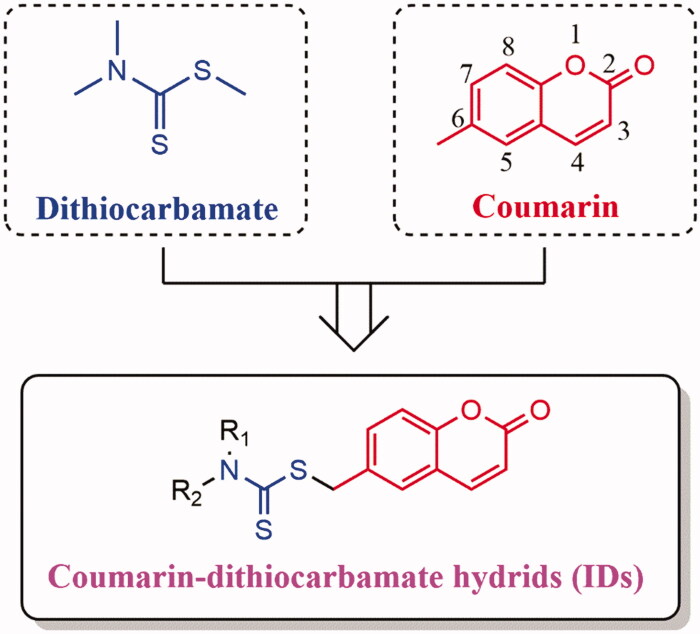
Design strategy of the novel coumarin-dithiocarbamate derivatives (**IDs**).

To our knowledge, there are few reports about the hybrids between coumarins and DTCs as potential anti-CRC agents. In this study, we focus on the modification of position C-6 of coumarin scaffold and hybridized with the DTCs. A series of novel coumarin-dithiocarbamate derivatives (Figure 2), which we named **IDs** uniformly, were designed and synthesized. Herein, we reported the discovery of these **IDs** as potential anti-CRC agents and explored their underlying anticancer mechanisms including BRD4 inhibition assay, the effects on cell cycle, cell apoptosis as well as the expression levels of *c-Myc* and *Bcl-2* in CRC cell lines *in vitro*. In addition, the physicochemical properties of the most active agent were also investigated.

## 2. Materials and methods

### 2.1. Chemistry

All chemicals were purchased from Aladdin (Shanghai, China) and Energy Chemical (Shanghai, China) and used without further purification. Reaction progress was monitored by thin layer chromatography (TLC) on precoated silica gel GF254 plates (Qingdao Haiyang Chemical Plant, Qingdao, China) and visualised under UV light (254 nm). ^1^H NMR and ^13 ^C NMR spectra were recorded on Bruker AVANCE III 400 or 600 spectrometer with DMSO-d_6_ or CDCl_3_ as the solvent, and the chemical shifts (d) given in parts per million (ppm) relative to tetramethylsilane (TMS) as an internal standard. Coupling constants (*J*) were reported in hertz, and splitting patterns were used as follows: s, singlet; br. s, broad singlet; d, doublet; dd, doublet of doublets; t, triplet; m, multiplet. High resolution mass spectrometry (HRMS) spectra were recorded on SHIMADZU LCMS-IT-TOF with an electrospray ion source (ESI). Melting point was measured on an SGW®X-4A micro melting point apparatus (Shanghai INESA Physico-Optical Instrument Co., Ltd, China).

### 2.2. General procedures

#### 2.2.1. Synthesis of intermediate 6-(bromomethyl)-2*H*-chromen-2-one (9)

6-Methyl-2*H*-chromen-2-one (**7**, 1.00 g, 6.24 mmol) and 2,2′-dimethyl-2,2′-azodipropionitrile (102 mg, 0.624 mmol) was dissolved in CCl_4_ (25 ml), and then the 1-bromopyrrolidine-2,5-dione (**8**, 1.11 g, 6.24 mmol) was added in batches at room temperature within 15 min. After that, the mixture was transferred, stirred and refluxed at 90 °C for another 10 h until the reaction finished. Then the CCl_4_ was removed under reduced pressure, and the residue containing 6-(bromomethyl)-2*H*-chromen-2-one (**9)** was used in next step directly without further purification.

#### 2.2.2. General procedures for the preparation of final products ID-1 to ID-20

First, dimethylamine (28 μL, 0.557 mmol) and KOH (31 mg, 0.557 mmol) was dissolved in dry ethanol (10 ml), and the mixture was stirred at room temperature for 20 min. Then, CS_2_ (33 μL, 0.557 mmol) was added and stirred for another 30 min. After that, intermediate **9** (100 mg, 0.418 mmol) was added slowly and continue to stir at room temperature for another 24 h. After the reaction finished, ethanol was removed under reduced pressure, and the residue was extracted with ethyl acetate/H_2_O (*v/v*, 100 ml) for three times. The ethyl acetate layer was collected, concentrated, and purified by silica gel column chromatography (PE: EA = 5: 1) to afford the title product **ID-1**.

Compounds **ID-6**, **ID-7**, **ID10**, **ID-12**, **ID-15,** and **ID-20** were prepared with a similar procedure which described for the synthesis of compound **ID-1**. For the synthesis of compounds **ID-2** ∼ **ID-5**, **ID-8**, **ID-9**, **ID-13**, **ID-14,** and **ID-16** ∼ **ID-19**, in which the solvent was dioxane/H_2_O instead of dry ethanol and the alkali was K_2_CO_3_ instead of KOH. For the synthesis of compound **ID-11**, actone was used as the solvent and K_3_PO_4_ was used as the alkali.

##### 2.2.2.1. (2-Oxo-2*H*-chromen-6-yl)methyl dimethylcarbamodithioate (**ID-1**)

White powder. Yield: 71%. m.p: 151.6–152.5 °C. ^1^H NMR (400 MHz, CDCl_3_) δ (ppm): 7.67 (1H, d, *J* = 9.2 Hz, H-4), 7.54 (1H, d, *J* =7.2 Hz, H-7), 7.53 (1H, s, H-5), 7.26 (1H, d, *J* = 6.4 Hz, H-8), 6.41 (1H, d, *J* = 9.2 Hz, H-3), 4.61 (2H, s, –SCH_2_–), 3.55 (3H, s, –N(CH_3_)_2_), 3.36 (3H, s, –N(CH_3_)_2_). ^13 ^C NMR (100 MHz, CDCl_3_) δ (ppm): 195.9, 160.7, 153.2, 143.3, 133.3, 132.8, 128.3, 118.7, 117.0, 116.8, 45.6, 41.5, 41.0. HRMS (ESI, *m/z*) calcd. for C_13_H_13_NO_2_S_2_ (M + H)^+^ 280.0460, found 208.0461.

##### 2.2.2.2. (2-Oxo-2*H*-chromen-6-yl)methyl diethylcarbamodithioate (**ID-2**)

White powder. Yield: 50%. m.p: 100.1–101.0 °C. ^1^H NMR (400 MHz, DMSO-d_6_) δ (ppm): 8.04 (1H, d, *J* =9.6 Hz, H-4), 7.73 (1H, d, *J* =2.4 Hz, H-5), 7.62 (1H, dd, *J_1_*=8.8 Hz, *J_2_*=2.4 Hz, H-7), 7.35 (1H, d, *J* =8.4 Hz, H-8), 6.48 (1H, d, *J* = 9.6 Hz, H-3), 4.58 (2H, s, –SCH_2_–), 3.96 (2H, q, *J* = 6.8 Hz, –N(CH_2_CH_3_)_2_), 3.72 (2H, q, *J* =6.8 Hz, –N(CH_2_CH_3_)_2_), 1.21–1.15 (6H, m, –N(CH_2_CH_3_)_2_). ^13 ^C NMR (100 MHz, DMSO-d_6_) δ (ppm): 193.0, 159.8, 152.6, 144.0, 133.0, 132.7, 128.6, 118.5, 116.4 × 2, 49.1, 46.5, 39.6, 12.3, 11.3. HRMS (ESI, *m/z*) calcd. for C_15_H_17_NO_2_S_2_ (M + Na)^+^ 330.0593, found 330.0599.

##### 2.2.2.3. (2-Oxo-2*H*-chromen-6-yl)methyl dipropylcarbamodithioate (**ID-3**)

Yellowish powder. Yield: 60%. m.p: 52.3–53.2 °C. ^1^H NMR (400 MHz, CDCl_3_) δ (ppm): 7.65 (1H, d, *J* = 9.6 Hz, H-4), 7.51–7.48 (2H, m, H-5, H-7), 7.20 (1H, d, *J* = 8.4 Hz, H-8), 6.35 (1H, d, *J* = 9.6 Hz, H-3), 4.55 (2H, s, –SCH_2_–), 3.86 (2H, t, –N(CH_2_CH_2_CH_3_)_2_), 3.56 (2H, t, –N(CH_2_CH_2_CH_3_)_2_), 1.76–1.63 (4H, m, –N(CH_2_CH_2_CH_3_)_2_), 0.88 (6H, t, –N(CH_2_CH_2_CH_3_)_2_). ^13 ^C NMR (100 MHz, CDCl_3_) δ (ppm): 194.8, 160.6, 153.1, 143.3, 133.3, 132.8, 128.4, 118.6, 116.9, 116.7, 57.0, 54.4, 40.7, 20.7, 19.6, 11.1 × 2. HRMS (ESI, *m/z*) calcd. for C_17_H_21_NO_2_S_2_ (M + Na)^+^ 358.0906, found 358.0918.

##### 2.2.2.4. (2-Oxo-2*H*-chromen-6-yl)methyl dibutylcarbamodithioate (**ID-4**)

Yellowish liquid. Yield: 70%. ^1^H NMR (400 MHz, CDCl_3_) δ (ppm): 7.69 (1H, d, *J* =9.6 Hz, H-4), 7.55–7.53 (2H, m, H-5, H-7), 7.24 (1H, d, *J* =8.4 Hz, H-8), 6.40 (1H, d, *J* =9.6 Hz, H-3), 4.60 (2H, s, –SCH_2_–), 3.95 (2H, t, *J* = 8.0 Hz, –N(CH_2_CH_2_CH_2_CH_3_)_2_), 3.64 (2H, t, *J* = 8.0 Hz, –N(CH_2_CH_2_CH_2_CH_3_)_2_), 1.74–1.67 (4H, m, –N(CH_2_CH_2_CH_2_CH_3_)_2_), 1.38–1.31 (4H, m, –N(CH_2_CH_2_CH_2_CH_3_)_2_), 0.96–0.91 (6H, m, –N(CH_2_CH_2_CH_2_CH_3_)_2_). ^13 ^C NMR (100 MHz, CDCl_3_) δ (ppm): 194.6, 160.6, 153.2, 143.3, 133.3, 132.9, 128.4, 118.7, 116.9, 116.7, 55.2, 52.5, 40.7, 29.3, 28.3, 20.0 × 2, 13.8, 13.7. HRMS (ESI, *m/z*) calcd. for C_19_H_25_NO_2_S_2_ (M + Na)^+^ 386.1219, found 386.1222.

##### 2.2.2.5. (2-Oxo-2*H*-chromen-6-yl)methyl azetidine-1-carbodithioate (**ID-5**)

White powder. Yield: 56%. m.p: 144.0–145.0 °C. ^1^H NMR (400 MHz, CDCl_3_) δ (ppm): 7.66 (1H, d, *J* =9.6 Hz, H-4), 7.55–7.53 (2H, m, H-5, H-7), 7.25 (1H, d, *J* =9.2 Hz, H-8), 6.40 (1H, d, *J* = 9.6 Hz, H-3), 4.59 (2H, s, –SCH_2_–), 4.30 (2H, t, *J* =7.6 Hz, H-a), 4.17 (2H, t, *J* =7.6 Hz, H-c), 2.43–2.35 (2H, m, H-b). ^13 ^C NMR (100 MHz, CDCl_3_) δ (ppm): 192.8, 160.7, 153.2, 143.2, 133.9, 132.7, 128.2, 118.7, 117.0, 116.8, 54.8, 53.1, 38.9, 15.5. HRMS (ESI, *m/z*) calcd. for C_14_H_13_NO_2_S_2_ (M + Na)^+^ 314.0280, found 314.0281.

##### 2.2.2.6. (2-Oxo-2*H*-chromen-6-yl)methyl 3-hydroxyazetidine-1-carbodithioate (**ID-6**)

White powder. Yield: 55%. m.p: 137.8–138.7 °C. ^1^H NMR (400 MHz, DMSO-d_6_) δ (ppm): 8.04 (1H, d, *J* = 9.6 Hz, H-4), 7.70 (1H, d, *J* =1.2 Hz, H-5), 7.61 (1H, dd, *J_1_* = 8.4 Hz, *J_2_* = 1.6 Hz, H-7), 7.35 (1H, d, *J* = 8.4 Hz, H-8), 6.48 (1H, d, *J* = 9.6 Hz, H-3), 5.94 (1H, d, *J* = 6.4 Hz, –N(CH_2_)_2_CHOH), 4.58–4.52 (3H, m, –SCH_2_–, –N(CH_2_)_2_CHOH), 4.39 (2H, m, –N(CH_2_)_2_CHOH), 3.90–3.84 (2H, m, –N(CH_2_)_2_CHOH). ^13 ^C NMR (100 MHz, DMSO-d_6_) δ (ppm): 191.7, 159.8, 152.6, 144.0, 133.8, 132.6, 128.4, 118.5, 116.4 × 2, 64.4, 62.5, 59.8, 38.2. HRMS (ESI, *m/z*) calcd. for (M + Na)^+^ 330.0229, found 330.0232.

##### 2.2.2.7. (2-Oxo-2*H*-chromen-6-yl)methyl pyrrolidine-1-carbodithioate (**ID-7**)

White powder. Yield: 50%. m.p: 110.2–111.1 °C. ^1^H NMR (400 MHz, CDCl_3_) δ (ppm): 7.67 (1H, d, *J* =9.6 Hz, H-4), 7.56–7.54 (2H, m, H-5, H-7), 7.25 (1H, d, *J* = 9.2 Hz, H-8), 6.40 (1H, d, *J* = 9.6 Hz, H-3), 4.63 (2H, s, –SCH_2_–), 3.93 (2H, t, *J* =6.8 Hz, –NCH_2_CH_2_CH_2_CH_2_N–*(pyrrolidine*)), 3.63 (2H, t, *J* =6.8 Hz, –NCH_2_CH_2_CH_2_CH_2_N–*(pyrrolidine*)), 2.10–2.03 (2H, m, –NCH_2_CH_2_CH_2_CH_2_N–*(pyrrolidine*)), 2.01–1.94 (2H, m, –NCH_2_CH_2_CH_2_CH_2_N–*(pyrrolidine*)). ^13 ^C NMR (100 MHz, CDCl_3_) δ (ppm): 191.5, 160.7, 153.2, 143.3, 133.7, 132.8, 128.3, 118.7, 117.0, 116.8, 55.3, 50.6, 39.8, 26.0, 24.2. HRMS (ESI, *m/z*) calcd. for C_15_H_15_NO_2_S_2_ (M + Na)^+^ 328.0436, found 328.0444.

##### 2.2.2.8. (2-Oxo-2*H*-chromen-6-yl)methyl piperidine-1-carbodithioate (**ID-8**)

Yellowish powder. Yield: 70%. m.p: 130.1–131.0 °C. ^1^H NMR (400 MHz, CDCl_3_) δ (ppm): 7.67 (1H, d, *J* =9.6 Hz, H-4), 7.56–7.53 (2H, m, H-5, H-7), 7.26 (1H, d, *J* =8.0 Hz, H-8), 6.40 (1H, d, *J* = 9.6 Hz, H-3), 4.62 (2H, s, –SCH_2_–), 4.28 (2H, br, H-a), 3.86 (2H, br, H-e), 1.69 (6H, br, H-b, H-c, H-d). ^13 ^C NMR (100 MHz, CDCl_3_) δ (ppm): 194.3, 160.7, 153.2, 143.3, 133.3, 132.9, 128.4, 118.7, 117.0, 116.8, 53.2, 51.4, 40.7, 26.0, 25.4, 24.2. HRMS (ESI, *m/z*) calcd. for C_16_H_17_NO_2_S_2_ (M + Na)^+^ 342.0593, found 342.0585.

##### 2.2.2.9. (2-Oxo-2*H*-chromen-6-yl)methyl azepane-1-carbodithioate (**ID-9**)

White powder. Yield: 66%. m.p: 72.6–73.5 °C. ^1^H NMR (400 MHz, CDCl_3_) δ (ppm): 7.65 (1H, d, *J* = 9.6 Hz, H-4), 7.53–7.50 (2H, m, H-5, H-7), 7.22 (1H, d, *J* = 8.0 Hz, H-8), 6.37 (1H, d, *J* = 9.6 Hz, H-3), 4.58 (2H, s, –SCH_2_–), 4.15 (2H, t, *J* = 6.0 Hz, H-a), 3.83 (2H, t, *J* = 6.0 Hz, H-f), 1.84–1.79 (4H, m, H-b, H-e), 1.23–1.20 (2H, m, H-c), 0.85–0.78 (2H, m, H-d). ^13 ^C NMR (100 MHz, CDCl_3_) δ (ppm): 195.0, 160.6, 153.2, 143.3, 133.4, 132.8, 128.4, 118.7, 116.9, 116.7, 55.8, 52.8, 40.6, 27.3, 26.6, 26.5, 26.1. HRMS (ESI, *m/z*) calcd. for C_17_H_19_NO_2_S_2_ (M + Na)^+^ 356.0749, found 356.0751.

##### 2.2.2.10. (2-Oxo-2*H*-chromen-6-yl)methyl morpholine-4-carbodithioate (**ID-10**)

White powder. Yield: 57%. m.p: 135.4–136.3 °C. ^1^H NMR (600 MHz, DMSO-d_6_) δ (ppm): 8.04 (1H, d, *J* =9.6 Hz, H-4), 7.73 (1H, s, H-5), 7.63 (1H, dd, *J_1_*=8.4 Hz, *J_2_*=1.8 Hz, H-7), 7.35 (1H, d, *J* = 9.0 Hz, H-8), 6.48 (1H, d, *J* =9.0 Hz, H-3), 4.63 (2H, s, –SCH_2_–), 4.22 (2H, br, –N(CH_2_CH_2_)_2_O–*(morpholine)*), 3.90 (2H, br, -N(CH_2_CH_2_)_2_O-(*morpholine)*), 3.65 (4H, br, –N(CH_2_CH_2_)_2_O–(*morpholine)*). ^13 ^C NMR (125 MHz, DMSO-d_6_) δ (ppm): 197.8, 162.9, 155.8, 147.1, 136.0, 135.9, 131.8, 121.7, 119.5, 119.5, 68.6 × 2, 54.4, 53.3, 43.1. HRMS (ESI, *m/z*) calcd. for C_15_H_15_NO_3_S_2_ (M + Na)^+^ 344.0386, found 344.0391.

##### 2.2.2.11. (2-Oxo-2*H*-chromen-6-yl)methyl 1H-imidazole-1-carbodithioate (**ID-11**)

Yellow powder. Yield: 68%. m.p: 139.1–140.0 °C. ^1^H NMR (400 MHz, DMSO-d_6_) δ (ppm): 8.64 (1H, s, H-b), 8.05 (1H, d, *J* =9.6 Hz, H-4), 8.01 (1H, s, H-5), 7.82 (1H, s, H-e), 7.71 (1H, d, *J* = 8.4 Hz, H-7), 7.40 (1H, d, *J* =8.4 Hz, H-8), 7.17 (1H, s, H-d), 6.50 (1H, d, *J* =9.6 Hz, H-3), 4.78 (2H, s, –SCH_2_–). ^13 ^C NMR (100 MHz, DMSO-*d*_6_) δ (ppm): 197.8, 159.7, 153.0, 143.8, 135.9, 133.0, 131.7, 130.6, 129.0, 118.7, 118.3, 116.6 × 2, 39.6. HRMS (ESI, *m/z*) calcd. for C_14_H_10_N_2_O_2_S_2_ (M + Na)^+^ 325.0076, found 325.0070.

##### 2.2.2.12. (2-Oxo-2*H*-chromen-6-yl)methyl 4-methylpiperazine-1-carbodithioate (**ID-12**)

Yellowish powder. Yield: 53%. m.p: 124.3–125.2 °C. ^1^H NMR (400 MHz, DMSO-d_6_) δ (ppm): 8.03 (1H, d, *J* = 9.6 Hz, H-4), 7.73 (1H, d, *J* = 2.0 Hz, H-5), 7.62 (1H, dd, *J_1_*=8.8 Hz, *J_2_*=2.0 Hz, H-7), 7.35 (1H, d, *J* =8.4 Hz, H-8), 6.48 (1H, d, *J* =9.6 Hz, H-3), 4.61 (2H, s, –SCH_2_–), 4.23 (2H, br, –N(CH_2_CH_2_)_2_N–*(piperazine*)), 3.87 (2H, br, –N(CH_2_CH_2_)_2_N–*(piperazine*)), 2.38 (4H, br, –N(CH_2_CH_2_)_2_N–*(piperazine*)), 2.19 (3H, s, –NCH_3_). ^13 ^C NMR (100 MHz, DMSO-d_6_) δ (ppm): 194.1, 159.8, 152.6, 143.9, 132.9, 132.8, 128.7, 118.5, 116.4 × 2, 53.9 × 2, 50.9, 49.5, 45.0 × 2. HRMS (ESI, *m/z*) calcd. for C_16_H_18_N_2_O_2_S_2_ (M + Na)^+^ 357.0702, found 357.0709.

##### 2.2.2.13. (2-Oxo-2*H*-chromen-6-yl)methyl 4-isopropylpiperazine-1-carbodithioate (**ID-13**)

Yellowish powder. Yield: 58%. m.p: 73.6–74.5 °C. ^1^H NMR (400 MHz, DMSO-d_6_) δ (ppm): 8.04 (1H, d, *J* = 9.6 Hz, H-4), 7.73 (1H, d, *J* = 2.0 Hz, H-5), 7.63 (1H, dd, *J_1_*=8.8 Hz, *J_2_*=2.4 Hz, H-7), 7.35 (1H, d, *J* =8.4 Hz, H-8), 6.48 (1H, d, *J* =9.6 Hz, H-3), 4.61 (2H, s, –SCH_2_–), 4.21 (2H, br, –N(CH_2_CH_2_)_2_N–CH(CH_3_)_2_), 3.86 (2H, br, –N(CH_2_CH_2_)_2_N-CH(CH_3_)_2_), 2.71–2.65 (1H, m, –N(CH_2_CH_2_)_2_N–CH(CH_3_)_2_), 2.50–2.49 (4H, m, –N(CH_2_CH_2_)_2_N–CH(CH_3_)_2_), 0.96 (3H, s, –N(CH_2_CH_2_)_2_N–CH(CH_3_)_2_), 0.95 (3H, s, –N(CH_2_CH_2_)_2_N–CH(CH_3_)_2_). ^13 ^C NMR (100 MHz, DMSO-d_6_) δ (ppm): 193.7, 159.8, 152.6, 143.9, 132.9, 132.8, 128.7, 118.5, 116.4 × 2, 53.4, 51.5, 50.1, 47.7, 47.6 × 2, 18.0 × 2. HRMS (ESI, *m/z*) calcd. for C_18_H_22_N_2_O_2_S_2_ (M + H)^+^ 363.1195, found 363.1201.

##### 2.2.2.14. (2-Oxo-2*H*-chromen-6-yl)methyl 4-(tert-butyl)piperazine-1-carbodithioate (**ID-14**)

Yellowish powder. Yield: 62%. m.p: 159.4–160.3 °C. ^1^H NMR (400 MHz, DMSO-d_6_) δ (ppm): 8.04 (1H, d, *J* =9.6 Hz, H-4), 7.73 (1H, d, *J* = 2.0 Hz, H-5), 7.62 (1H, dd, *J_1_*=8.8 Hz, *J_2_*=2.4 Hz, H-7), 7.35 (1H, d, *J* =8.4 Hz, H-8), 6.48 (1H, d, *J* =9.6 Hz, H-3), 4.60 (2H, s, –SCH_2_–), 4.18 (2H, br, –N(CH_2_CH_2_)_2_N–C(CH_3_)_3_), 3.84 (2H, br, –N(CH_2_CH_2_)_2_N–C(CH_3_)_3_), 2.55 (4H, br, –N(CH_2_CH_2_)_2_N–C(CH_3_)_3_), 1.00 (9H, s, –N(CH_2_CH_2_)_2_N–C(CH_3_)_3_). ^13 ^C NMR (100 MHz, DMSO-d_6_) δ (ppm): 193.5, 159.8, 152.6, 143.9, 133.0, 132.8, 128.7, 118.5, 116.4 × 2, 53.3, 51.7, 50.4, 45.4, 45.3, 45.2, 25.5 × 3. HRMS (ESI, *m/z*) calcd. for C_19_H_24_N_2_O_2_S_2_ (M + H)^+^ 377.1352, found 377.1349.

##### 2.2.2.15. (2-Oxo-2*H*-chromen-6-yl)methyl 4–(2-hydroxyethyl)piperazine-1-carbodithioate (**ID-15**)

Yellowish powder. Yield: 43%. m.p: 120.9–121.8 °C. ^1^H NMR (400 MHz, DMSO-d_6_) δ (ppm): 8.04 (1H, d, *J* =9.6 Hz, H-4), 7.73 (1H, d, *J* = 2.0 Hz, H-5), 7.62 (1H, dd, *J_1_*=8.4 Hz, *J_2_*=2.0 Hz, H-7), 7.35 (1H, d, *J* =8.4 Hz, H-8), 6.48 (1H, d, *J* =9.6 Hz, H-3), 4.61 (2H, s, –SCH_2_–), 4.47 (1H, t, *J* =4.8 Hz, –NCH_2_CH_2_OH), 4.22 (2H, br, –N(CH_2_CH_2_)_2_N–*(piperazine*)), 3.87 (2H, br, –N(CH_2_CH_2_)_2_N–*(piperazine*)), 3.50 (2H, q, *J* = 5.6 Hz, –NCH_2_CH_2_OH), 2.50 (4H, br, –N(CH_2_CH_2_)_2_N–*(piperazine*)), 2.42 (2H, t, *J* = 6.0 Hz, –NCH_2_CH_2_OH). ^13 ^C NMR (100 MHz, DMSO-d_6_) δ (ppm): 193.9, 159.8, 152.6, 143.9, 132.9, 132.8, 128.7, 118.5, 116.4 × 2, 59.4, 58.4, 52.5, 52.4, 51.0, 49.6, 39.5. HRMS (ESI, *m/z*) calcd. for C_17_H_20_N_2_O_3_S_2_ (M + Na)^+^ 387.0808, found 387.0808.

##### 2.2.2.16. (2-Oxo-2*H*-chromen-6-yl)methyl 4-cyclohexylpiperazine-1-carbodithioate (**ID-16**)

Yellowish powder. Yield: 68%. m.p: 106.9–107.8 °C. ^1^H NMR (400 MHz, DMSO-d_6_) δ (ppm): 8.04 (1H, d, *J* = 9.6 Hz, H-4), 7.73 (1H, d, *J* = 2.0 Hz, H-5), 7.62 (1H, dd, *J_1_*=8.4 Hz, *J_2_*=2.0 Hz, H-7), 7.35 (1H, d, *J* =8.8 Hz, H-8), 6.48 (1H, d, *J* =9.6 Hz, H-3), 4.60 (2H, s, –SCH_2_–), 4.19 (2H, br, H-a), 3.84 (2H, br, H-d), 2.56–2.54 (5H, m, H-b, H-c, H-e), 1.73–1.70 (4H, m, H-f, H-j), 1.56–1.53 (1H, m, H-h), 1.22–1.00 (5H, m, H-g, H-h, H-i). ^13 ^C NMR (100 MHz, DMSO-d_6_) δ (ppm): 193.7, 179.3, 159.8, 152.6, 143.9, 132.9, 132.8, 128.7, 118.5, 116.4, 62.2, 51.5, 50.2, 48.2, 48.1, 48.0, 29.4, 28.2, 25.7, 25.1 × 2. HRMS (ESI, *m/z*) calcd. for C_21_H_26_N_2_O_2_S_2_ (M + H)^+^ 403.1508, found 403.1509.

##### 2.2.2.17. (2-Oxo-2*H*-chromen-6-yl)methyl 4–(4-hydroxyphenyl)piperazine-1-carbodithioate (**ID-17**)

Yellowish powder. Yield: 55%. m.p: 195.5–196.4 °C. ^1^H NMR (400 MHz, DMSO-d_6_) δ (ppm): 8.91 (1H, s, –OH), 8.04 (1H, d, *J* = 9.6 Hz, H-4), 7.74 (1H, d, *J* = 2.0 Hz, H-5), 7.64 (1H, dd, *J_1_*=8.4 Hz, *J_2_*=2.0 Hz, H-7), 7.36 (1H, d, *J* =8.4 Hz, H-8), 6.80 (2H, d, *J* =8.8 Hz, H-f, H-g), 6.66 (2H, d, *J* =8.8 Hz, H-e, H-h), 6.48 (1H, d, *J* =9.6 Hz, H-3), 4.63 (2H, s, –SCH_2_–), 4.36 (2H, br, H-a), 4.05–3.99 (2H, m, H-d), 3.05 (4H, br, H-b, H-c). ^13 ^C NMR (100 MHz, DMSO-d_6_) δ (ppm): 194.2, 159.8, 152.6, 151.4, 143.9, 143.1, 132.9, 132.8, 128.7, 118.5, 118.3 × 2, 116.4 × 2, 115.4 × 2, 51.0, 50.9, 49.7 × 2, 39.5. HRMS (ESI, *m/z*) calcd. for C_21_H_20_N_2_O_3_S_2_ (M–H)^-^ 411.0843, found 411.0860.

##### 2.2.2.18. (2-Oxo-2*H*-chromen-6-yl)methyl 4–(4-cyanophenyl)piperazine-1-carbodithioate (**ID-18**)

Yellowish powder. Yield: 55%. m.p: 181.5–182.4 °C. ^1^H NMR (400 MHz, DMSO-d_6_) δ (ppm): 8.04 (1H, d, *J* = 9.6 Hz, H-4), 7.74 (1H, d, *J* = 1.6 Hz, H-5), 7.64 (1H, dd, *J_1_*=8.4 Hz, *J_2_*=1.6 Hz, H-7), 7.60 (2H, d, *J* =8.8 Hz, H-f, H-g), 7.36 (1H, d, *J* =8.4 Hz, H-8), 6.94 (2H, d, *J* =8.8 Hz, H-e, H-h), 6.48 (1H, d, *J* = 9.6 Hz, H-3), 4.65 (2H, s, –SCH_2_–), 4.35 (2H, br, H-a), 4.06 (2H, br, H-d), 3.55 (4H, t, *J* =5.2 Hz, H-b, H-c). ^13 ^C NMR (100 MHz, DMSO-d_6_) δ (ppm): 194.4, 159.8, 152.6, 151.9, 143.9, 133.3 × 2, 132.9, 132.8, 128.7, 119.9, 118.5, 116.4, 116.4, 113.3 × 2, 98.1, 50.2, 50.1, 48.6, 48.5, 44.9. HRMS (ESI, *m/z*) calcd. for C_22_H_19_N_3_O_2_S_2_ (M + Na)^+^ 444.0811, found 444.0827.

##### 2.2.2.19. (2-Oxo-2*H*-chromen-6-yl)methyl 4-phenylpiperidine-1-carbodithioate (**ID-19**)

White powder. Yield: 66%. m.p: 115.6–116.5 °C. ^1^H NMR (400 MHz, CDCl_3_) δ (ppm): 7.68 (1H, d, *J* = 9.6 Hz, H-4), 7.58–7.56 (2H, m, H-5, H-7), 7.33–7.27 (3H, m, H-g, H-i, H-8), 7.24–7.18 (3H, m, H-f, H-h, H-j), 6.42 (1H, d, *J* = 9.2 Hz, H-3), 5.75 (1H, br, H-a), 4.72–4.64 (3H, m, –SCH_2_–, H-a), 3.26–3.16 (2H, m, H-e), 2.92–2.84 (1H, m, H-c), 2.00–1.96 (2H, m, H-b), 1.80 (2H, br, H-d). ^13 ^C NMR (100 MHz, CDCl_3_) δ (ppm): 194.9, 160.6, 153.3, 144.2, 143.2, 133.2, 132.9, 128.6 × 2, 128.4, 126.8, 126.7 × 2, 118.7, 117.0, 116.9, 52.6, 50.8, 42.5, 40.8, 33.2, 32.6. HRMS (ESI, *m/z*) calcd. for C_22_H_21_NO_2_S_2_ (M + Na)^+^ 418.0906, found 418.0910.

##### 2.2.2.20. (2-Oxo-2*H*-chromen-6-yl)methyl 4–(4-methylpiperazin-1-yl)piperidine-1-carbodithioate (**ID-20**)

Orange powder. Yield: 66%. m.p: 95.0–96.0 °C. ^1^H NMR (400 MHz, DMSO-d_6_) δ (ppm): 8.04 (1H, d, *J* =9.6 Hz, H-4), 7.73 (1H, d, *J* =2.0 Hz, H-5), 7.62 (1H, dd, *J_1_*=8.4 Hz, *J_2_*=2.4 Hz, H-7), 7.35 (1H, d, *J* =8.8 Hz, H-8), 6.48 (1H, d, *J* =9.6 Hz, H-3), 4.59 (2H, d, *J* = 4.0 Hz, –SCH_2_–), 3.36–3.23 (2H, m, –CH(CH_2_CH_2_)N–(*piperidine*)), 2.58–2.53 (1H, m, –CH(CH_2_CH_2_)N–(*piperidine*)), 2.52–2.49 (2H, m, –CH(CH_2_CH_2_)N–(*piperidine*)), 2.45 (4H, br, –N(CH_2_CH_2_)_2_N–CH_3_–*(piperazine*)), 2.30 (4H, br, –N(CH_2_CH_2_)_2_N–CH_3_–*(piperazine*)), 2.14 (3H, s, –N(CH_2_CH_2_)_2_N–CH_3_), 1.867–1.839 (2H, m, –CH(CH_2_CH_2_)N–(*piperidine*)), 1.38–1.34 (2H, m, –CH(CH_2_CH_2_)N–(*piperidine*)). ^13 ^C NMR (100 MHz, DMSO-d_6_) δ (ppm): 193.2, 159.8, 152.6, 144.0, 133.0, 132.8, 128.7, 118.5, 116.4 × 2, 59.7, 54.9 × 2, 48.3 × 3, 45.5 × 2, 39.8, 28.0, 27.5. HRMS (ESI, *m/z*) calcd. for C_21_H_27_N_3_O_2_S_2_ (M + H)^+^ 418.1617, found 418.1630.

### 2.3. Biological assay

#### 2.3.1. Cell lines and cell culture

Human colorectal cancer cell lines including RKO, SW620 and SW480 were purchased from Chinese Academy of Sciences, a typical cell library culture preservation committee (Shanghai, China). Normal human colon epithelial cell line NCM460 was purchased from ATCC (Washington, DC, USA). RPMI-1640 medium (12633020), foetal bovine serum (FBS, 26170043), penicillin-streptomycin (10378016) and phosphate buffered saline (PBS, 70011044) were purchased from Gibco (Eggenstein, Germany). 5-Fluorouracil (5-FU, E0201010050, purity ≥98%) was purchased from Energy Chemical (Shanghai, China). Dimethyl sulfoxide (DMSO, 41639) was obtained from Sigma-Aldrich (St. Louis, MO). 3–(4,5-dimethylthiazol-2-yl)-5–(3-carboxymethoxyphenyl)-2–(4-sulfophenyl)-2H-tetrazolium (MTS, G1111) was purchased from Promega (Madison, WI, USA). All cell lines were cultured in RPMI-1640 medium supplemented with 10% FBS, penicillin (100 units/mL) and streptomycin (100 mg/mL) at 37 °C in a humidified atmosphere containing 5% CO_2_.

#### 2.3.2. MTS assay

Cell viability was measured via MTS assay. RKO, SW620, SW480 and NCM460 cells were seeded in 96-well plates at a density of 5000 cells per well for 24 h. After treatment with the test compounds at indicated concentration for another 48 h or 72 h at 37 °C, MTS solution was added to each well and incubated for another 30 min at 37 °C. Then, the absorbance at 490 nm was measured by a BioTek Synergy H4 microplate Reader (Vermont, USA). The IC_50_ values were calculated with Graphpad 7.0 software packages.

#### 2.3.3. Binding affinity of target compounds to BRD4(BD1) and BRD4(BD2) by HTRF assay

In brief, compounds were serially diluted in Echo plate according to the plate map and DMSO’s final fraction is 0.1%. Then, transferring compounds and DMSO to 384-well assay plate by Echo. After that, 2× Protein and Peptide Mix was added to the assay plate and 2× Detection Mix was then added to assay plate and shook for 30 s. After incubating the plate at room temperature for 1.5 h, the HTRF signals (Excitation wavelength at 340 nm, Emission wavelength at 615 & 665 nm) was read on EnVision (PerkinElmer, UK). **(+)-JQ1** (BPS Bioscience, Cat. No. 27402) was used as a positive control. BRD4-1 (RD-11–157) and BRD4-2 (RD-11–158) were purchased from Reaction Biology Corp. (PENN, USA). For Curve fitting, the inhibition values was calculated in Excel using Equation (1): Inhibition %=(Max-Signal)/(Max-Min)*100, and the IC_50_ values was calculated in XL-Fit using Equation (2): Y = Bottom + (Top-Bottom)/(1+(IC_50_/X)*HillSlope), Y is %inhibition and X is compound concentration.

#### 2.3.4. Colony formation

SW620 and SW480 cells were seeded in 6-well plates (1000 cells per well) and cultured in RPMI-1640 medium containing 10% FBS overnight. After the cells treatment with DMSO or compound **ID-11** (4, 8, 16, and 32 μM) for 24 h, the culture medium was replaced by fresh RPMI-1640 medium (containing 10% FBS) and continue culturing for another 10 days. Then the cells were washed with PBS and fixed with methanol for 15 min and stained with 0.1% crystal violet for another 30 min. Finally, the cells were washed with running water, air-dried and visualised by photographing.

#### 2.3.5. Cell cycle analysis

Cells were seeded in 6-well plates (40,000 cells per well) and cultured in RPMI-1640 medium containing 10% FBS overnight. Then the cells were exposed to DMSO or compound **ID-11** (8 or 16 μM) for 24 h. After that, cells were collected, washed with cold PBS for twice and fixed in 75% ethanol overnight at 4 °C. Subsequently, ethanol was removed and suspended in PBS for twice, and then RNase A and propidium iodide (PI) (Multi Sciences, Hangzhou, China) was added and keep in the darkness for 30 min. Finally, the percentages of cells in different cell cycle phases were measured using a FACS Calibur flow cytometer (Bectone Dickinson, San Jose, CA, USA).

#### 2.3.6. Cell apoptosis analysis

Cell apoptosis was determined using Annexin V-FITC/PI Apoptosis Detection Kit (BD Biosciences, New Jersey, USA) in according with manufacturer's instruction. Briefly, cells were cultured in 6-well plates (40 000 cells per well) till reaching logarithmic growth phase and then treatment with DMSO or compound **ID-11** (8 or 16 μM) for 24 h. Afterwards, the cells were harvested, washed with PBS for twice and stained with 100 μL of the mixture of Annexin V/FITC and PI in binding buffer (*v/v*, 5: 5: 100) in the darkness for 30 min. Another 200 μL binding buffer was added before detection. The apoptotic cells were then analysed by FACS Calibur flow cytometry immediately.

#### 2.3.7. Western blot assay

Cells were cultured in 6-well plates at a density of 40,000 cells per well overnight, and then exposed to selected compounds at different concentrations for 24 h. Subsequently, the cells were lysed in lysate buffer containing 50 mM Tris-HCl (PH 6.8), 2% SDS, 0.1% bromophenol blue, 1.5% DTT, 10% glycerol. The total protein extracts were boiled, sonicated, separated by 10–12% SDS-PAGE gels and transferred to polyvinylidene fluoride (PVDF) membranes. Then the membranes were blocked with 5% non-fat milk at room temperature for 1 h and incubated with primary antibodies at 4 °C overnight. After that, membranes were washed with 1× TBST for three times and incubated with secondary antibody at room temperature for 2 h. Finally, after washing with 1× TBST for three times, the bolts were visualised by an enhanced chemiluminescence kit (Beyotime, China) using the Amersham Imager 600 system (GE Healthcare Life Science, Shanghai, China).

#### 2.3.8. RNA isolation and quantitative RT-PCR

Cells (50,000 cells per well) incubated in 6-well plates were treated with compound **ID-11** for 24 h. After that, the total RNA was extracted using Total RNA Extraction Reagent (Invitrogen, USA) according to the manufacturer’s instructions and quantified with NanoDrop 2000 (Nanodrop, USA). Then the cDNA was synthesized from total RNA using High-Capacity cDNA Reverse Transcription Kit (Applied Biosystems, USA). The SYBR Green Master reagents (Applied Biosystems, USA) was used to determine the relative expression of mRNA level by quantitative real-time PCR (qRT-PCR). The primer sequences are shown below.

**Table:** 

Gene	Primer sequence (5’-3’)
c-Myc Forward	GCCTCAGAGTGCATCGAC
c-Myc Reverse	TCCACAGAAACAACATCG
Bcl-2 Forward	GTGTGTGGAGAGCGTCAACC
Bcl-2 Reverse	CTTGTGGCCCAGATAGGCA
Actin Forward	ACTCTTCCAGCCTTCCTTCC
Actin Reverse	CGTCATACTCCTGCTTGCTG

#### 2.3.9. Molecular docking

The cocrystal structure of BRD4 with **(+)-JQ1** (PDB: 3MXF) was downloaded from Protein Data Bank (https://www.rcsb.org/) and the whole molecular docking process were carried out in the Ledock software (https://www.lephar.com/). The receptor was prepared using the LePro module with default parameters and a receptor grid was defined as the ligand-binding site based on the cocrystallised ligand and an enclosed box that was in similar in size to the cocrystallised ligand were used to fit the compounds to be docked. The ligand was prepared using ChemBio3D Ultra 14.0 and minimised to the lowest energy. Before the docking study started, the native ligand **(+)-JQ1** was re-docked into the binding site using the same set of parameters as described above. The RMSD of the best-docked pose (binding energy=‒8.66 kcal/mol) was 0.628 Å, thus the docking method using Ledock was suitable. Then the docking work between the receptor and ligand was conducted in the LeDock module and the best docking pose was selected as the dominant conformation. The final docking results were processed with PyMOL software (Version 2.1.0).

#### 2.3.10. Molecular properties prediction of compound ID-11

Molecular properties including absorption, distribution, metabolism and excretion (ADME), toxicity and drug-likeness property of compound **ID-11** were predicted at related available website (https://preadmet.bmdrc.kr/).

#### 2.3.11. Statistic analysis

Data were presented as mean ± standard deviation (SD). One-way ANOVA was used to analyse the statistics by GraphPad Prism 7.0 packages. *p* < 0.05 was considered as statistically significance.

## 3. Results and discussion

### 3.1. Chemistry

**Scheme 1.**
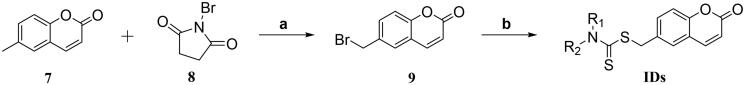
Synthetic routes of novel coumarin-dithiocarbamate derivatives (**IDs**). Reagents and Conditions: (a) CCl_4_, AIBN (cat.), 90 °C, reflux, 10 h; (b) appropriate secondary amines, CS_2_, alkali (KOH, K_2_CO_3_ or K_3_PO_4_), solvent (ethanol, dioxane/H_2_O (1/4, *v/v*) or actone), rt, 12–24 h.

The overall synthetic routes of novel coumarin-dithiocarbamate derivatives (**IDs**) were shown in Scheme 1. Intermediate 6-(bromomethyl)-2*H*-chromen-2-one (**9)** was prepared via the reaction between 6-methyl-2*H*-chromen-2-one (**7**) and 1-bromopyrrolidine-2,5-dione (**8**), and the bromination reaction was catalysed by 2,2′-dimethyl-2,2′-azodipropionitrile (AIBN) with a moderate yield. Then, the final products **ID-1**, **ID-6**, **ID-7**, **ID10**, **ID-12**, **ID-15** and **ID-20** could be obtained via the reactions between intermediate **9**, CS_2_ and appropriate secondary amines in dry ethanol and KOH conditions. Compounds **ID-2** ∼ **ID-5**, **ID-8**, **ID-9**, **ID-13**, **ID-14** and **ID-16** ∼ **ID-19** were prepared with a similar procedure which described for the synthesis of compound **ID-1**, and the solvent was dioxane/H_2_O instead of dry ethanol and the alkali was K_2_CO_3_ instead of KOH. Especially, for the synthesis of compound **ID-11**, actone was used as the solvent and K_3_PO_4_ was used as the alkali. The chemical structures of novel coumarin-dithiocarbamate derivatives (**IDs**) were shown in Table 1. All final products were confirmed by ^1^H NMR, ^13 ^C NMR, and HRMS spectra.

**Table 1.** Structures of novel coumarin-dithiocarbamate derivatives (**IDs**).

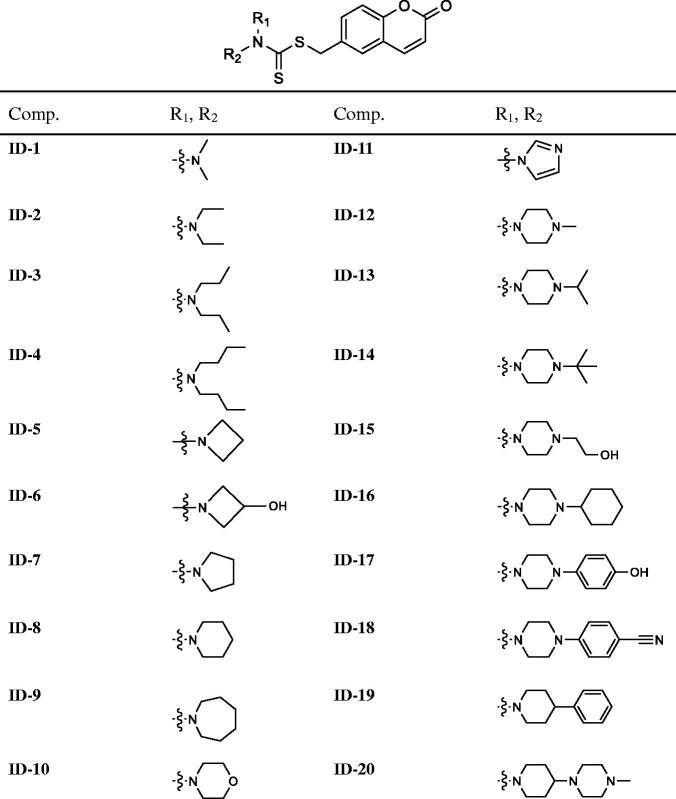

### 3.2. *In vitro* antiproliferative activities of IDs

**Figure 3.**
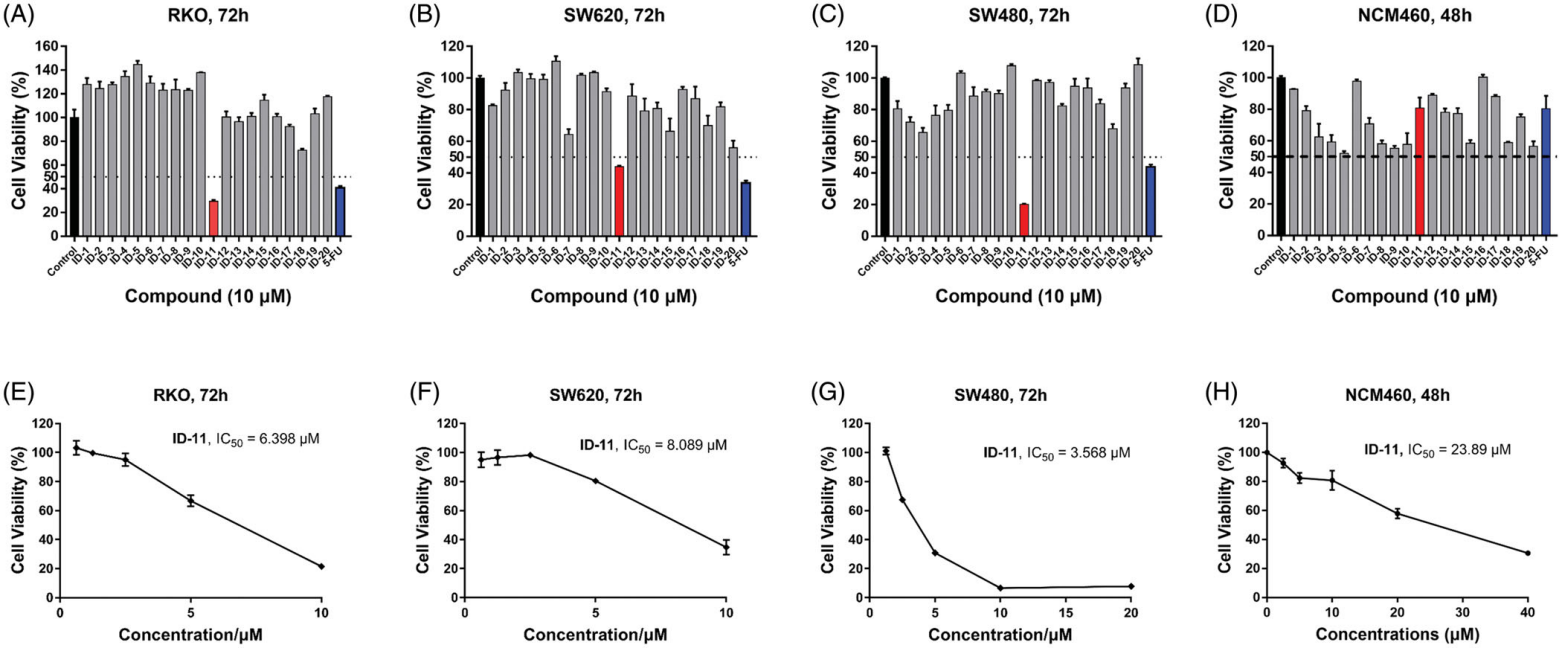
The inhibitory activities of **IDs** against different human CRC cell lines and normal human colon epithelial cells. (A–C) RKO, SW620 and SW480 cells were treated with **IDs** (**ID-1 ∼ ID-20**) and 5-FU for 72 h at 10 μM and then cell viability was determined by MTS assay. (D) NCM460 cells were treated with **IDs** (**ID-1 ∼ ID-20**) and 5-FU for 48 h at 10 μM and then cell viability was determined by MTS assay. (E-G) RKO, SW620 and SW480 cells were treated with **ID-11** for 72 h at variable concentrations (20, 10, 5, 2.5, 1.25, 0.625 μM) and then cell viability determined by MTS assay, finally the IC_50_ values were calculated. (H) NCM460 cells were treated with **ID-11** for 48 h at variable concentrations and then cell viability determined by MTS assay, finally the IC_50_ values were calculated.

**Figure 4.**
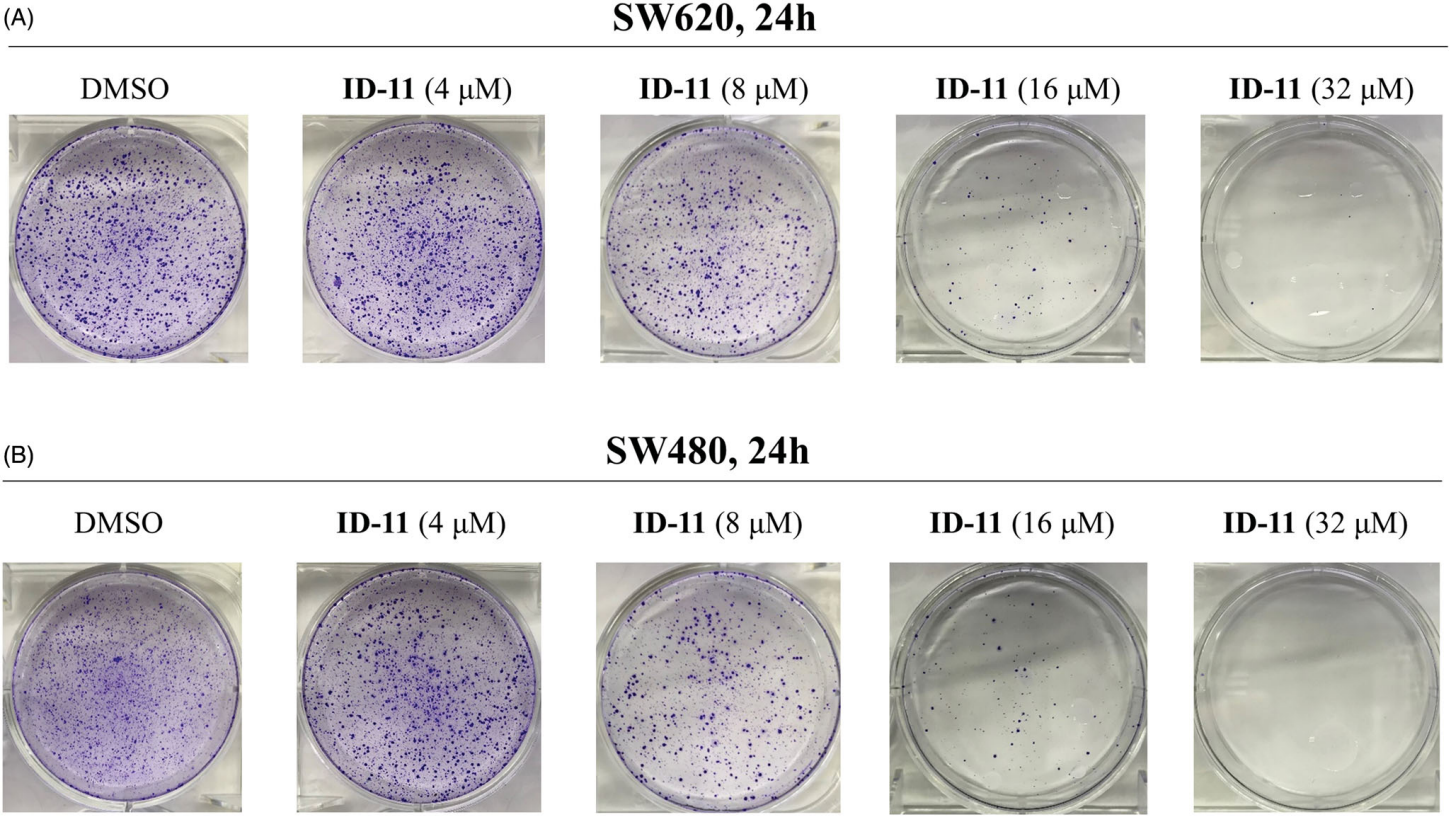
Effects on colony formation of different CRC cell lines by treatment of compound **ID-11**. (A) SW620 cells. (B) SW480 cells. Cells after treating with **ID-11** (0, 4, 8, 16, and 32 μM) for 24 h, continue to cultivate with fresh culture medium for 10 days and then fixed, stained, air-dried and photographed.

To investigate the *in vitro* tumour cell growth inhibitory activities of all newly designed compounds and explore the structure-activity relationships (SAR), three different kinds of human colorectal cancer cell lines, including RKO, SW620 and SW480 were applied. As 5-Fluorouracil (5-FU) is a first-line drug for the treatment of CRC^38^, so we chose it as a positive control in the *in vitro* antiproliferative assay. As shown in Figure 3(A–C), after treatment with the tested compounds (**ID-1** ∼ **ID-20**) at 10 μM for 72 h, the cell viabilities of RKO, SW620 and SW480 cells were all over 50% apart from compound **ID-11** (R_1_, R_2_**=** imidazole**)** treatment group, the cell viabilities of these three CRC cell lines after **ID-11** treatment were 29.43, 43.94, and 20.18%, respectively. At the same condition, the cell viabilities of which were 40.73, 33.57, and 43.87% for 5-FU treatment, respectively. The MTS assay results indicated that, when the R_1_, R_2_ positions replaced by C1–C4 linear alkyl (**ID-1** to **ID-4**), four-membered ring to seven-membered ring secondary amines (**ID-5** to **ID-9**), morpholine (**ID-10**), substituted piperazine or piperidine secondary amines (**ID-12** to **ID-20**) will weaken antiproliferative activities against these cell lines. Next, we measured the IC_50_ values of **ID-11** towards these cancer cell lines. As illustrated in Figure 3(E–G), the IC_50_ values of **ID-11** against RKO, SW620 and SW480 cells were 6.398, 8.809, and 3.568 μM, respectively. Furthermore, we also evaluated the cytotoxicity of these newly designed compounds towards NCM460 cell line (normal human colon epithelial cell line). As shown in Figure 3(D), after incubation with these **IDs** and 5-FU for 48 h at 10 μM, the viabilities of NCM460 cells were all exceed 50% (80.84% for **ID-11** treatment and 80.36% for 5-FU treatment). We also measured the IC_50_ value of **ID-11** against NCM460 cells, and the IC_50_ value of which was 23.89 μM after **ID-11** incubation (Figure 3(H)). We next evaluated the long-term effects on the colony formation of CRC cell lines with the treatment of the most active compound **ID-11**. As shown in Figure 4(A,B), compound **ID-11** effectively inhibited the colony formation of both SW620 and SW480 cells in a concentration dependent way (from 0 to 32 μM). The above results indicated that these **IDs** (especially for **ID-11)** have potential growth inhibitory activities towards CRC cell lines *in vitro* while shown little impact on normal human colon epithelial cells. In view of the excellent antiproliferative activities of **ID-11** against CRC cell lines, **ID-11** was chosen as the representative compound for further anticancer mechanism investigations.

### 3.3. BRD4 inhibitory assay

Growing evidences have shown that the abnormality of BRD4 is closely related to the development of CRC^8^. We want to know whether the antiproliferative activities of these **IDs** towards CRC cell lines was associated with the inhibition of BRD4, so the HTRF assay was performed. In the HTRF assay, reference compound **(+)-JQ1**^11^ was selected as positive control. The results were shown in Table 2. Among all the twenty **IDs**, at the concentration of 10 μM, only compound **ID-11** shown potential inhibitory activity against BRD4 (BD1), the inhibitory rate of which was 68.9%. In the same conditions, **(+)-JQ1** could strongly inhibit both BRD4 (BD1) and BRD4 (BD2), the inhibitory rate of **(+)-JQ1** against BRD4 (BD1) and BRD4 (BD2) were all exceed 98%. We next measured the IC_50_ value of **ID-11** against BRD4 (BD1), and the results shown that **ID-11** inhibited BRD4 (BD1) at μM level, the IC_50_ value of which was 7.8 μM.

**Table 2.** BRD4 inhibitory activities of **IDs** *in vitro*.

Comp.	(Inhibitory rate at 10 μM^a^) or IC_50_ (μM)^b^		cLogP^c^
	BRD4 (BD1)	BRD4 (BD2)	
**ID-1**	(2.2%)	(1.5%)	2.02
**ID-2**	(4.3%)	(5.1%)	3.08
**ID-3**	(1.6%)	(3.3%)	4.14
**ID-4**	(4.2%)	(3.1%)	5.20
**ID-5**	(6.2%)	(5.8%)	2.10
**ID-6**	(15.0%)	(8.7%)	1.32
**ID-7**	(9.2%)	(7.1%)	2.66
**ID-8**	(7.8%)	(8.2%)	3.21
**ID-9**	(6.3%)	(5.0%)	3.77
**ID-10**	(27.2%)	(19.6%)	1.70
**ID-11**	(68.9%)	(35.7%)	2.67
	7.8 ± 2.2		
**ID-12**	(35.5%)	(26.5%)	2.26
**ID-13**	(14.4%)	(10.8%)	3.10
**ID-14**	(12.8%)	(16.6%)	3.50
**ID-15**	(40.2%)	(33.3%)	1.69
**ID-16**	(20.1%)	(12.9%)	4.08
**ID-17**	(39.0%)	(27.7%)	3.03
**ID-18**	(11.8%)	(8.4%)	3.52
**ID-19**	(6.5%)	(3.2%)	4.62
**ID-20**	(36.6%)	(29.3%)	1.04
**(+)-JQ1**	(98.9%)	(99.5%)	4.82
	0.06 ± 0.01	0.03 ± 0.01	

^a^All results were measured three times.

^b^Data were shown as mean ± SD value of three independent experiments.

^c^cLogP values were calculated using ChemDraw 14.0.

**Figure 5.**
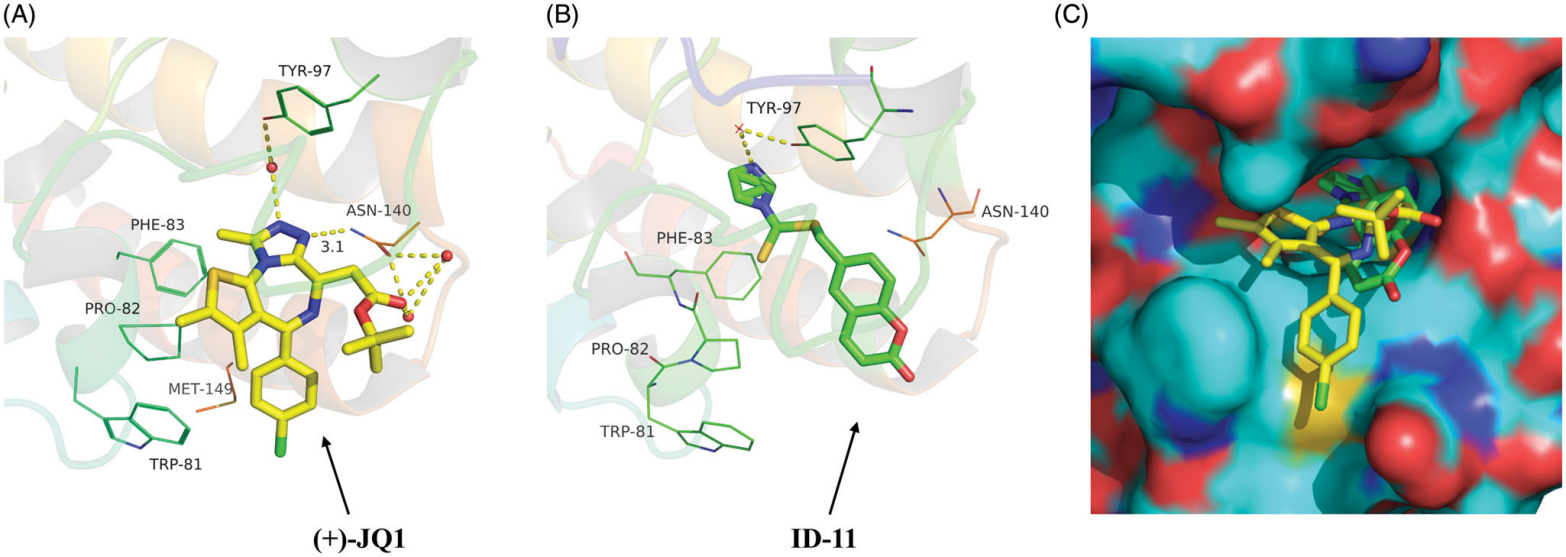
Proposed binding mode of **ID-11** with BRD4. (A) Crystal structure of BRD4 bound to **(+)-JQ1** (yellow stick, in which carbon atoms in yellow, chlorine atom in green, sulphur atom in orange yellow, oxygen atoms in red and nitrogen atoms in blue) (PDB ID: 3MXF); (B) Docking conformation of **ID-11** (green stick, in which carbon atoms in green, sulphur atom in orange yellow, oxygen atoms in red and nitrogen atoms in blue) with BRD4 (PDB ID: 3MXF); (C) Superimposition of **(+)-JQ1** (yellow stick) and **ID-11** (green stick) in their putative bioactive conformations with BRD4 (PDB ID: 3MXF).

Based on the inhibitory activity of compound **ID-11** against BRD4 *in vitro*, we next explored the potential binding mode of **ID-11** with BRD4. As shown in Figure 5(A,B), comparing the best docking conformation of **ID-11** with BRD4 (binding energy = ‒6.59 kcal/mol) and the cocrystal structure of **(+)-JQ1** with BRD4, the N atom of imidazole in **ID-11** formed a hydrogen bond with Tyr-97 residue through a water molecule, which was similar as the binding mode of **(+)-JQ1** with BRD4, while **(+)-JQ1** formed more complex interactions with BRD4, in which the N atom on triazole of **(+)-JQ1** formed another hydrogen bond with ASN-140 (the distance is 3.1 Å) and the carbonyl oxygen of **(+)-JQ1** also formed a hydrogen bond with ASN-140 through a water molecule. In addition, **(+)-JQ1** could also form hydrophobic interactions between the chlorobenzene moiety in **(+)-JQ1** with BRD4. The abundant interactions of **(+)-JQ1** with BRD4 maybe explained the reason why the inhibitory activity of **(+)-JQ1** against BRD4 was much stronger than **ID-11**. As shown in Figure 5(C), we also found that **ID-11** buried more deeply than **(+)-JQ1** in BRD4 binding pocket.

### 3.4. Compound ID-11 induced G2/M cell cycle arrest in human colorectal cancer cells

**Figure 6.**
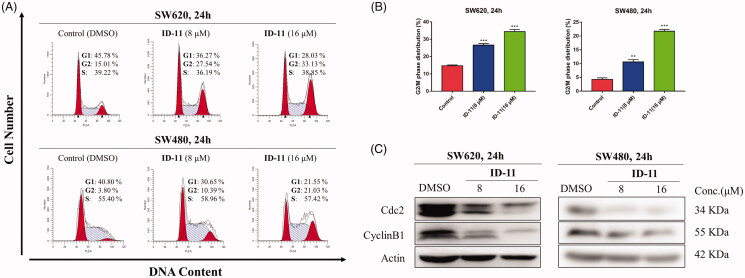
Compound **ID-11** induced G2/M phase arrest in CRC cell lines. (A) SW620 and SW480 cells were incubated with **ID-11** for 24 h at concentration of 0, 8, and 16 μM. Cells were harvested and stained with PI and then cell cycle distributions were measured by flow cytometry. (B) Histogram of G2/M phase distributions. (C) The expression levels of G2/M phase related proteins were determined by western blot assay. Actin was used as the internal control. Error bar original from three independent experiments. (**p < 0.01; ***p < 0.001 vs. control).

Control of cell cycle is important for mediating the equilibrium between cell proliferation and death. Moreover, blockade of the cell cycle regarded as an effective strategy in cancer therapy^39^. In order to explore the compound **ID-11** whether could induce cell cycle arrest in human colorectal cancer cells, flow cytometry assay was performed. As shown in Figure 6(A,B), SW620 and SW480 cells were both arrested at G2/M phase, and the percentage of cell cycle distribution of G2/M phase increased in a concentration-dependent manner (from 15.01 to 33.13% for SW620 cells, and from 3.80 to 21.03% for SW480 cells) after treatment with compound **ID-11** from 0 to 16 μM for 24 h.

Previous studies had revealed that Cdc2 protein kinase plays an important role in cell cycle transition of eukaryotic cells, which was regulated by other factors, such as cyclin B1 and Cdc25c proteins^40^. Thus, western bolt assay was performed to detect the expression level of several cell cycle related proteins in human colorectal cancer cells at the presence or absence of compound **ID-11**. As shown in Figure 6(C), the expression levels of cdc2 and cyclin B1 in SW620 and SW480 cells were both decreased in a concentration-dependent way after exposed to compound **ID-11** from 0 to 16 μM for 24 h.

### 3.5. Compound ID-11 induced apoptosis in human colorectal cancer cells

**Figure 7.**
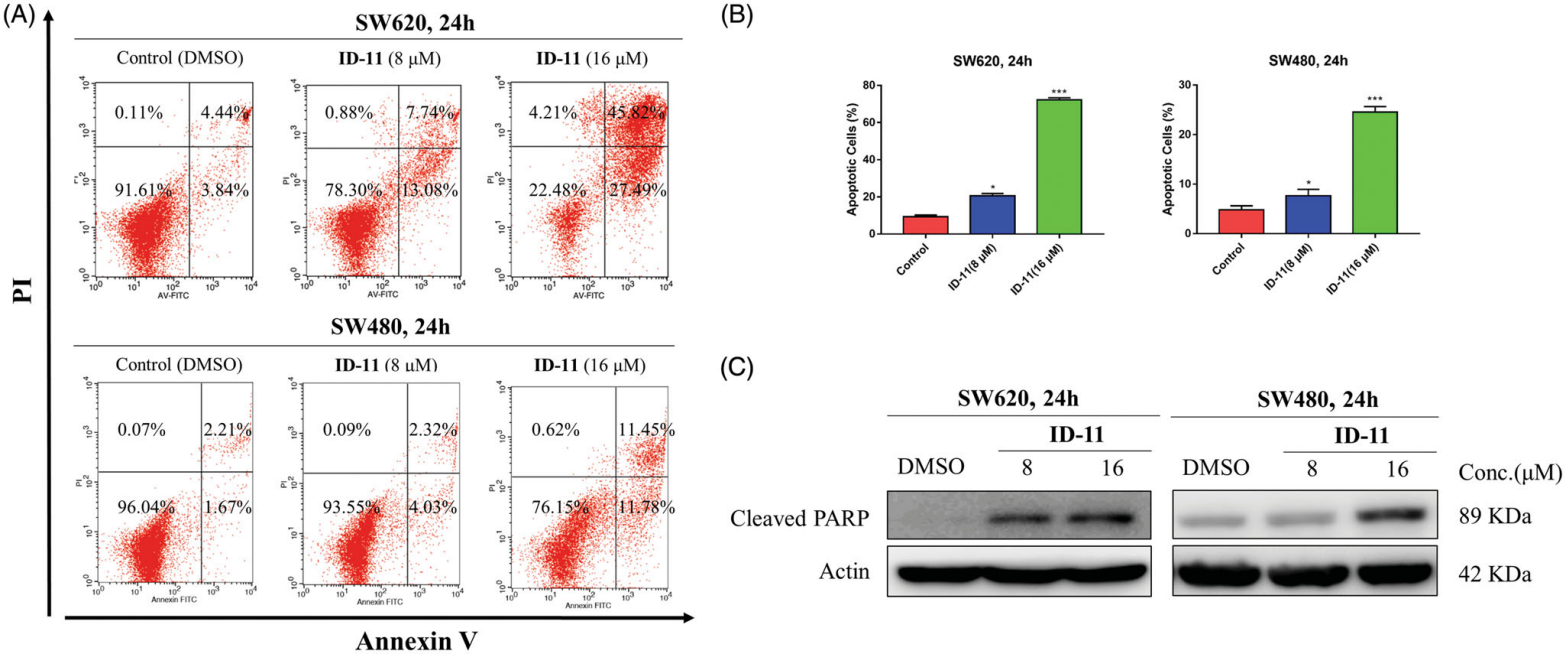
Compound **ID-11** induced apoptosis in CRC cell lines. (A) SW620 and SW480 cells were incubated with **ID-11** for 24 h at concentration of 0, 8, and 16 μM. Cells were harvested and stained with PI/Annexin V and then cell distributions were measured by flow cytometry. (B) Histogram of apoptotic cells. (C) The expression levels of apoptosis-related proteins were determined by western blot assay. Actin was used as the internal control. Error bar original from three independent experiments. (*p < 0.05; ***p < 0.001 vs. control).

Annexin V-FITC/PI staining assay was performed by flow cytometry to investigate whether the antiproliferative potency of compound **ID-11** was consistent with the induction of apoptosis. As shown in Figure 7(A,B), the total apoptosis rate in SW620 and SW480 cells were both dose-dependently increased (from 8.28 to 73.31% for SW620 cells, and from 3.88 to 23.23% for SW480 cells) after incubated with compound **ID-11** from 0 to 16 μM for 24 h.

The process of cell apoptosis was regulated by some vital proteins including, anti-apoptotic proteins (e.g. Bcl-2 and Bcl-xl), pro-apoptotic proteins (e.g. Bax and Bad) and caspase family, etc.^41^. To explore whether the apoptosis effect induced by compound **ID-11** in SW620 and SW480 cells was correlated to the influence on apoptosis-related proteins, western blot assay was performed. The results showed that, the expression level of cleaved PARP was increased in a dose-dependent way in both SW620 and SW480 cells after treatment with compound **ID-11** from 0 to 16 μM for 24 h (Figure 7(C)).

### 3.6. Effects on c-Myc and bcl-2 protein and mRNA expression

**Figure 8.**
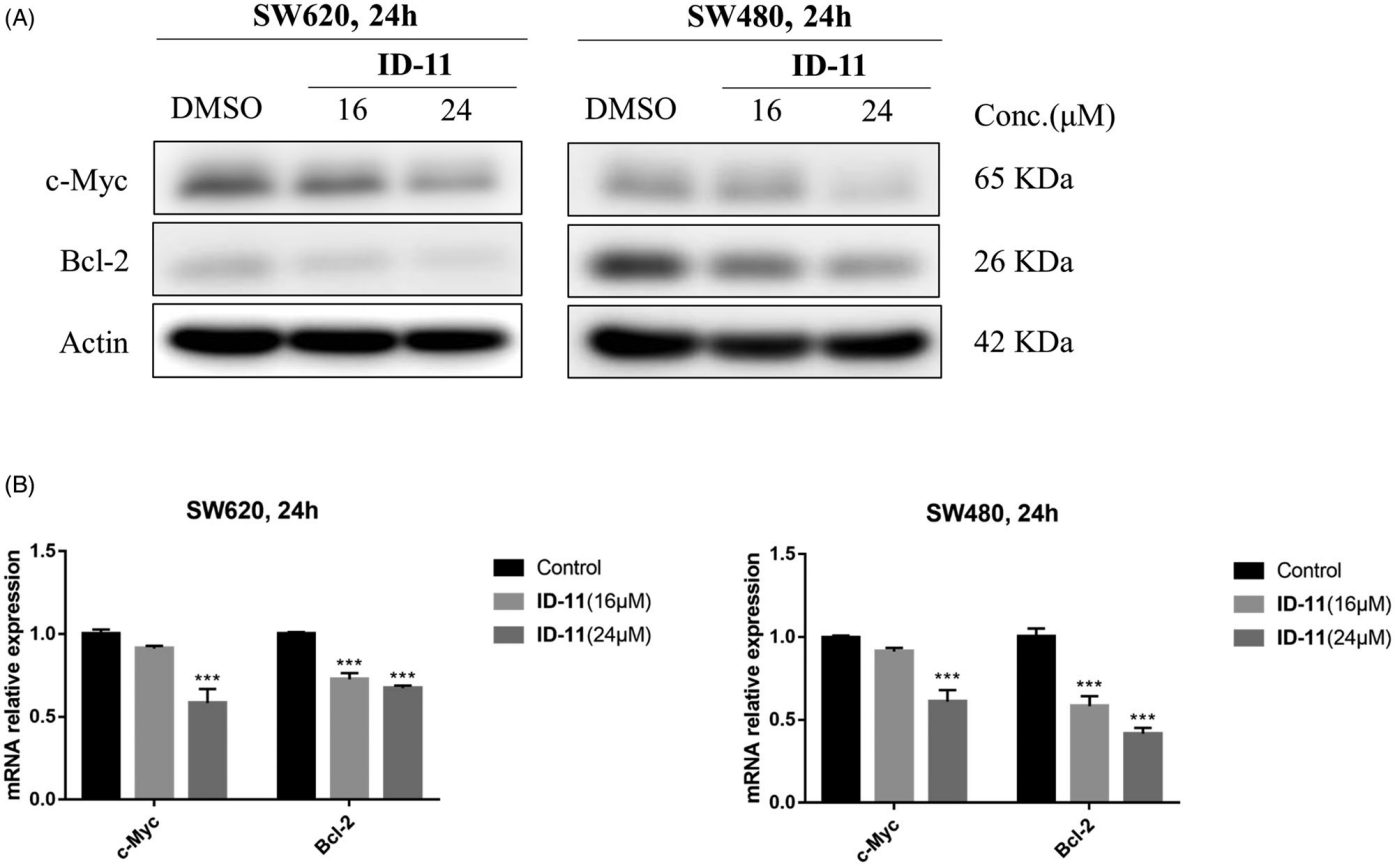
Effects on c-Myc and Bcl-2 protein and mRNA expression in CRC cell lines by treatment of compound **ID-11**. (A) The expression levels of proteins c-Myc and Bcl-2 in SW620 and SW480 cells. Cells after treating with **ID-11** (0, 16, and 24 μM) for 24 h, proteins were extracted and the relative expression levels were determined by western blot assay. (B) The mRNA expression levels of *c-Myc* and *Bcl-2* in SW620 and SW480 cells. Cells after treating with **ID-11** (0, 16, and 24 μM) for 24 h, the total RNA was extracted, quantified and the mRNA levels of *c-Myc* and *Bcl-2* were determined by qRT-PCR.

Previous study shown that the inhibition of BRD4 activity usually affect the expression of some genes such as *c-Myc*, *Bcl-2* and *CDK6*, etc.^42^. Considering the BRD4 inhibitory activity and the ability to induce apoptosis of compound **ID-11** against CRC cell lines, we next measured the protein and mRNA expression levels of c-Myc and Bcl-2 after incubating with **ID-11** in CRC cell lines. As shown in Figure 8(A), **ID-11** treatment group decreased the protein expression levels of c-Myc and Bcl-2 in a concentration dependent way (from 0 to 24 μM) in both SW620 and SW480 cells. Meanwhile, the mRNA relative expression levels of *c-Myc* and *Bcl-2* were also decreased in a concentration dependent way (from 0 to 24 μM) (Figure 8(B)) in both SW620 and SW480 cells.

### 3.7. Molecular properties of compound ID-11

The proper physicochemical properties of a compound are essential for its druggability. Based on the screening results *in vitro*, we further evaluated the physicochemical properties of compound **ID-11**. As shown in Table 3, we predicted the ADMET and drug-likeness properties of **ID-11**, the results indicated that **ID-11** has a high human intestinal absorption rate and plasma protein binding rate without P-glycoprotein inhibitory activity. We also found that **ID-11** follows Lipinski’ rule of five and shown little toxicity. These prediction results shown that **ID-11** owns proper physicochemical properties and well druggability.

**Table 3.** Molecular properties prediction of compound **ID-11**^a^.

ADME		Toxicity		Drug-likeness	
ID	Value	ID	Value	ID	Value
BBB	0.256131	algae_at	0.0613152	CMC_like_Rule	Qualified
Buffer_solubility_mg_L	268.239	Ames_test	Mutagen	CMC_like_Rule_Violation_Fields	–
Caco2	28.7315	Carcino_Mouse	Negative	CMC_like_Rule_Violations	0
CYP_2C19_inhibition	Inhibitor	Carcino_Rat	Negative	Lead-like_Rule_Violation_Fields	AlopP98_value
CYP_2C9_inhibition	Inhibitor	daphnia_at	0.0456393	Lead_like_Rule	Violated
CYP_2D6_inhibition	Non	hERG_inhibition	Medium_risk	Lead_like_Rule_Violations	1
CYP_2D6_substrate	Non	medaka_at	0.00500794	MDDR_like_Rule	Mid-structure
CYP_3A4_inhibition	Inhibitor	minnow_at	0.004834	MDDR_like_Rule_Violation_Fields	No_Rotatable_bonds
CYP_3A4_substrate	Substrate	TA100_10RLI	Positive	MDDR_like_Rule_Violations	1
HIA	99.553241	TA100_NA	Positive	Rule_of_Five	Suitable
MDCK	16.196	TA1535_10RLI	Negative	Rule_of_Five_Violation_Fields	–
Pgp_inhibition	Non	TA1535_NA	Positive	Rule_of_Five_Violations	0
Plasma_Protein_Binding	89.406977			WDI_like_Rule	In 90% cut-off
Pure_water_solubility_mg_L	47.2348			WDI_like_Rule_Violation_Fields	–
Skin_Permeability	−3.7303			WDI_like_Rule_Violations	0
SKlogD_value	1.986330				
SKlogP_value	1.986330				
SKlogS_buffer	−3.052010				
SKlogS_pure	−3.806270				

^a^The predicted results were performed at: https://preadmet.bmdrc.kr/.

## 4. Conclusions

In this work, we provided a series of novel coumarin-dithiocarbamate derivatives (**IDs**) and found that compound **ID-11** shown the best antiproliferative activities towards several human CRC cell lines including RKO, SW620 and SW480 cells, while shown little effect on the normal human colon epithelial cells. Further investigations of the anticancer mechanism indicated that **ID-11** has potential BRD4 inhibitory activity, as well as induced apoptosis and G2/M phase arrest in CRC cells. In addition, **ID-11** also decreased the expression levels of related genes such as *c-Myc* and *Bcl-2*. Furthermore, the prediction results of the physicochemical properties of **ID-11** demonstrated that it may be a promising anti-CRC leading agent and deserved for further development.

## Supplementary Material

Supplemental Material
