# Supplementary material for: Design, synthesis, and evaluation of novel coumarin-dithiocarbamate derivatives (IDs) as anti-colorectal cancer agents
Source: J Enzyme Inhib Med Chem. 2021 Feb 8;36(1):593–604. doi: 10.1080/14756366.2021.1875458 (PMC8759731; doi:10.1080/14756366.2021.1875458)

Supplementary Information

Design, synthesis, and evaluation of novel coumarin-dithiocarbamate derivatives (IDs) as anti-colorectal cancer agents

Heping Zhu[a], Shilong Ying[b], Bingluo Zhou[b], Xinyang Hu[b], Xiao Liang[a], Wangyu Li[a], Dungai Wang[a], Hongchuan Jin*[,b], Yuanjiang Pan*[,a]

[a] Department of Chemistry, Zhejiang University, Hangzhou 310027, P. R. China

[b] Laboratory of Cancer Biology, Key Lab of Biotherapy in Zhejiang, Sir Run Run Shaw Hospital, Medical School of Zhejiang University, Hangzhou 310020, P. R. China

Corresponding authors:

E-mail address: panyuanjiang@zju.edu.cn (YJ. Pan)

E-mail address: jinhc@zju.edu.cn (HC. Jin)

Contents

^{1}H NMR, ^{13}C NMR and HRMS spectra of compounds **ID-1**~**ID-20**……………S2-S25

^{1}H NMR, $CDCl_3$（Bruker AVANCE III 400）（**ID-1**）

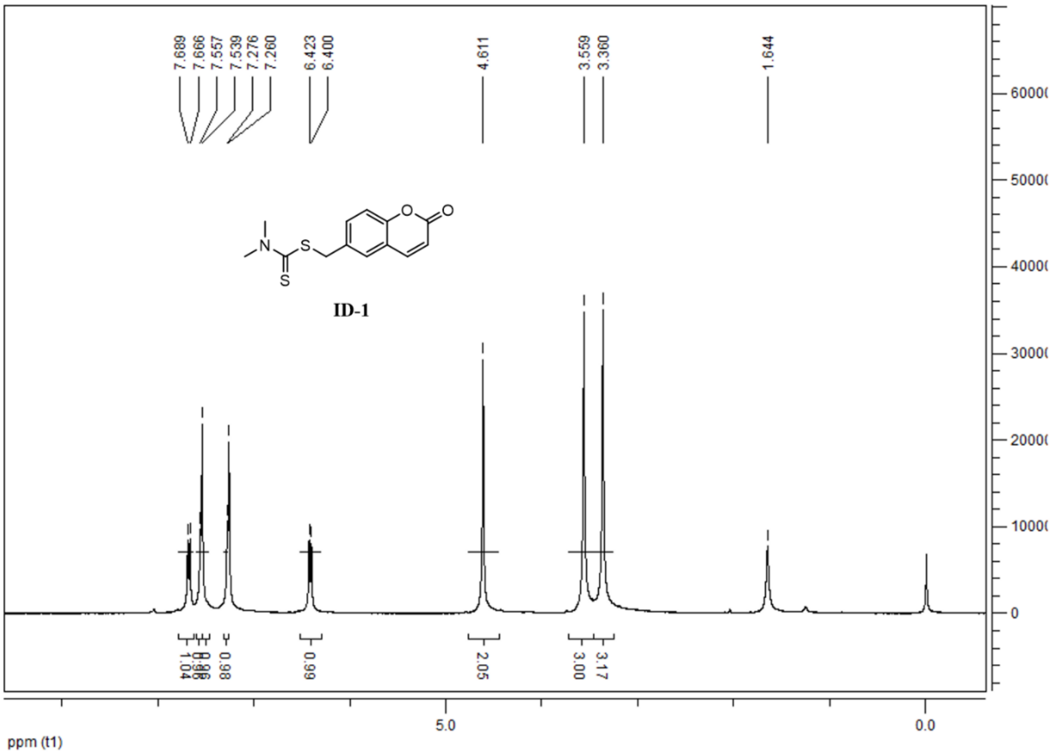

^{13}C NMR, $CDCl_3$（Bruker AVANCE III 400）（**ID-1**）

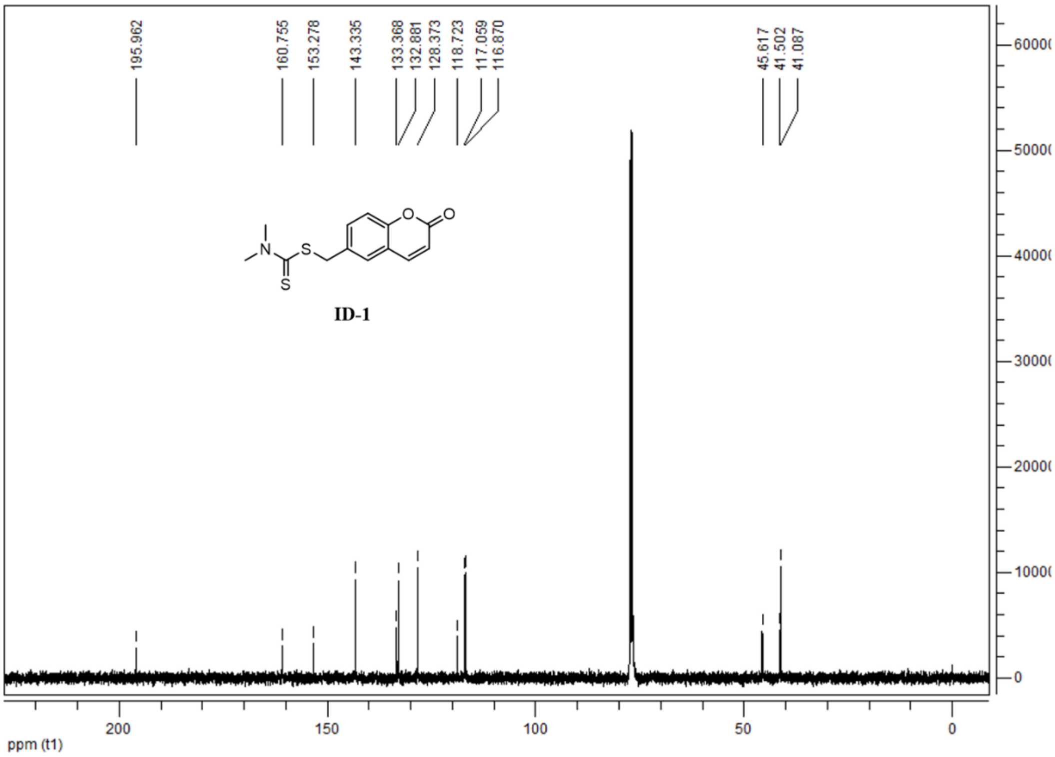

^{1}H NMR, DMSO-d_6（Bruker AVANCE III 400）（**ID-2**）

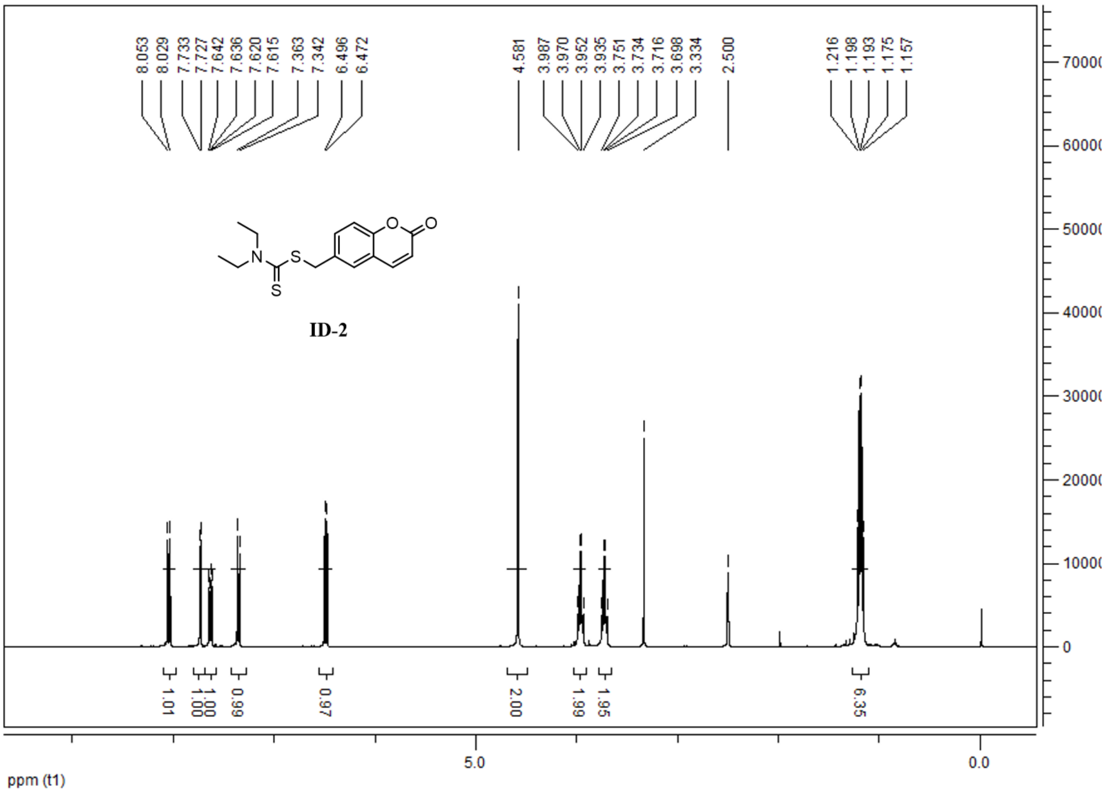

^{13}C NMR, DMSO-d_6（Bruker AVANCE III 400）（**ID-2**）

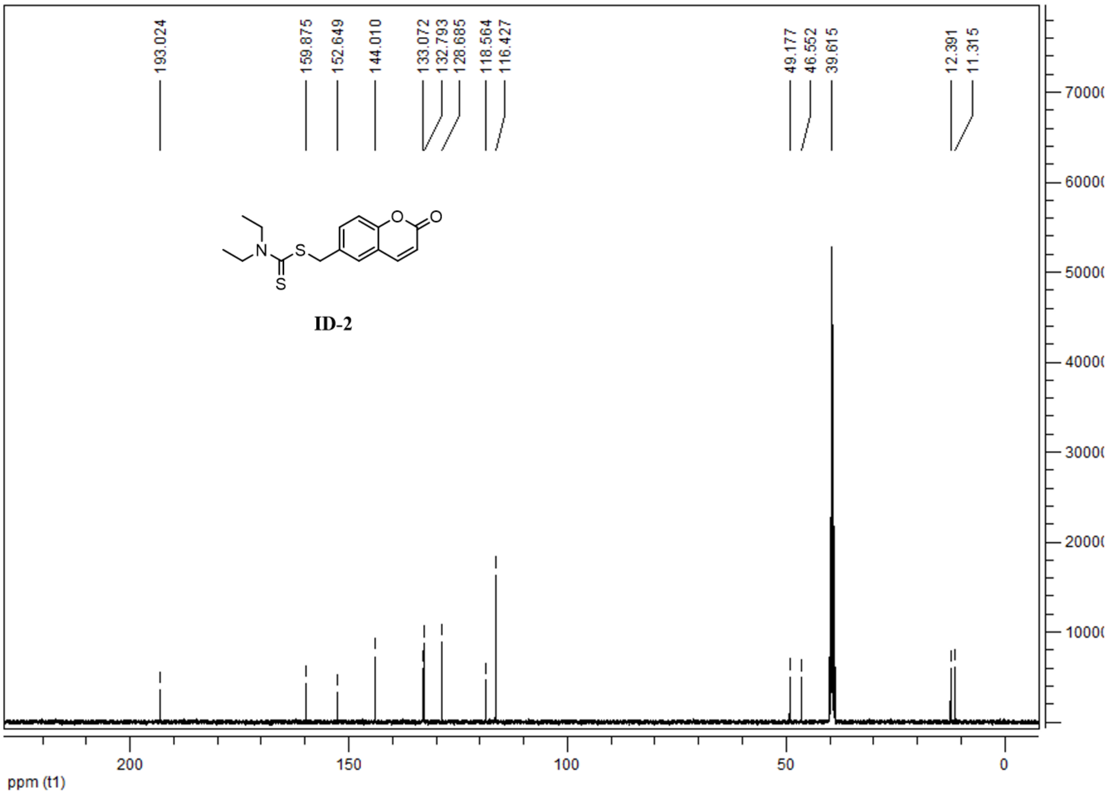

^{1}H NMR, $CDCl_3$（Bruker AVANCE III 400）（**ID-3**）

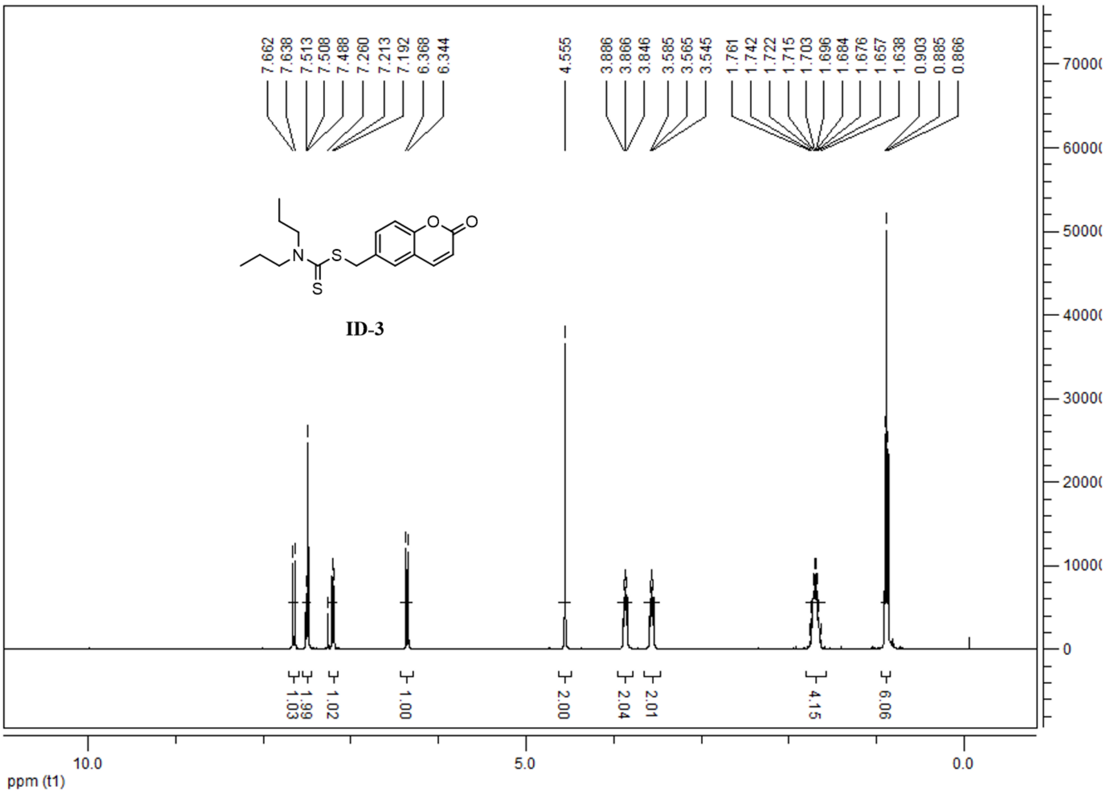

^{13}C NMR, $CDCl_3$（Bruker AVANCE III 400）（**ID-3**）

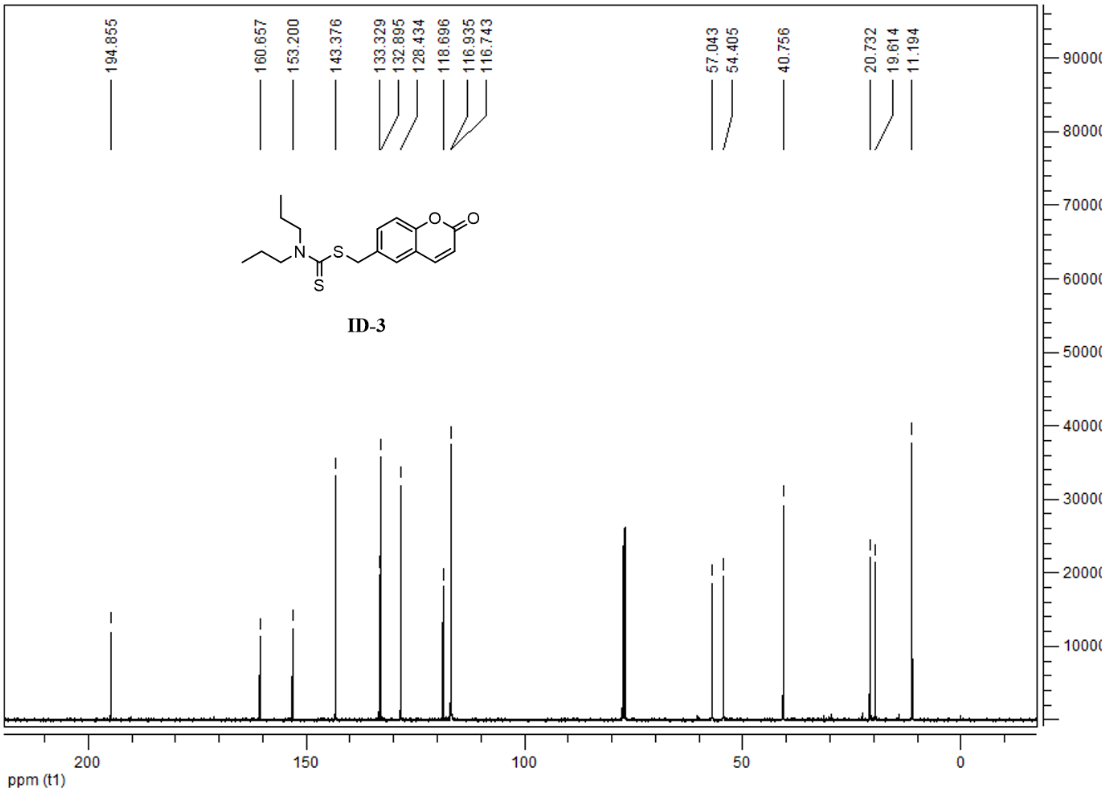

^{1}H NMR, $CDCl_3$（Bruker AVANCE III 400）（**ID-4**）

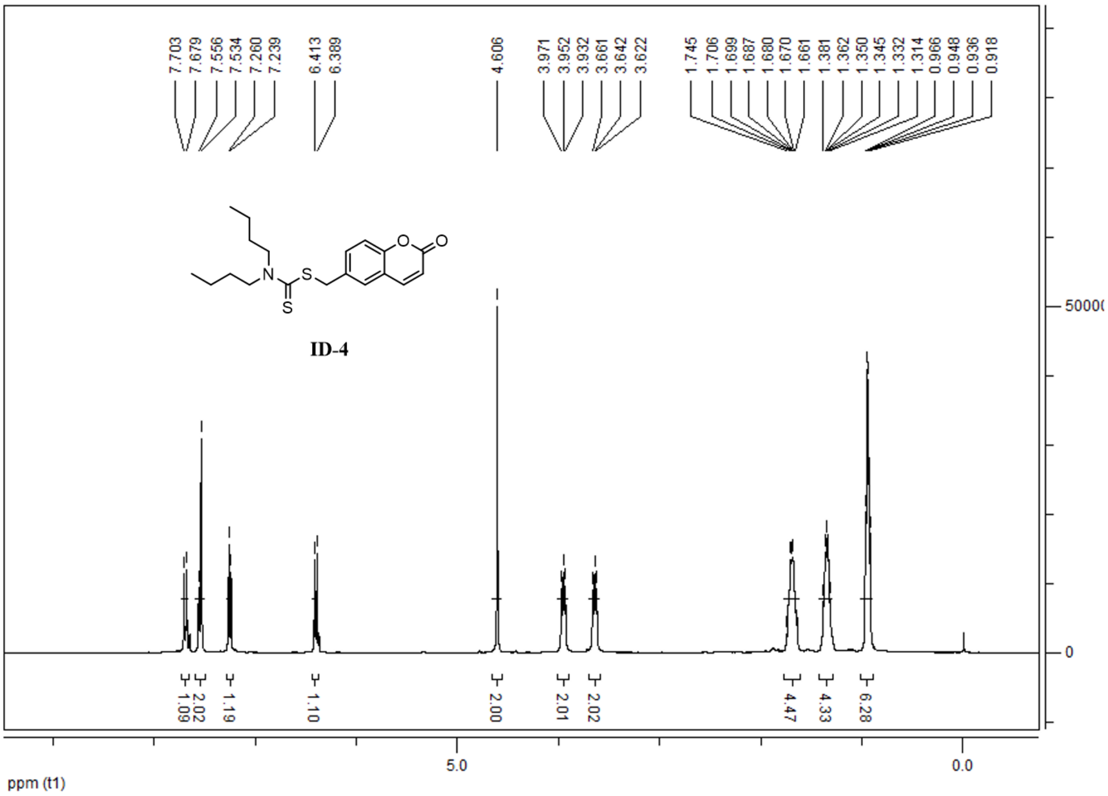

^{13}C NMR, $CDCl_3$ (Bruker AVANCE III 400) (**ID-4**)

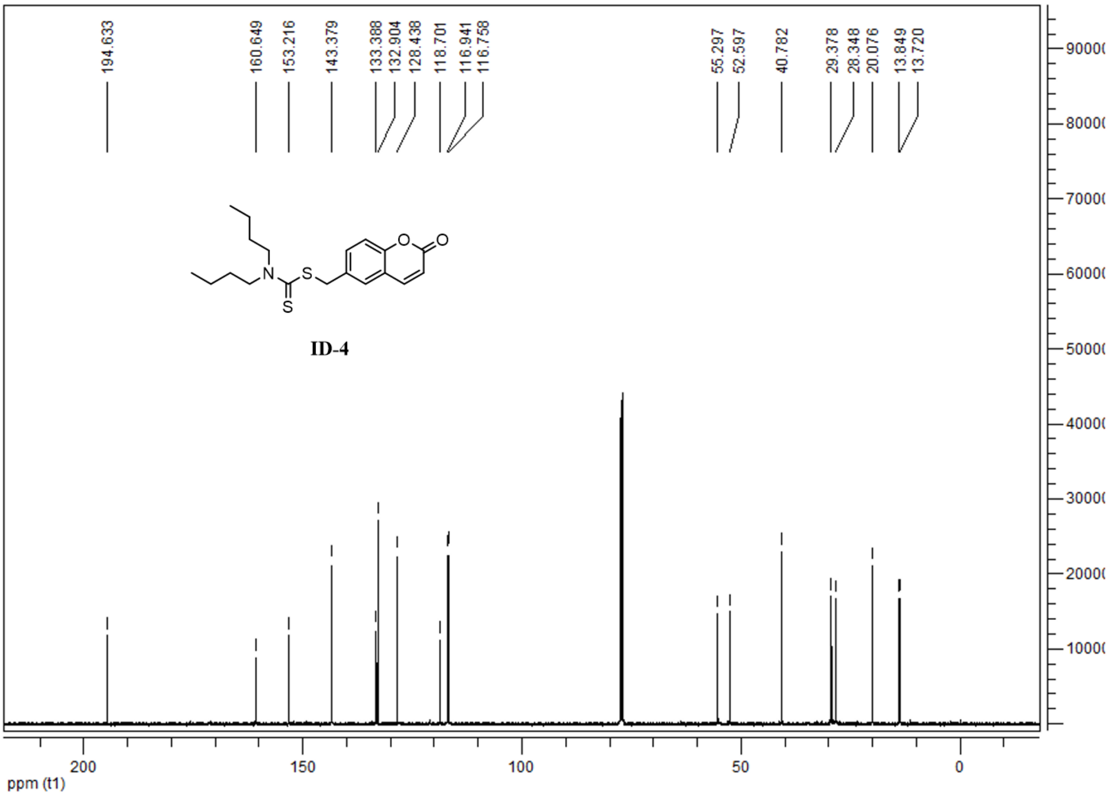

^{1}H NMR, $CDCl_3$ (Bruker AVANCE III 400) (**ID-5**)

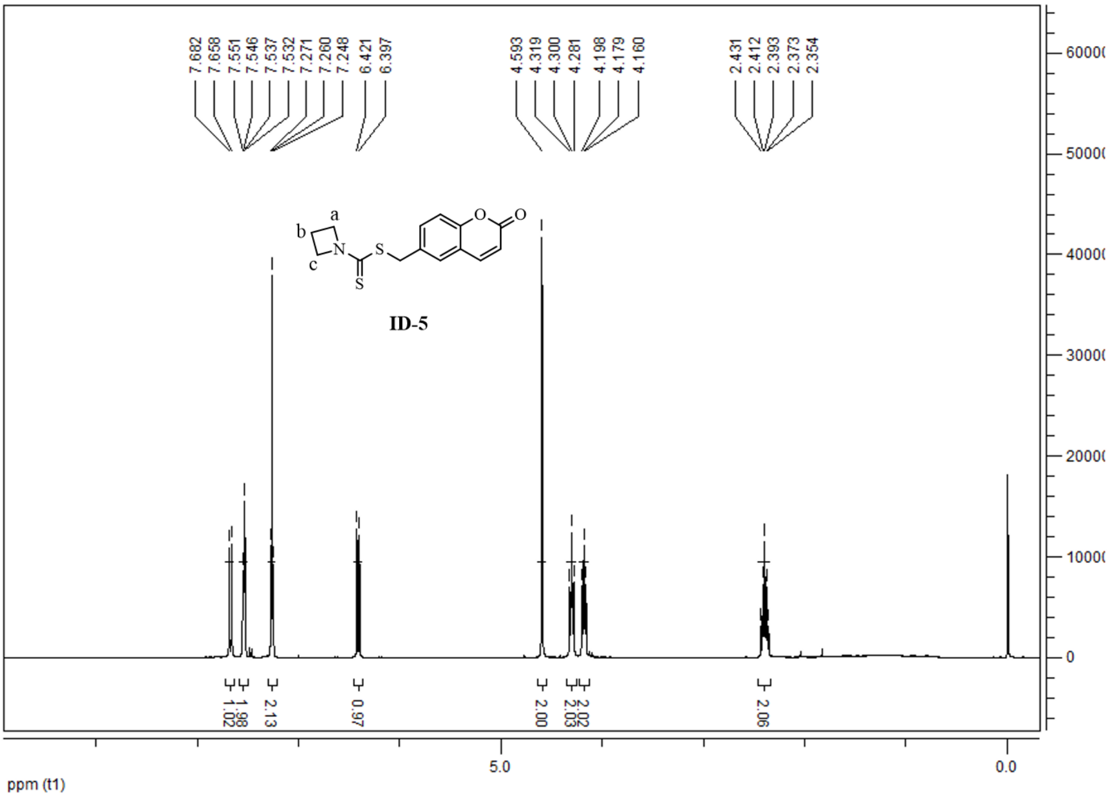

^{13}C NMR, $CDCl_3$ (Bruker AVANCE III 400) (**ID-5**)

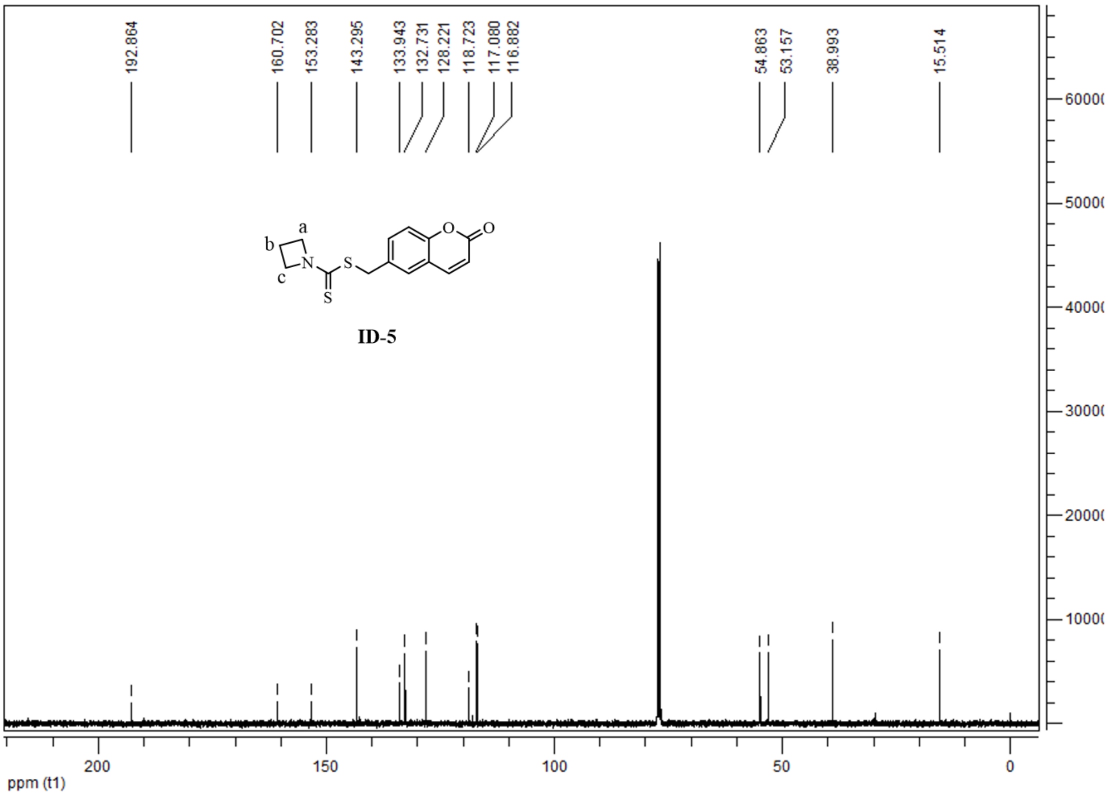

^{1}H NMR, DMSO-d_6 (Bruker AVANCE III 400) (**ID-6**)

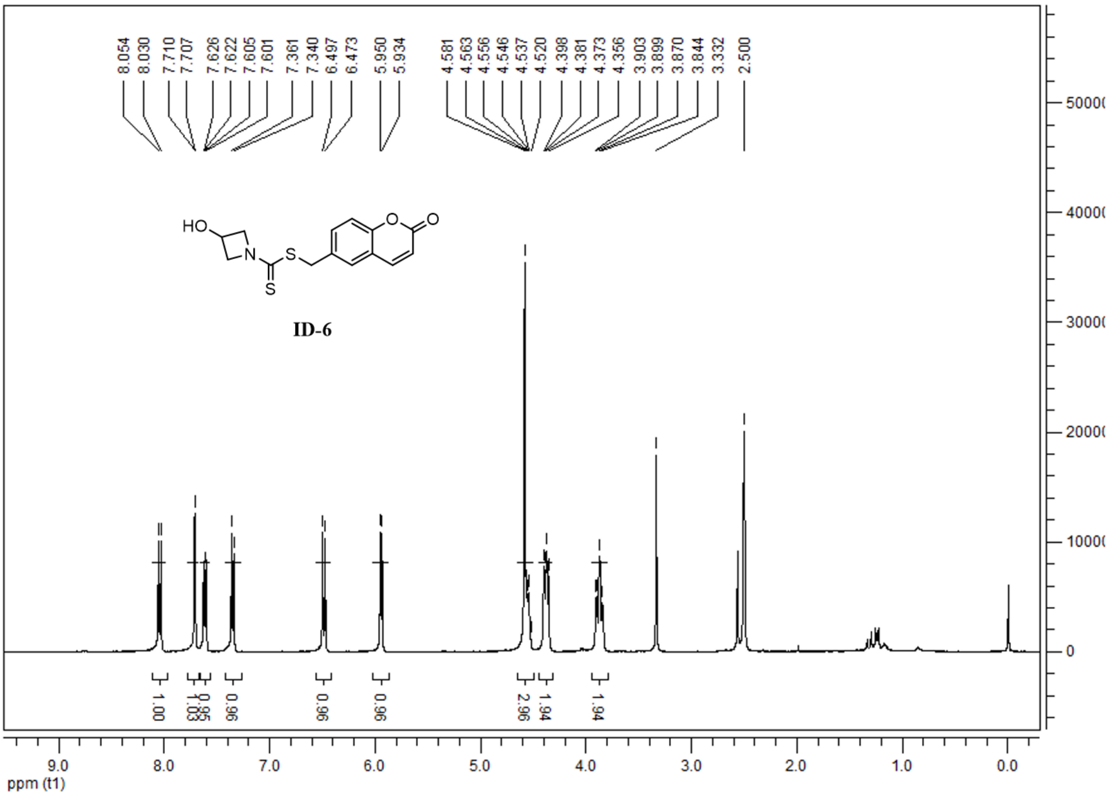

^{13}C NMR, DMSO-d_6 (Bruker AVANCE III 400) (**ID-6**)

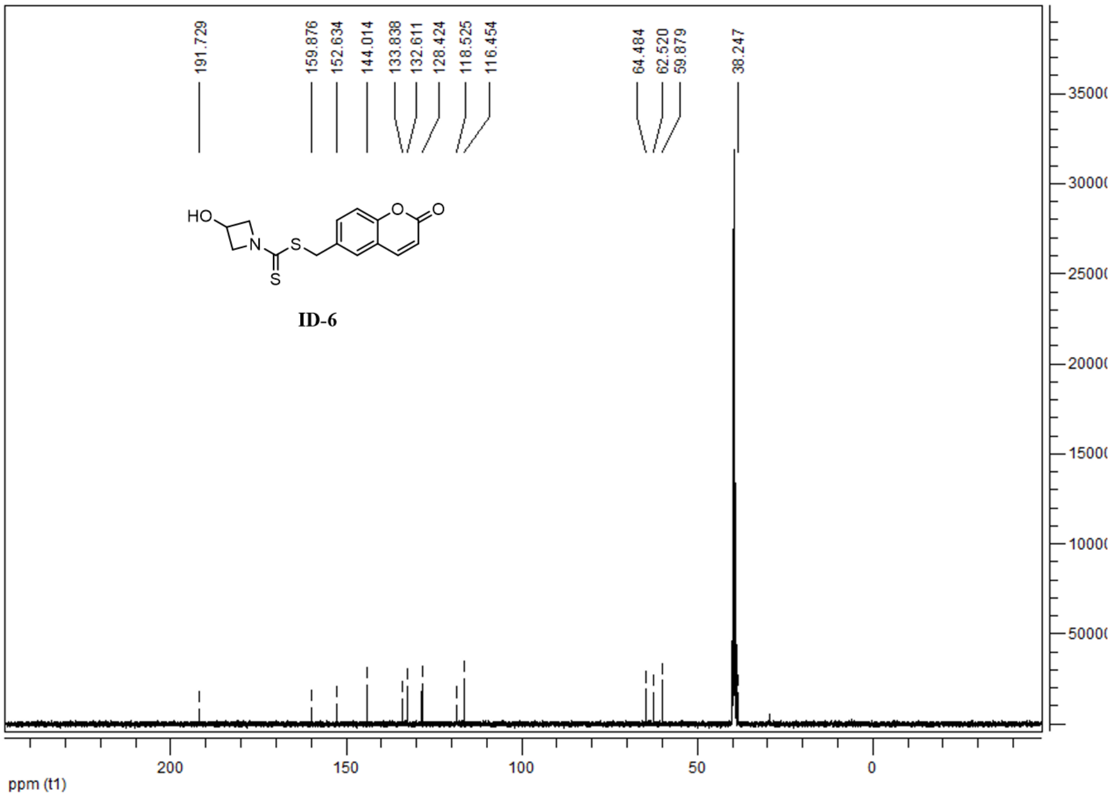

^{1}H NMR, $CDCl_3$ (Bruker AVANCE III 400) (**ID-7**)

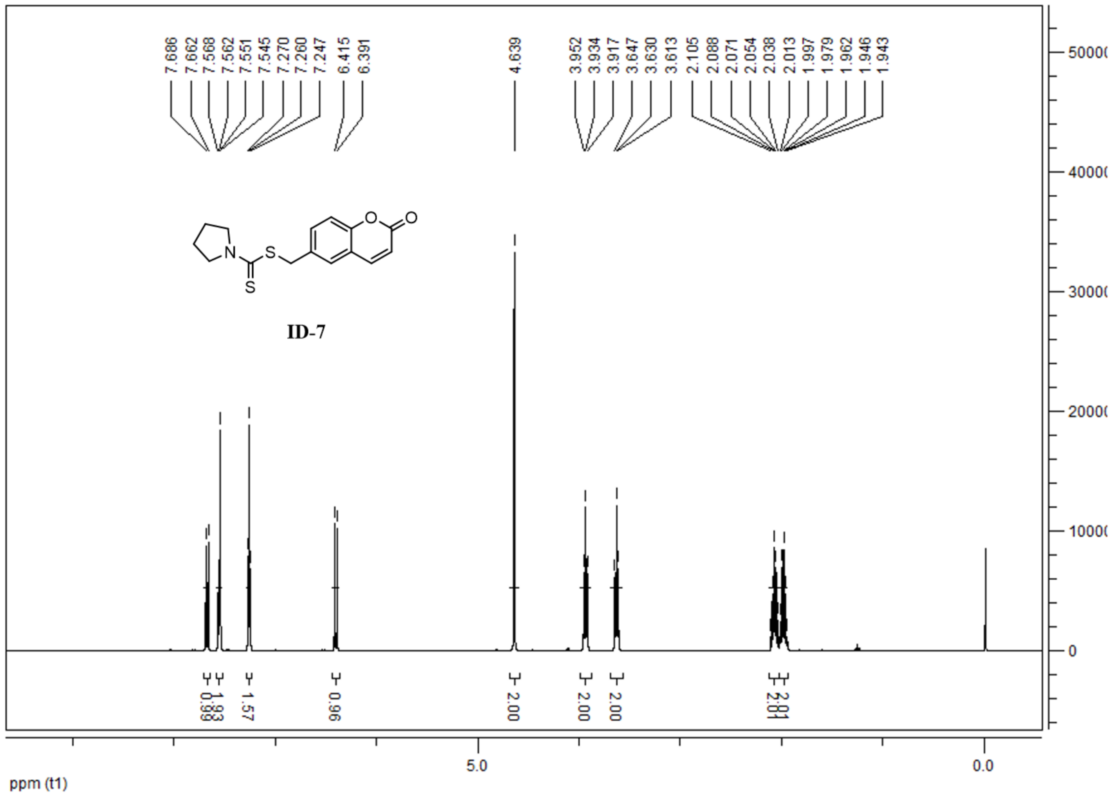

^{13}C NMR, $CDCl_3$（Bruker AVANCE III 400）（**ID-7**）

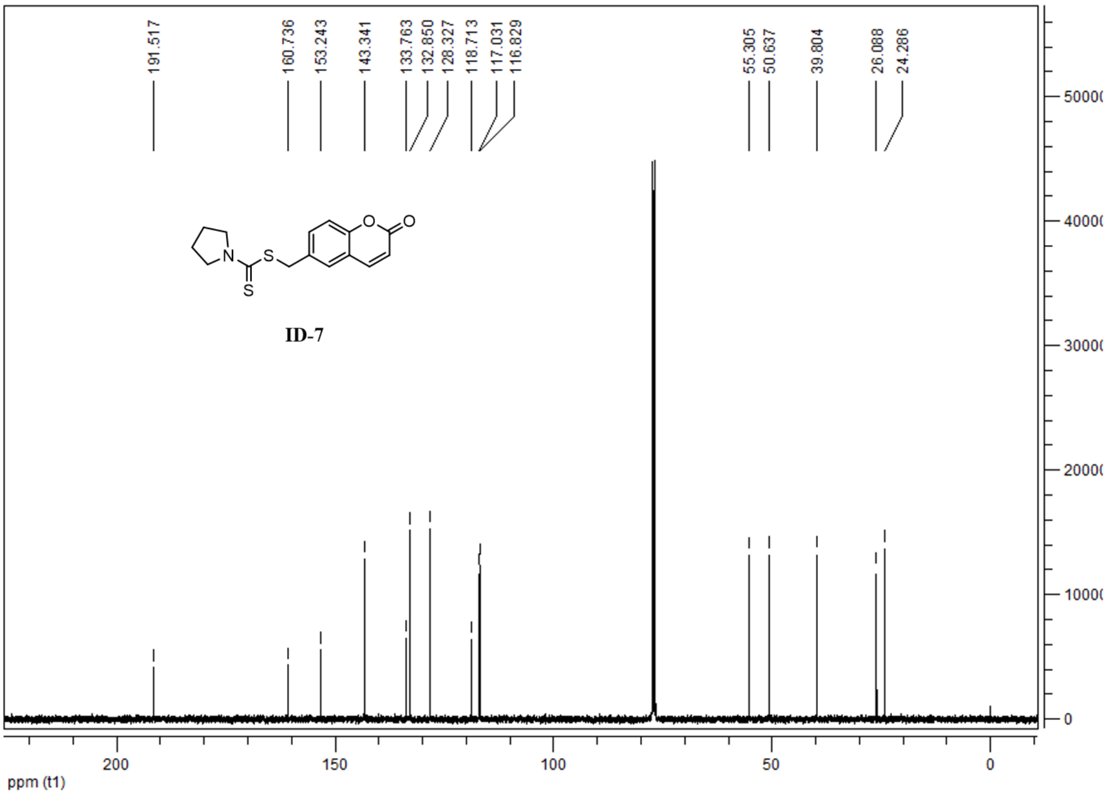

^{1}H NMR, $CDCl_3$（Bruker AVANCE III 400）（**ID-8**）

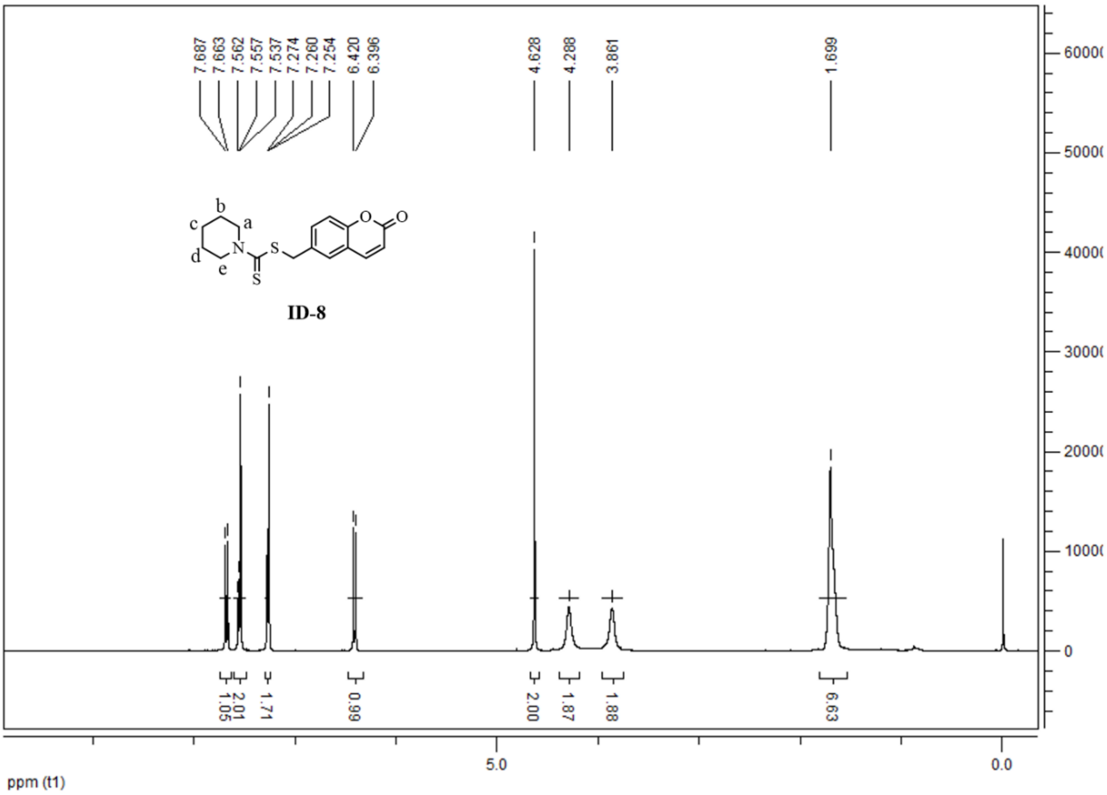

^{13}C NMR, $CDCl_3$（Bruker AVANCE III 400）（**ID-8**）

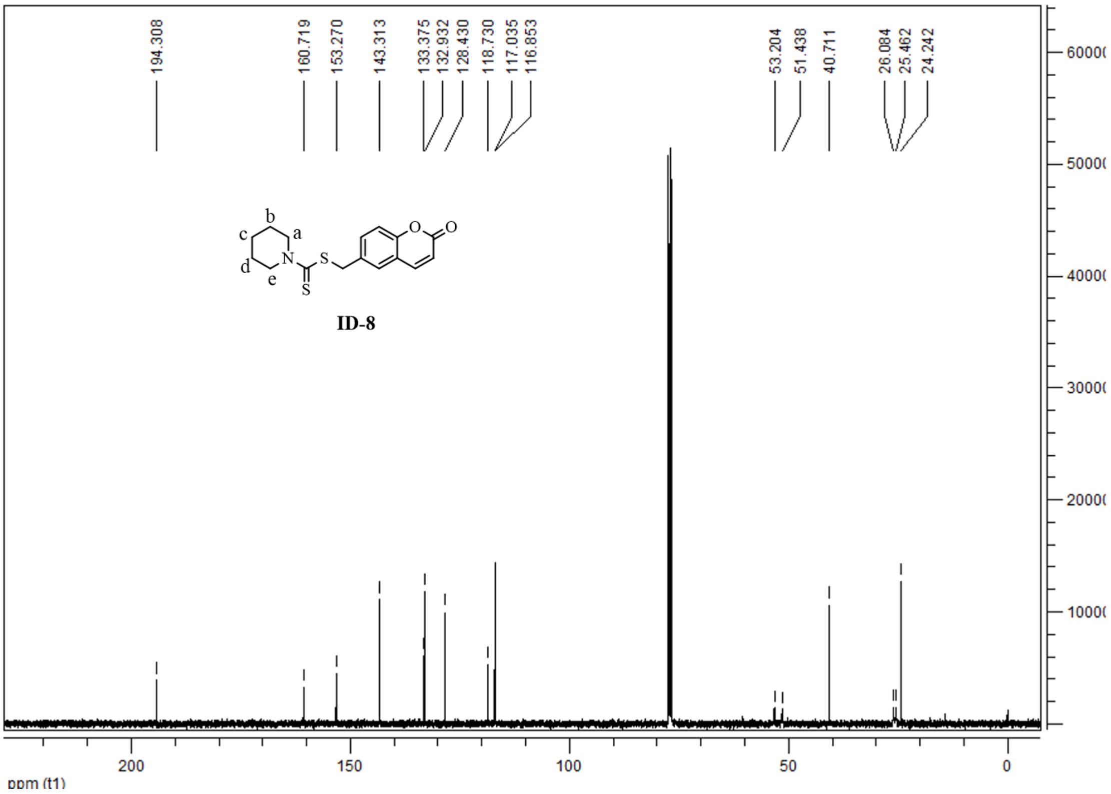

^{1}H NMR, $CDCl_3$（Bruker AVANCE III 400）（**ID-9**）

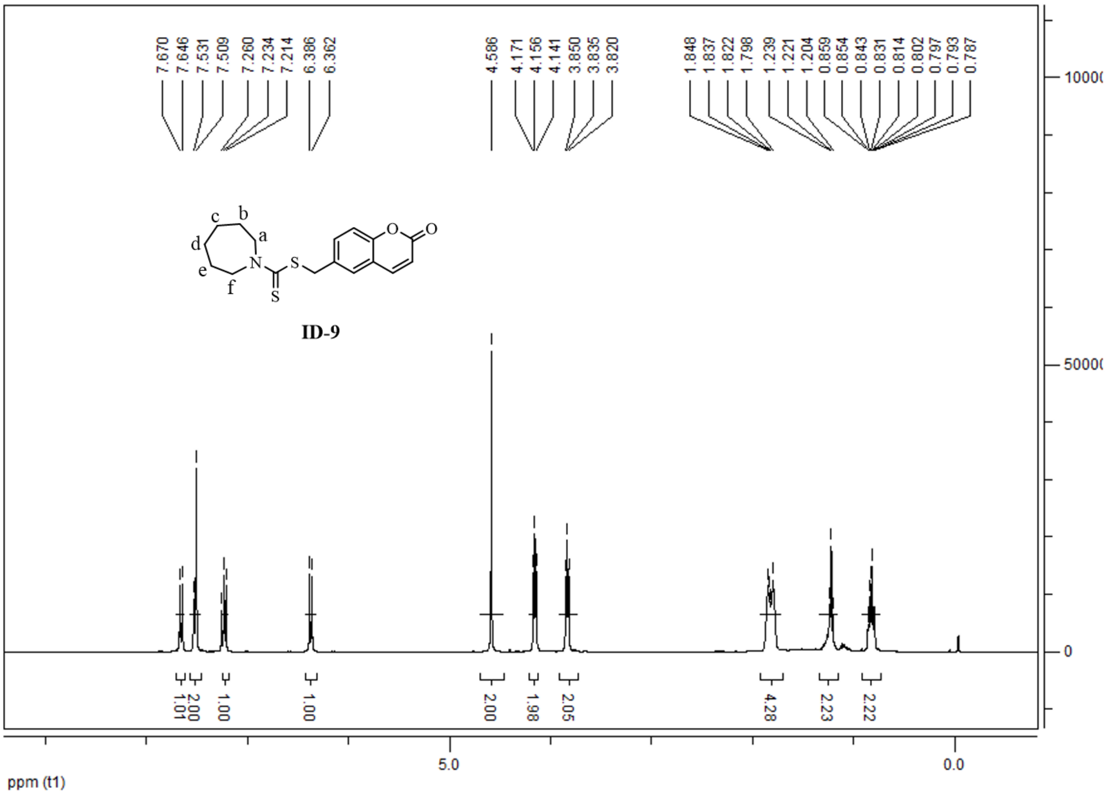

^{13}C NMR, $CDCl_3$ (Bruker AVANCE III 400) (**ID-9**)

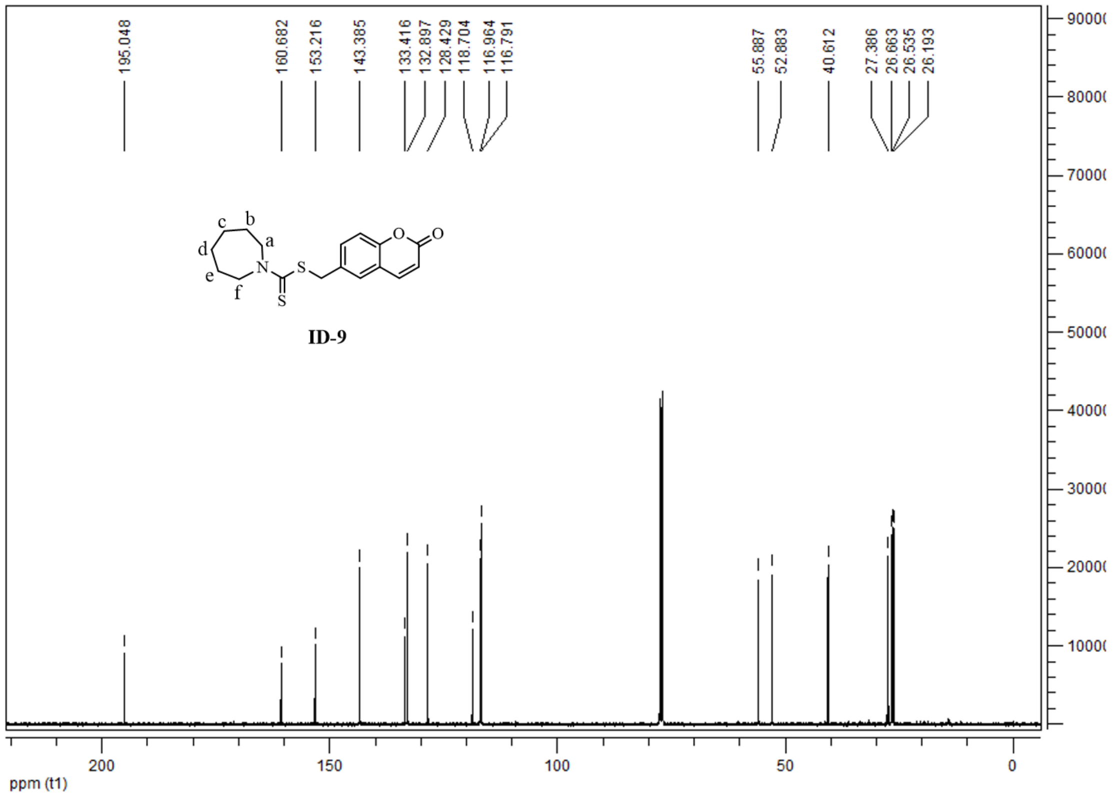

^{1}H NMR, DMSO-d_6 (Bruker AVANCE III 600) (**ID-10**)

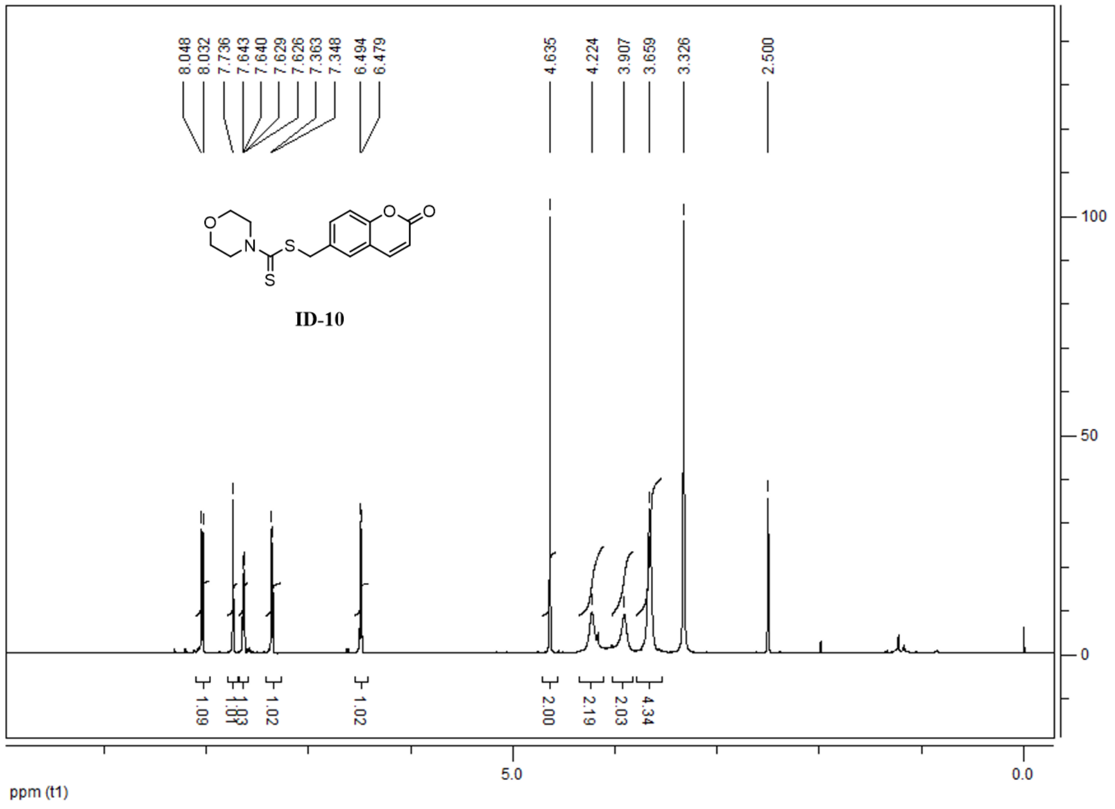

^{13}C NMR, DMSO-d_6（Bruker AVANCE III 600）（**ID-10**）

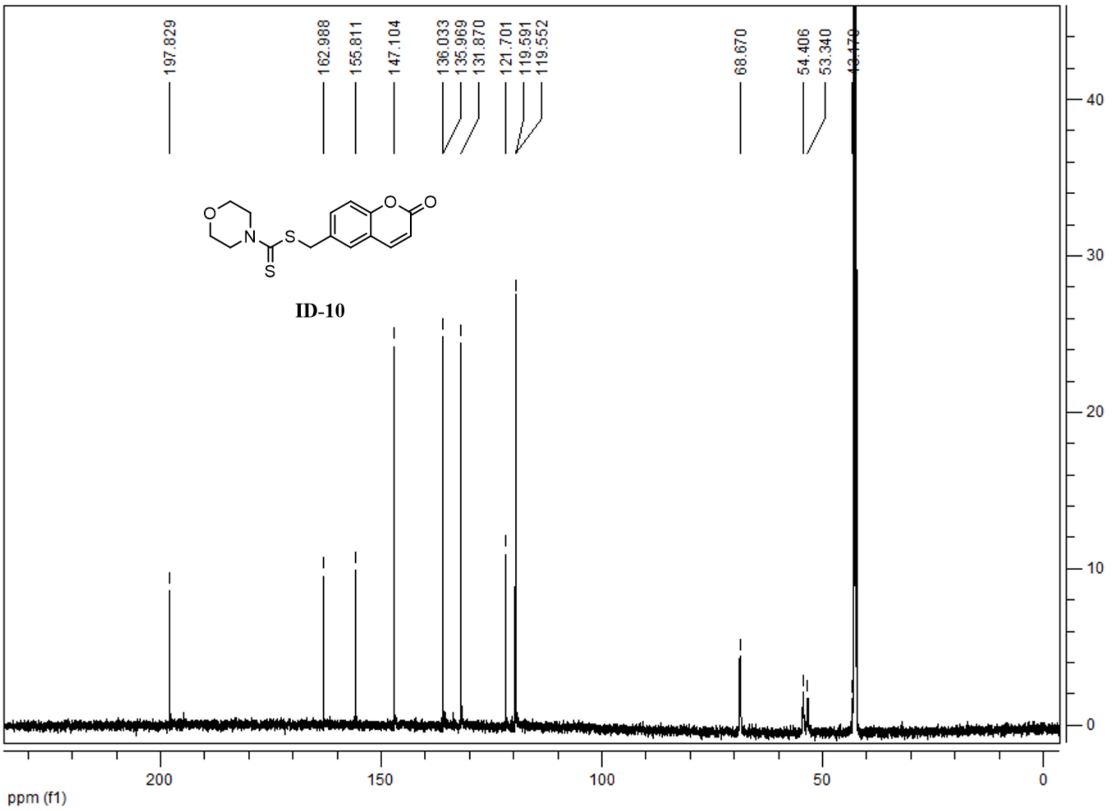

^{1}H NMR, DMSO-d_6（Bruker AVANCE III 400）（**ID-11**）

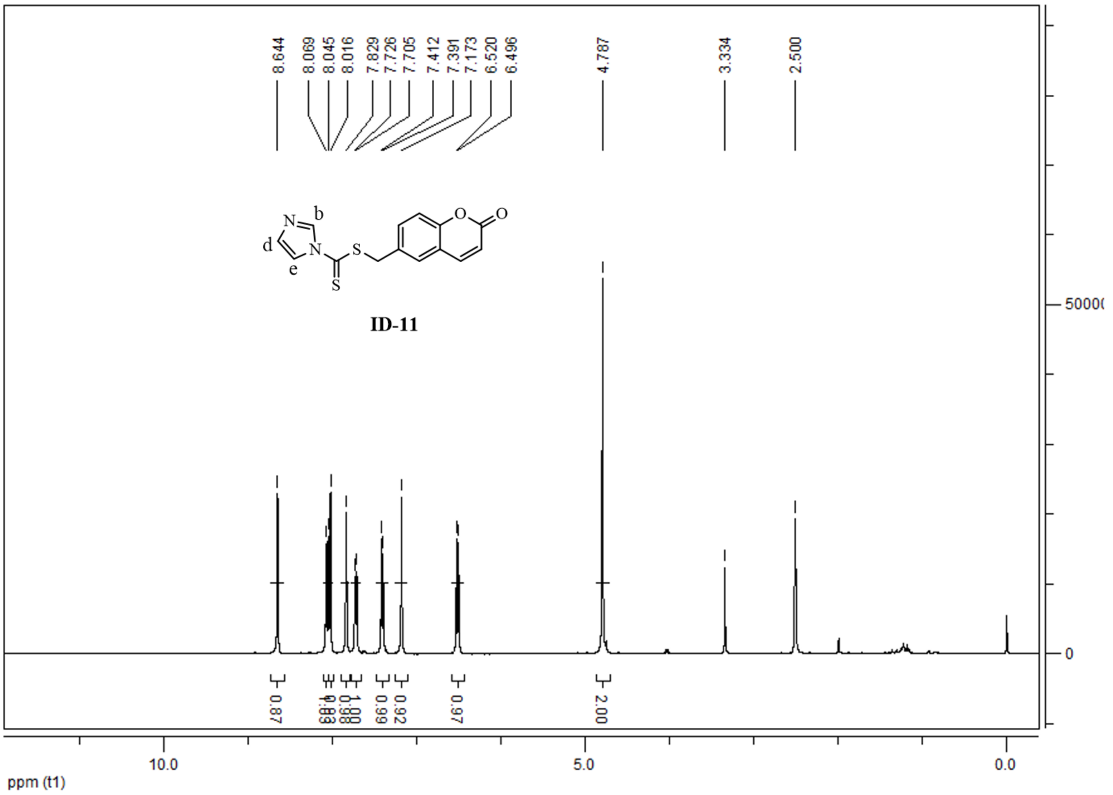

^{13}C NMR, DMSO-d_6（Bruker AVANCE III 400）（**ID-11**）

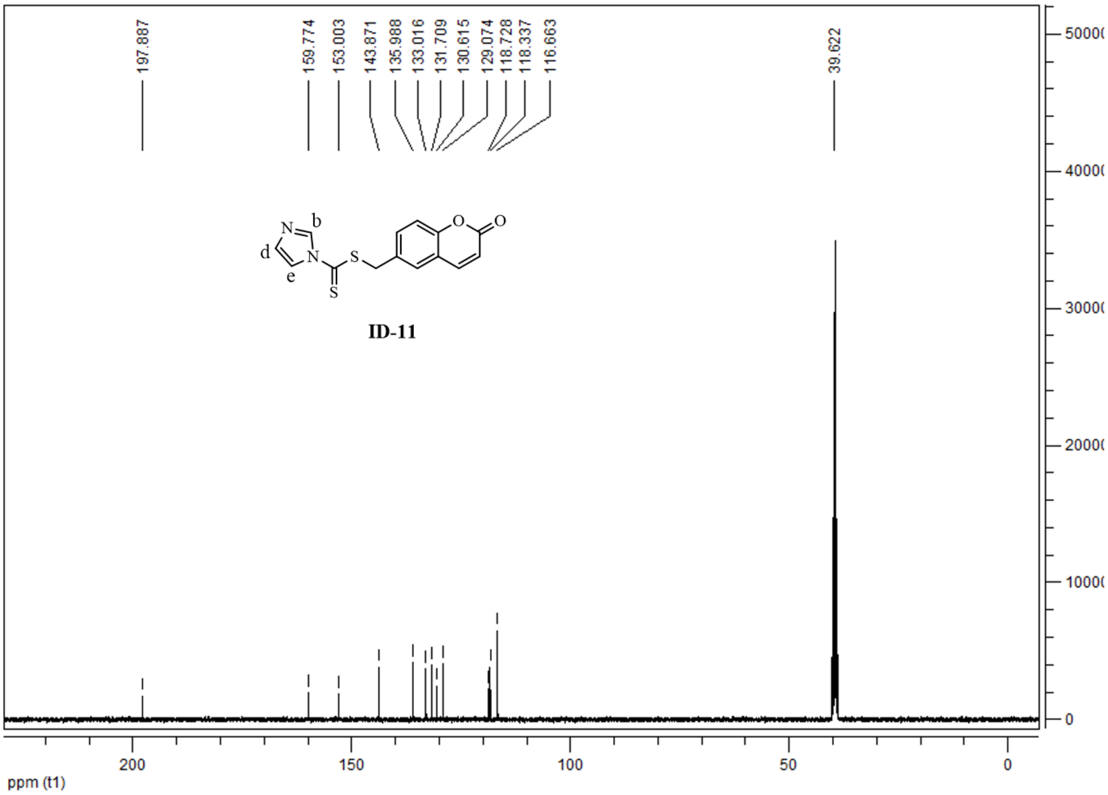

^{1}H NMR, DMSO-d_6（Bruker AVANCE III 400）（**ID-12**）

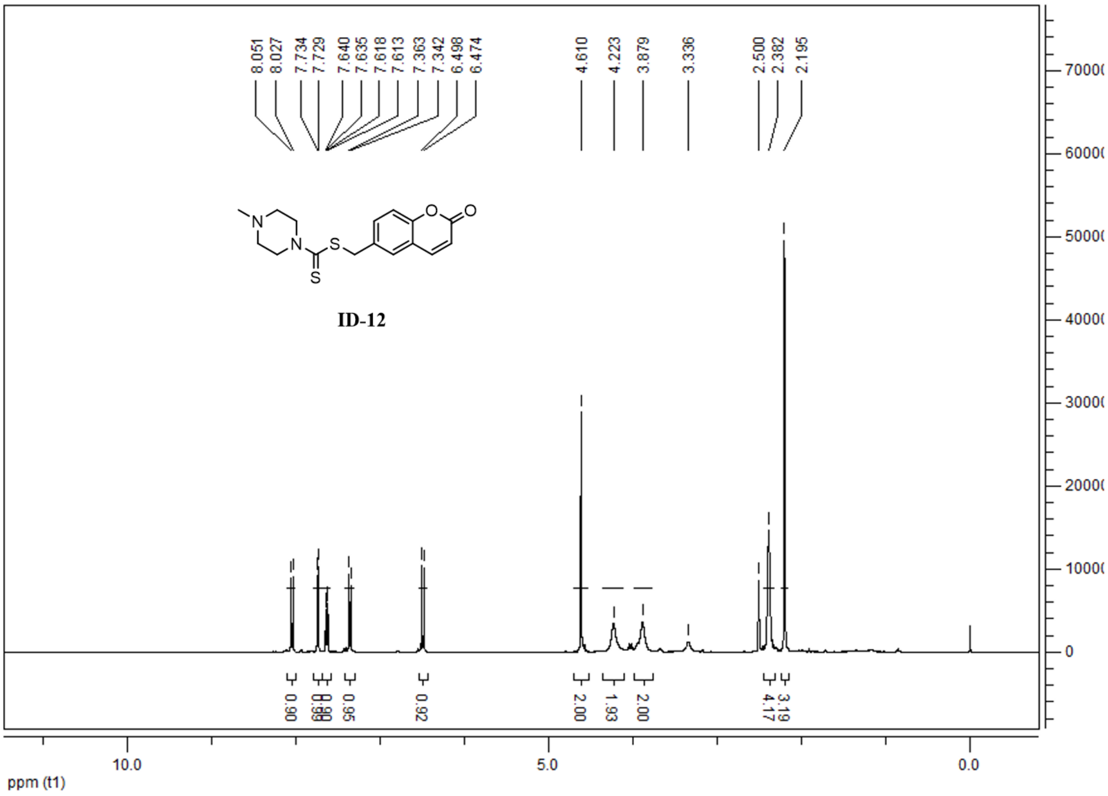

^{13}C NMR, DMSO-d_6（Bruker AVANCE III 400）（**ID-12**）

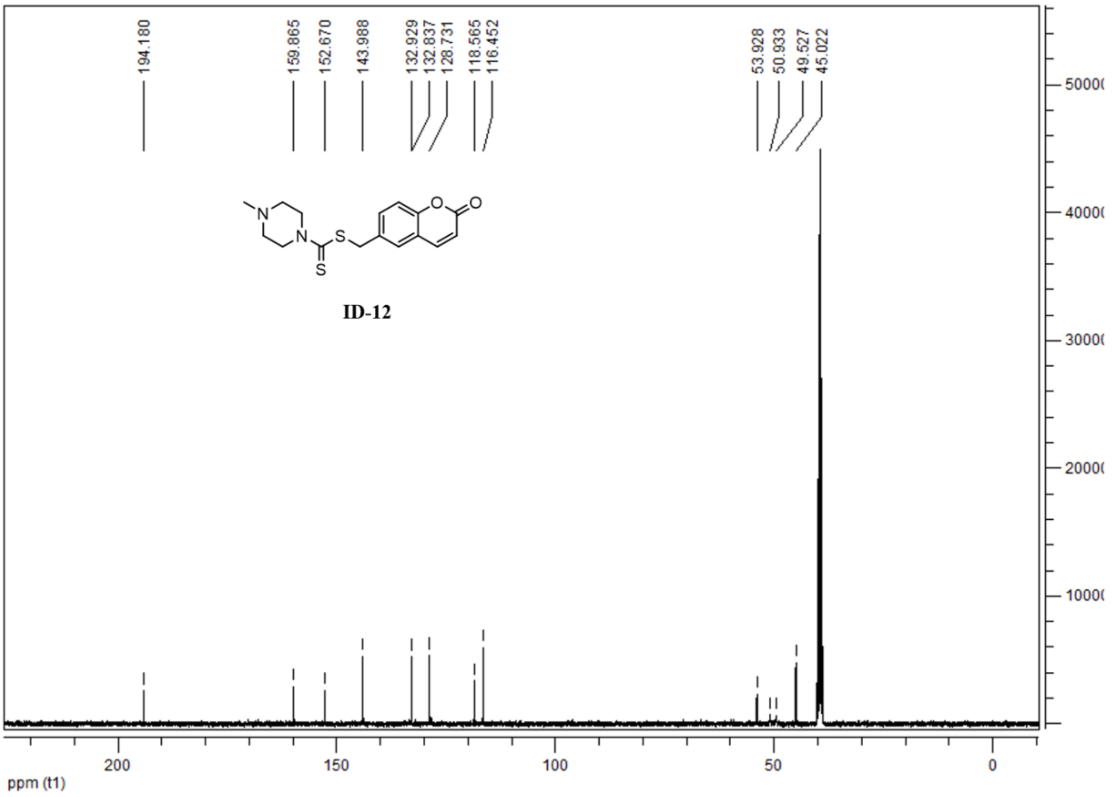

^{1}H NMR, DMSO-d_6（Bruker AVANCE III 400）（**ID-13**）

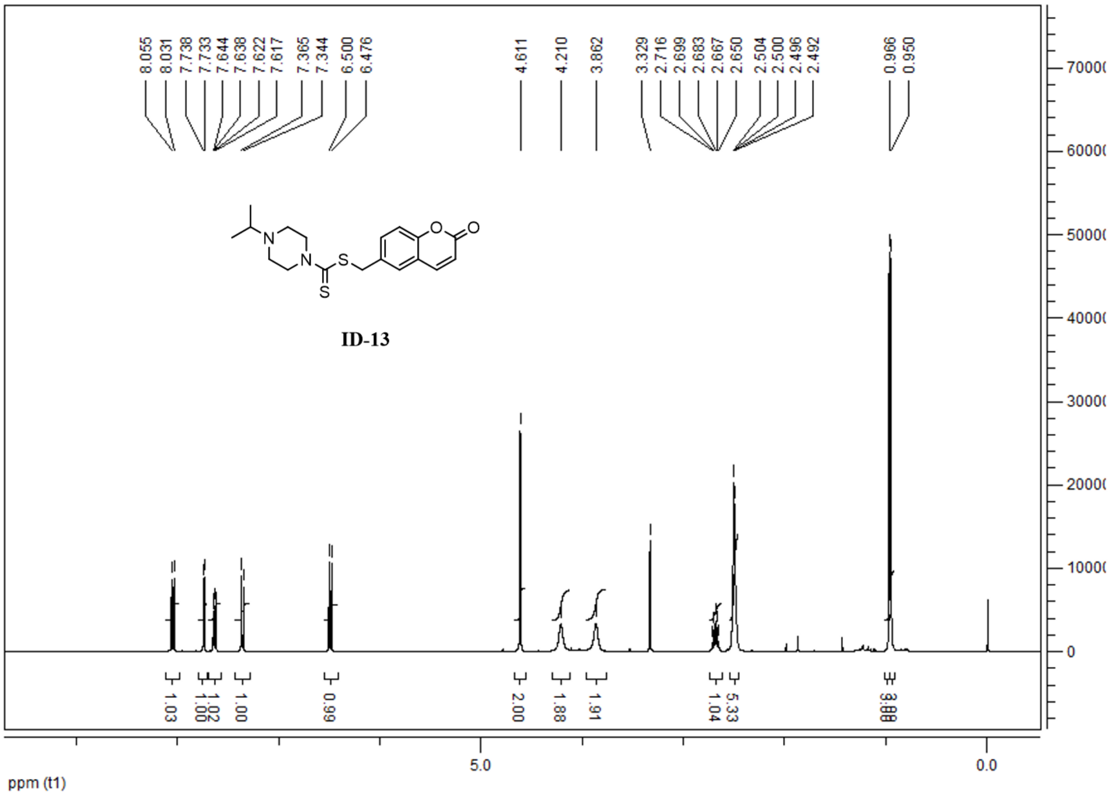

^{13}C NMR, DMSO-d_6（Bruker AVANCE III 400）（**ID-13**）

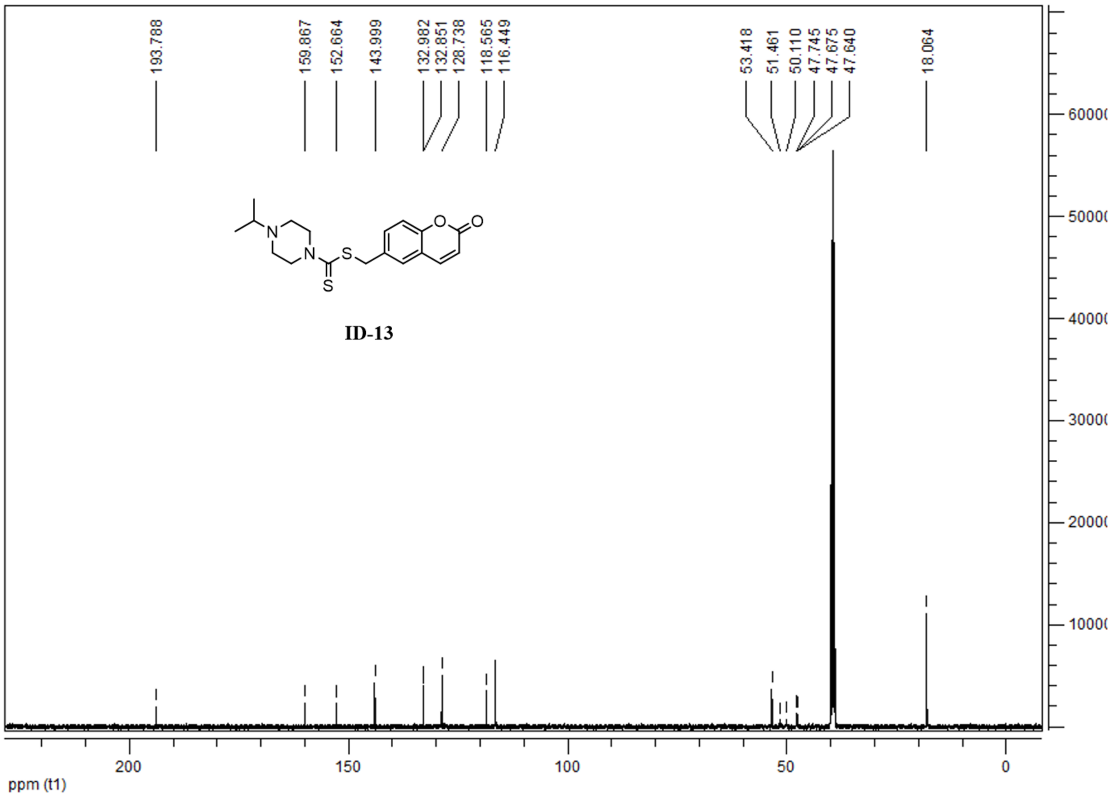

^{1}H NMR, DMSO-d_6（Bruker AVANCE III 400）（**ID-14**）

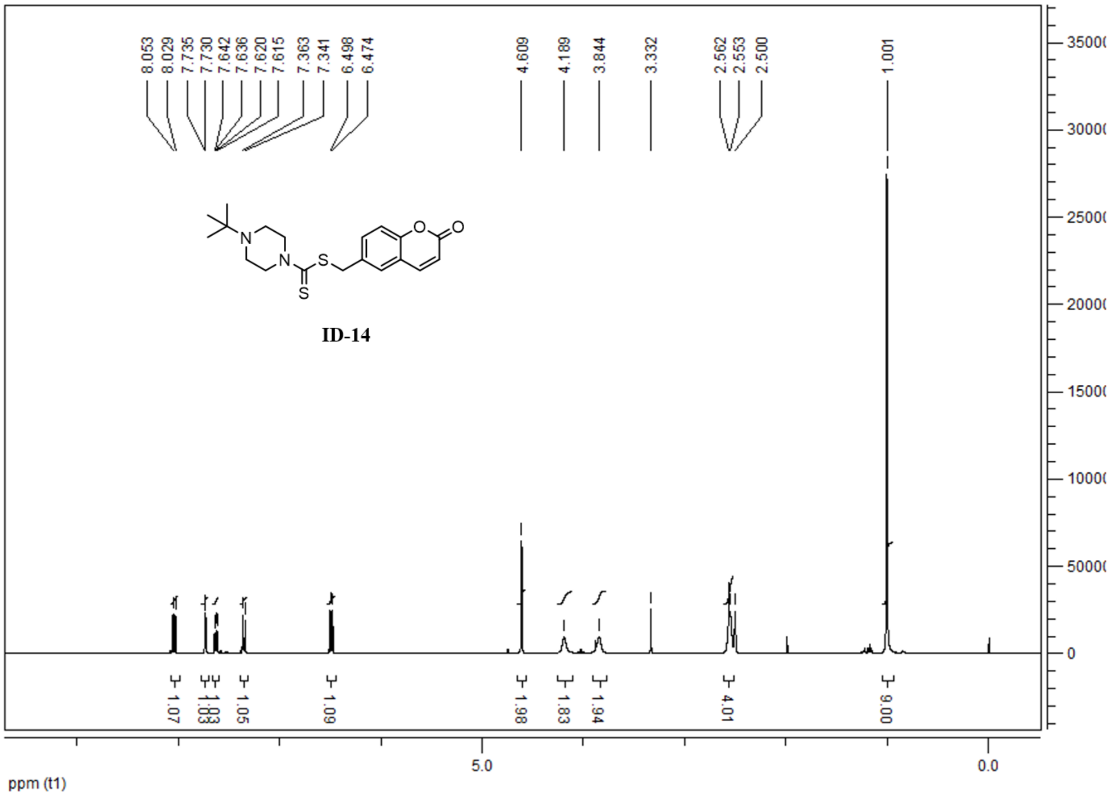

^{13}C NMR, DMSO-d_6（Bruker AVANCE III 400）（**ID-14**）

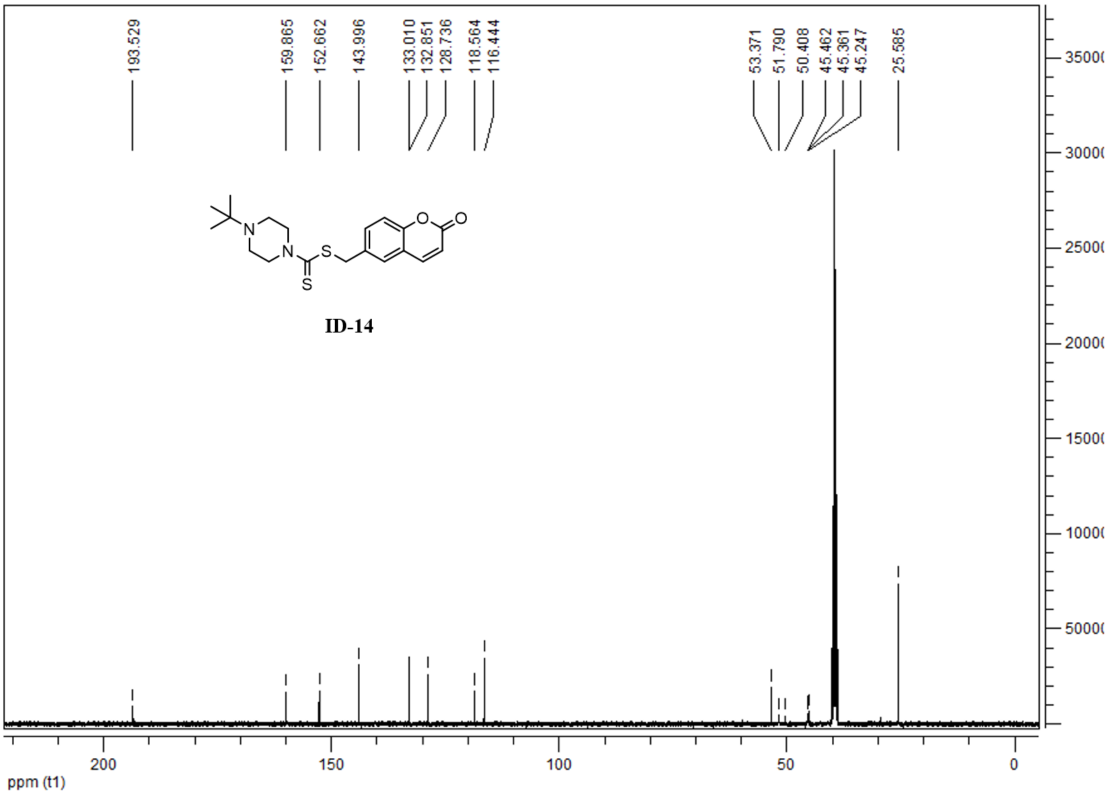

^{1}H NMR, DMSO-d_6（Bruker AVANCE III 400）（**ID-15**）

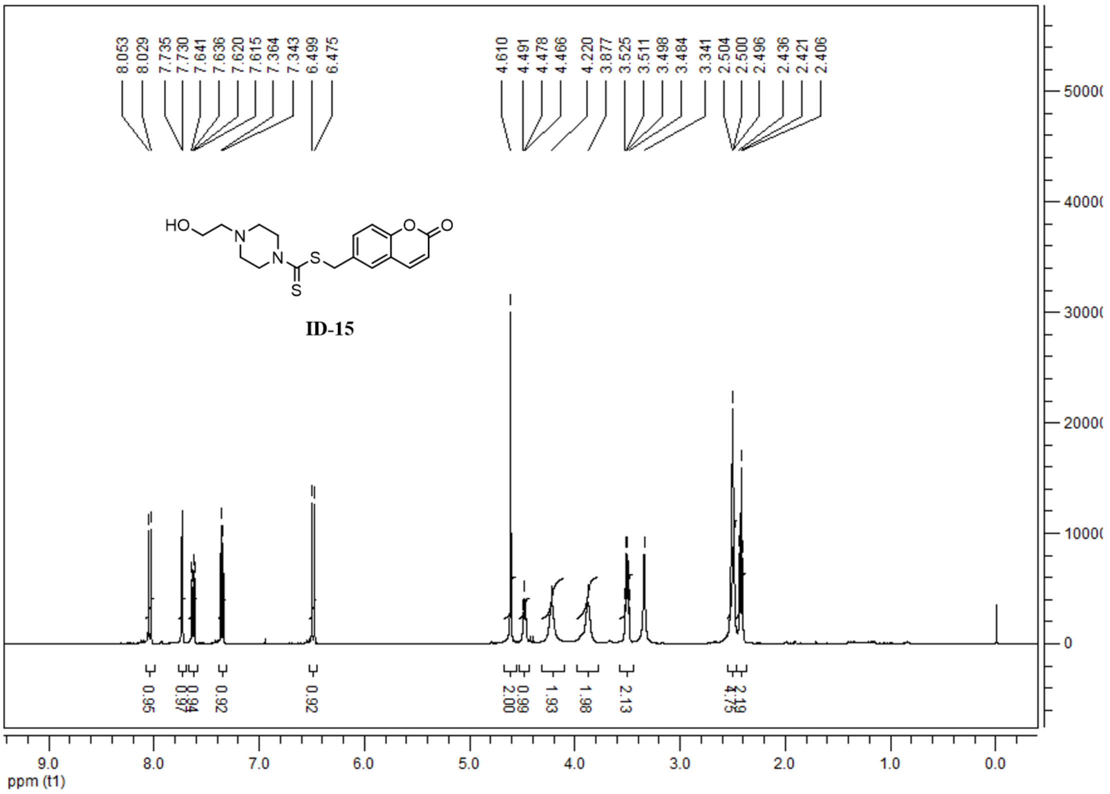

^{13}C NMR, DMSO-d_6（Bruker AVANCE III 400）（**ID-15**）

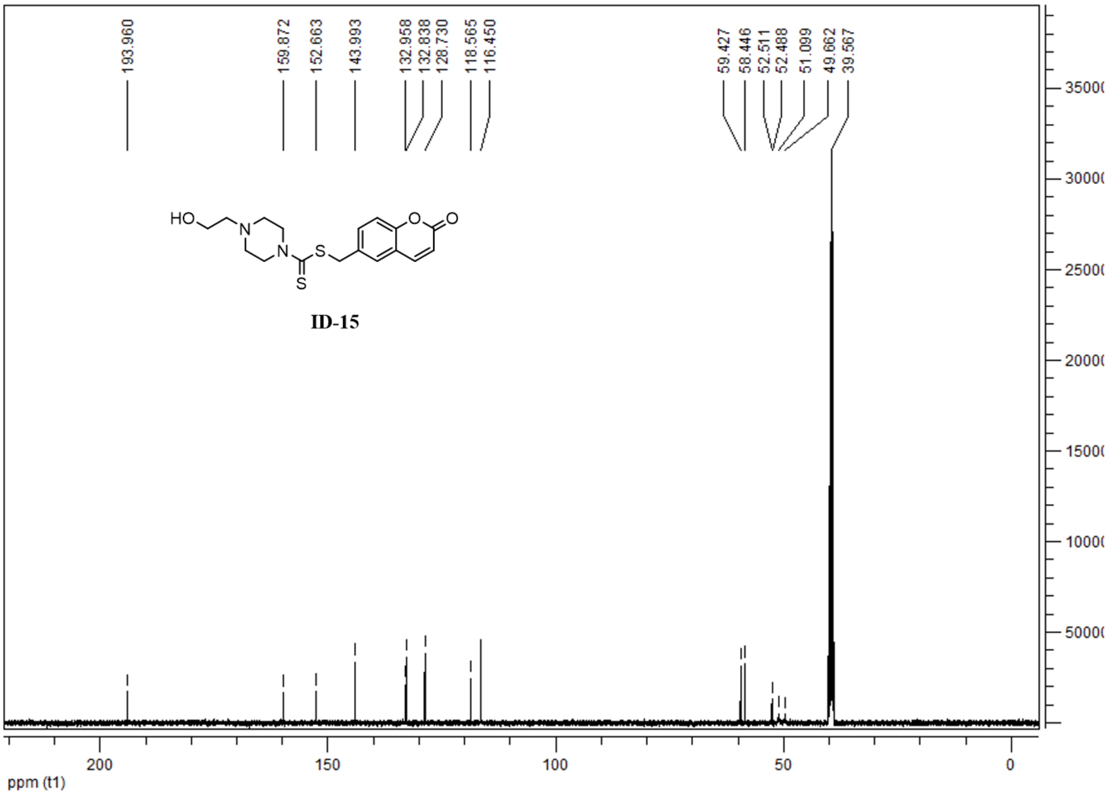

^{1}H NMR, DMSO-d_6（Bruker AVANCE III 400）（**ID-16**）

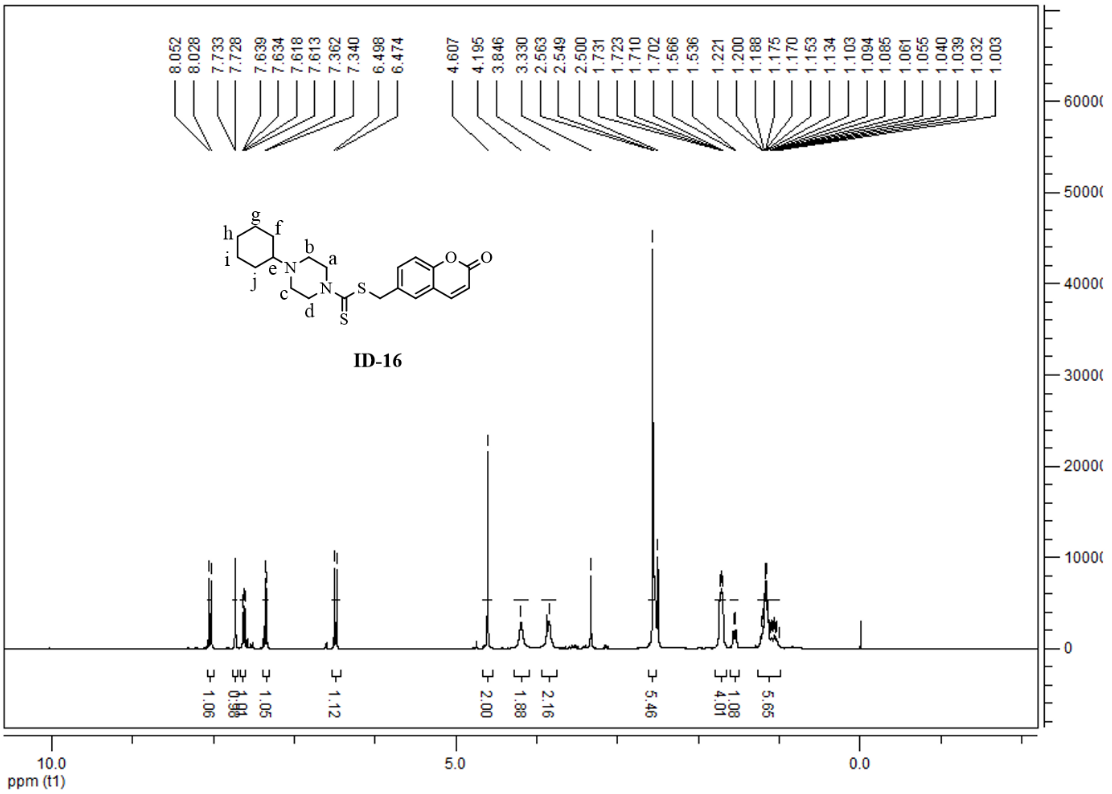

[13] C NMR, DMSO-d_6（Bruker AVANCE III 400）（**ID-16**）

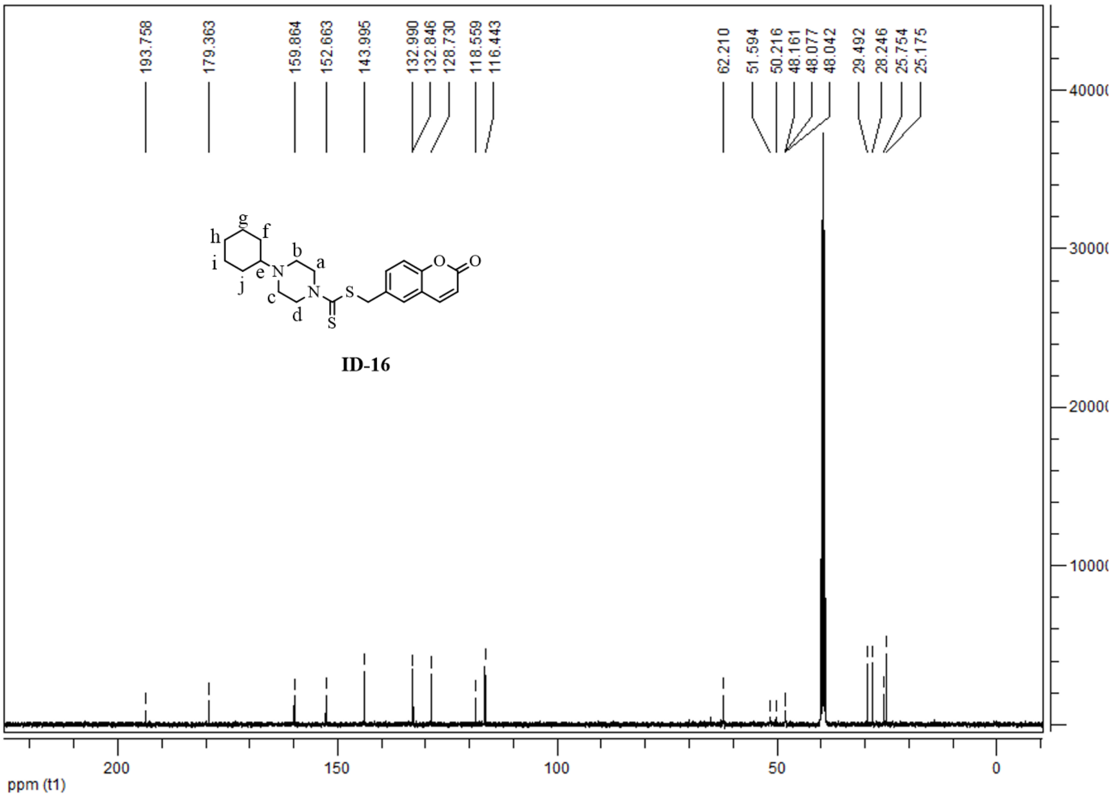

[1] H NMR, DMSO-d_6（Bruker AVANCE III 400）（**ID-17**）

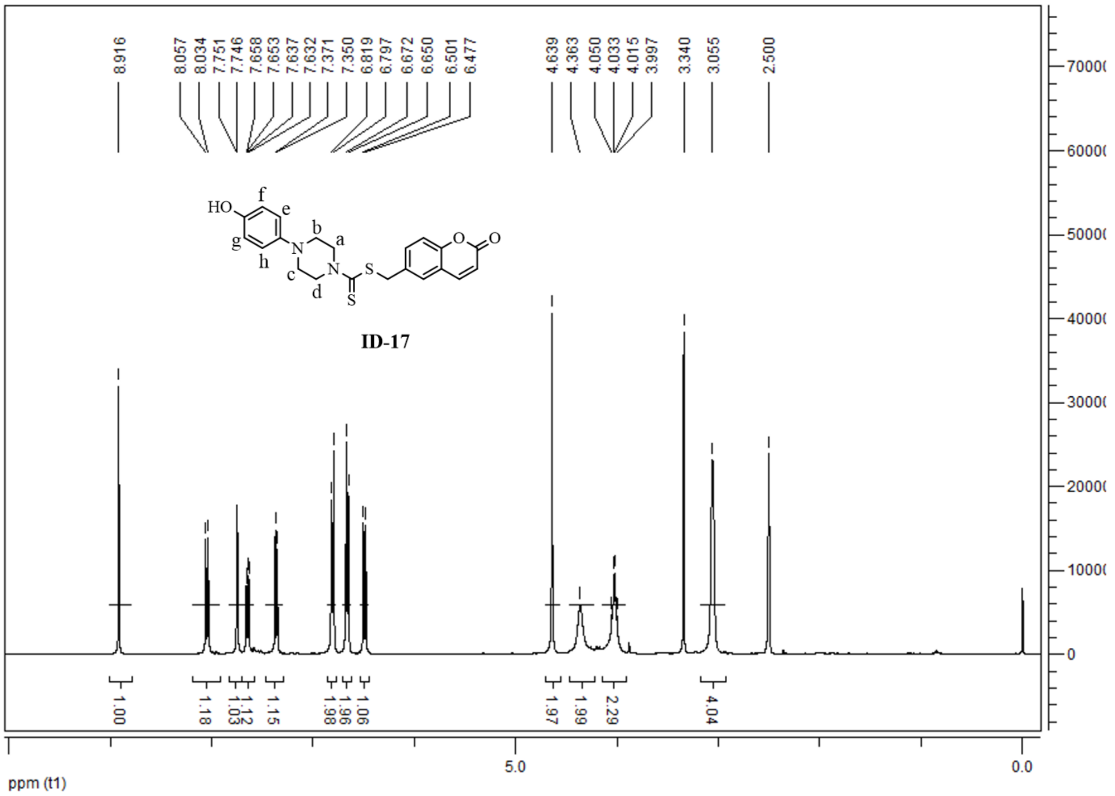

^{13}C NMR, DMSO-d_6（Bruker AVANCE III 400）（**ID-17**）

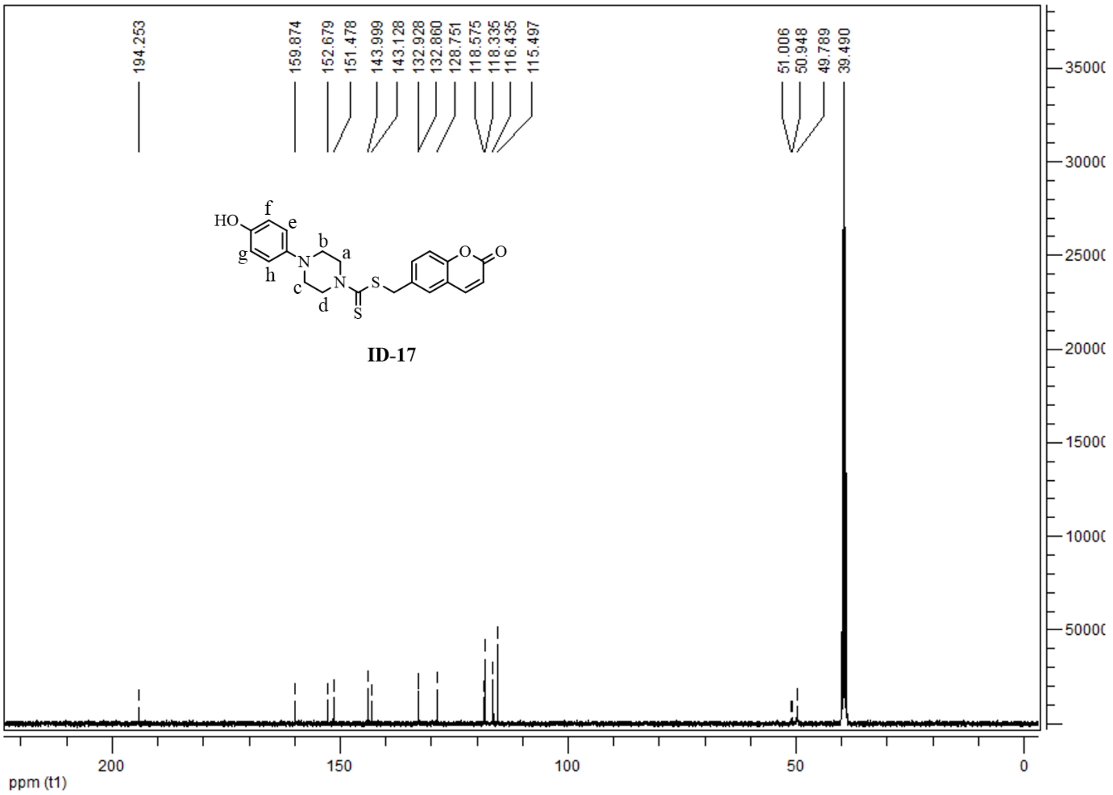

^{1}H NMR, DMSO-d_6（Bruker AVANCE III 400）（**ID-18**）

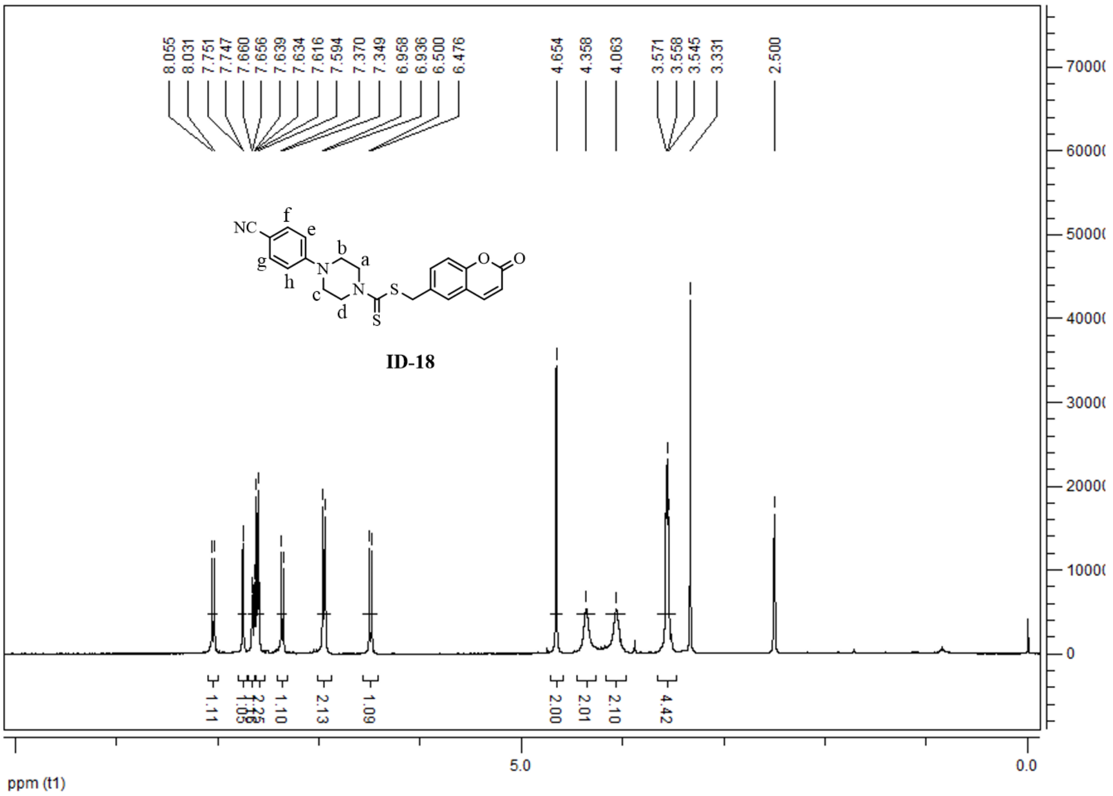

^{13}C NMR, DMSO-d_6（Bruker AVANCE III 400）（**ID-18**）

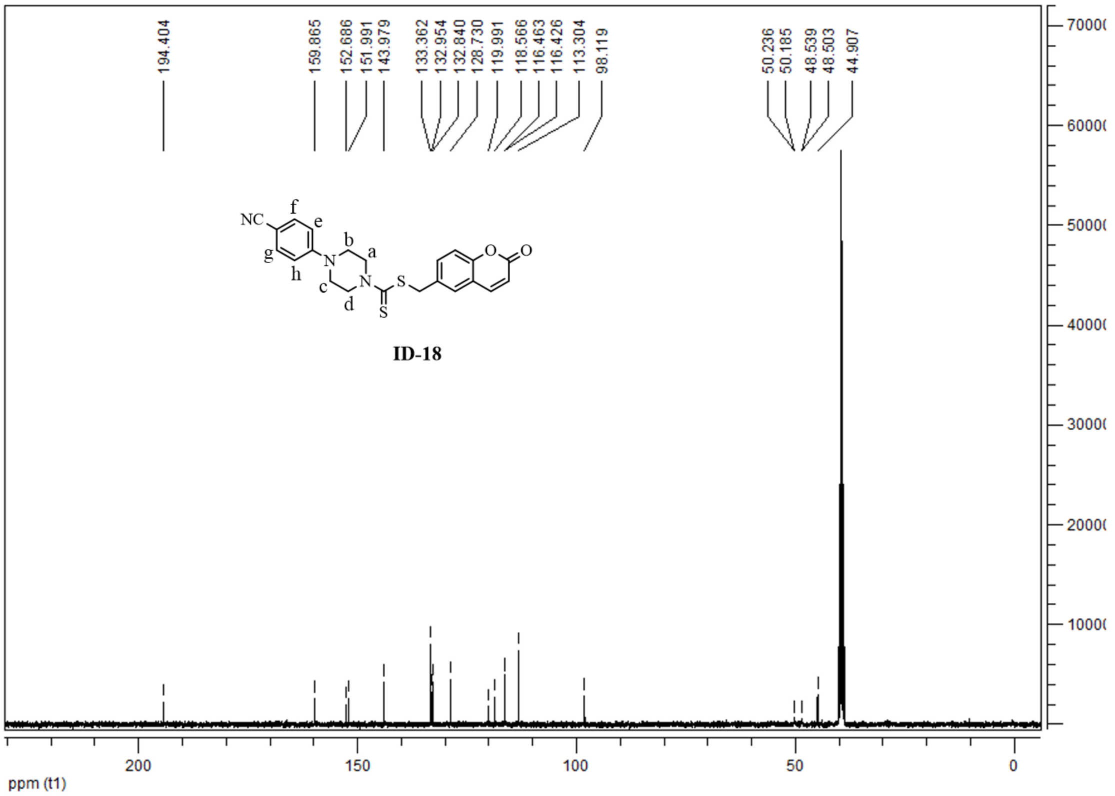

^{1}H NMR, $CDCl_3$（Bruker AVANCE III 400）（**ID-19**）

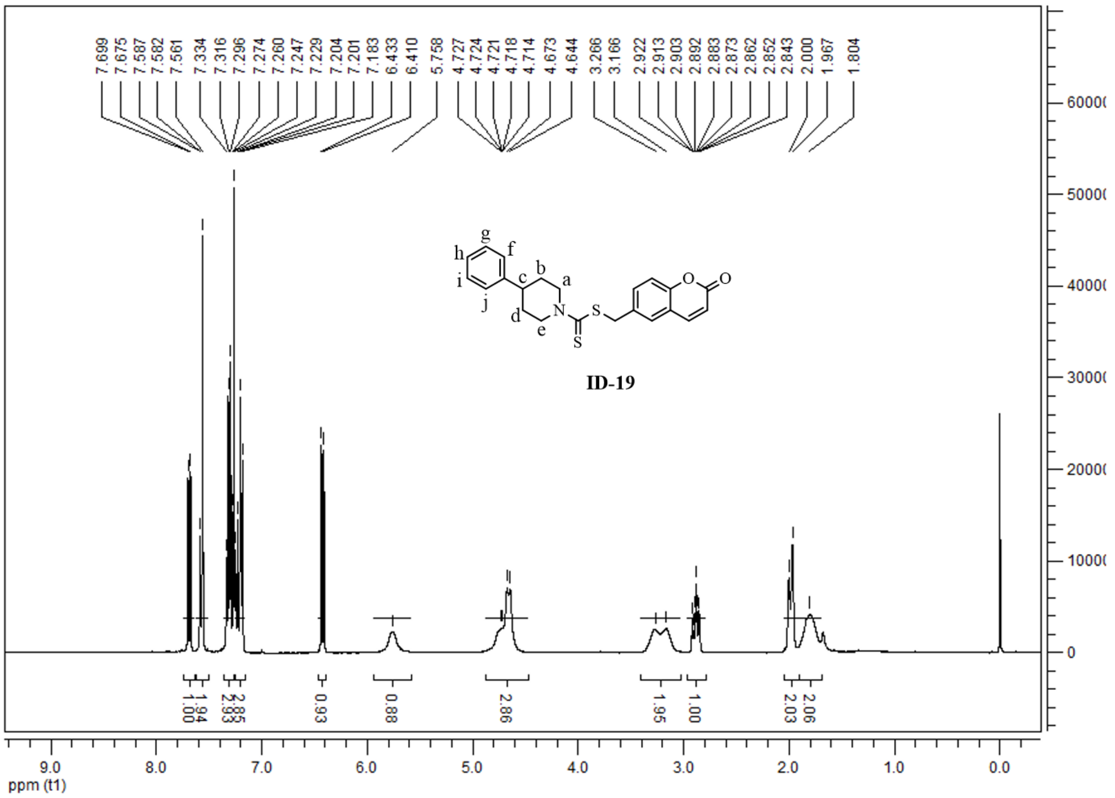

^{13}C NMR, $CDCl_3$（Bruker AVANCE III 400）（**ID-19**）

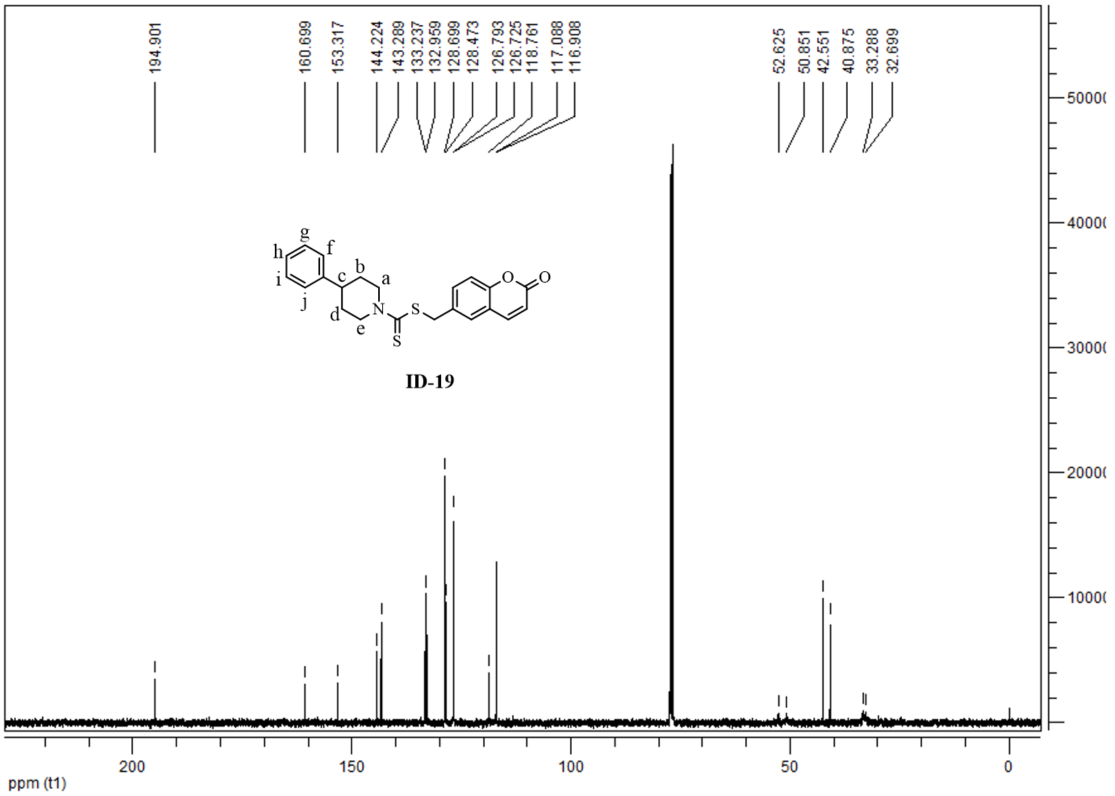

^{1}H NMR, DMSO-d_6（Bruker AVANCE III 400）（**ID-20**）

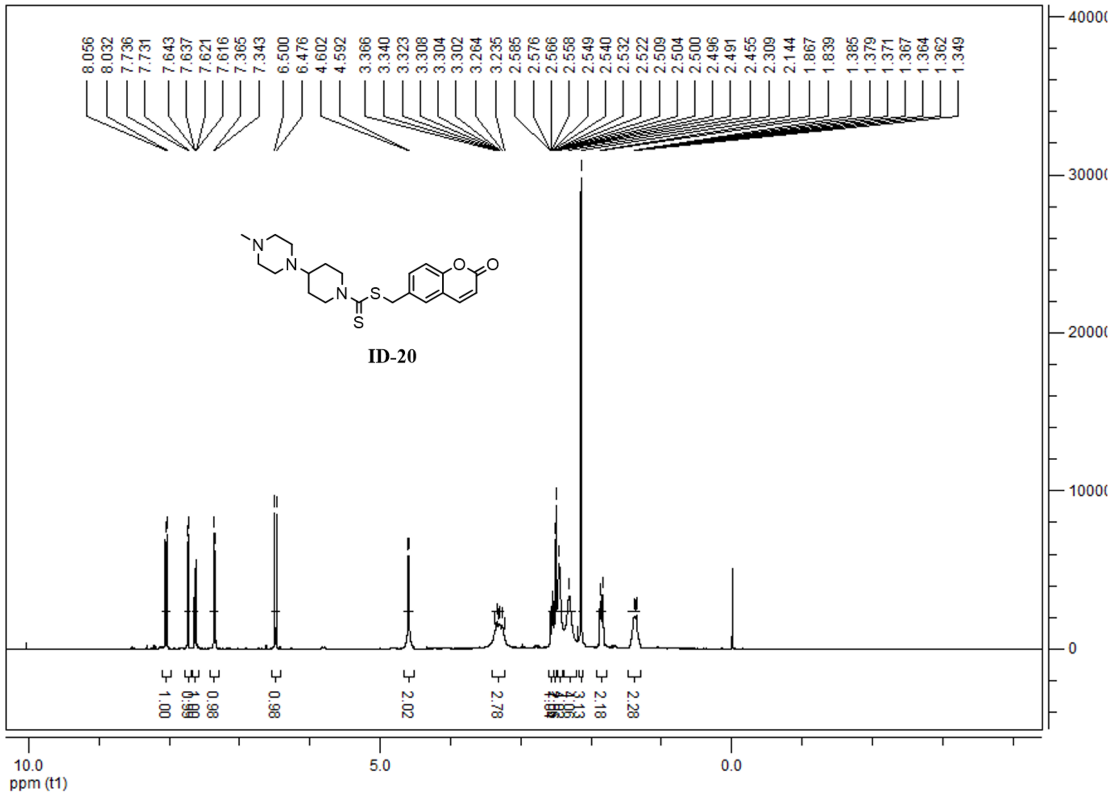

^{13}C NMR, DMSO-d_6（Bruker AVANCE III 400）（**ID-20**）

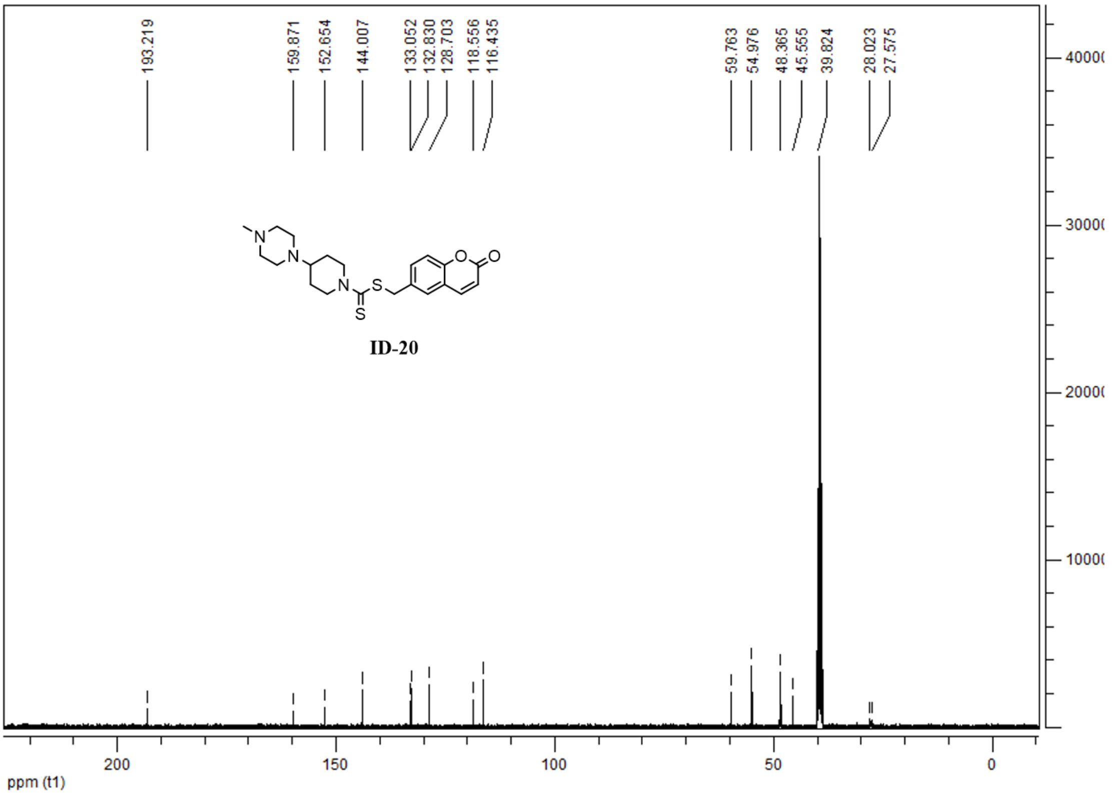

(ID-1): HRMS (ESI, *m/z*) calcd. for $C_{13}H_{13}NO_2S_2$ $(M+Na)^+$ 302.0280, found 302.0282.

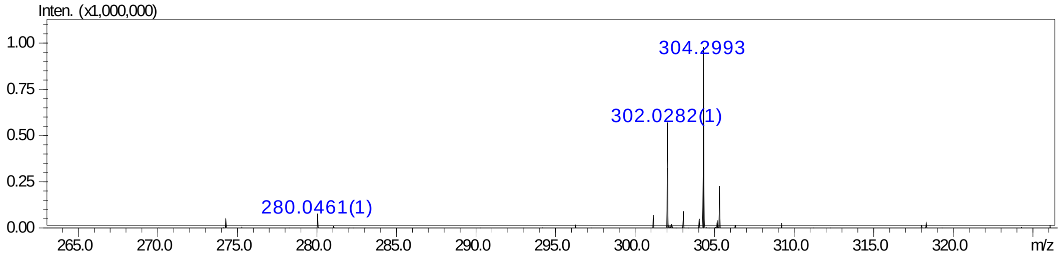

(ID-2): HRMS (ESI, *m/z*) calcd. for $C_{15}H_{17}NO_2S_2$ $(M+Na)^+$ 330.0593, found 330.0599.

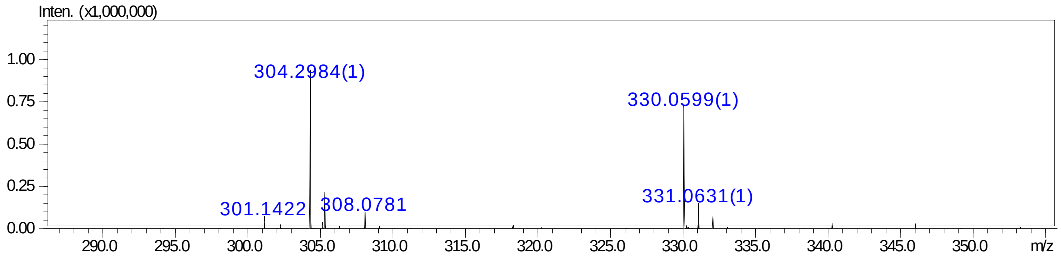

(ID-3): HRMS (ESI, *m/z*) calcd. for $C_{17}H_{21}NO_2S_2$ $(M+Na)^+$ 358.0906, found 358.0918.

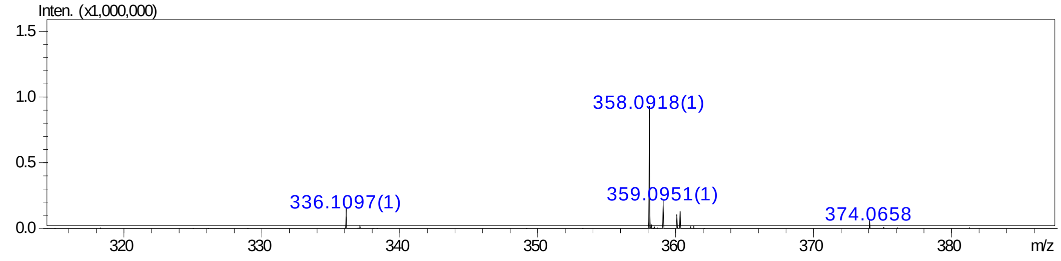

(ID-4): HRMS (ESI, *m/z*) calcd. for $C_{19}H_{25}NO_2S_2$ $(M+Na)^+$ 386.1219, found 386.1222.

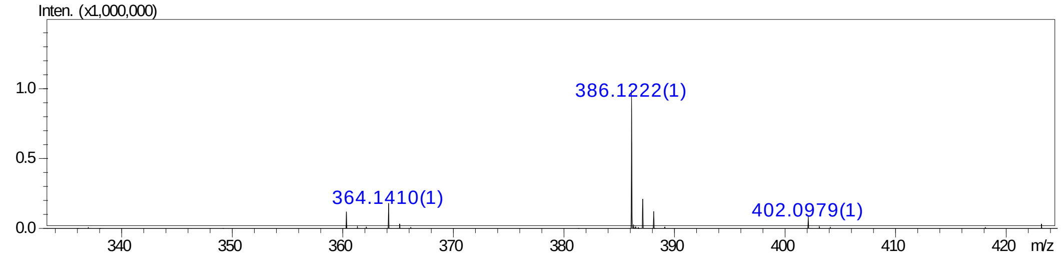

(ID-5): HRMS (ESI, *m/z*) calcd. for $C_{14}H_{13}NO_2S_2$ $(M+Na)^+$ 314.0280, found 314.0281.

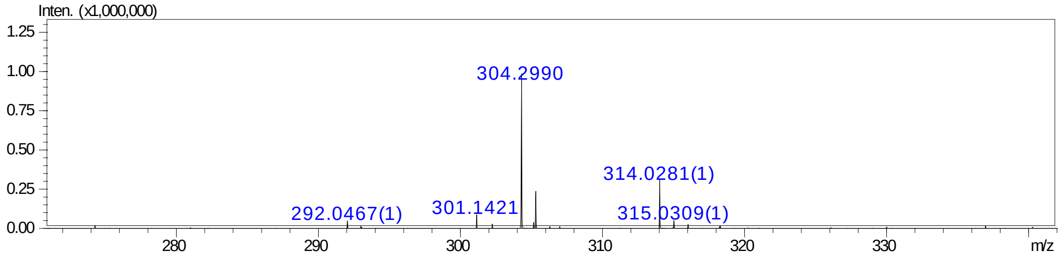

(ID-6): HRMS (ESI, *m/z*) calcd. for $C_{14}H_{13}NO_3S_2$ $(M+Na)^+$ 330.0229, found 330.0232.

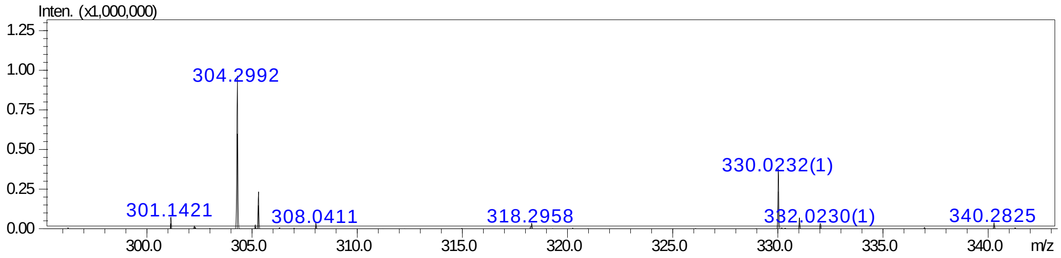

(ID-7): HRMS (ESI, *m/z*) calcd. for $C_{15}H_{15}NO_2S_2$ $(M+Na)^+$ 328.0436, found 328.0444.

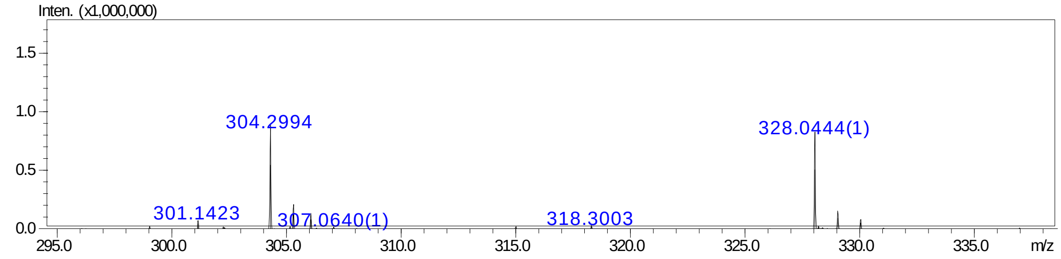

(ID-8): HRMS (ESI, *m/z*) calcd. for $C_{16}H_{17}NO_2S_2$ $(M+Na)^+$ 342.0593, found 342.0585.

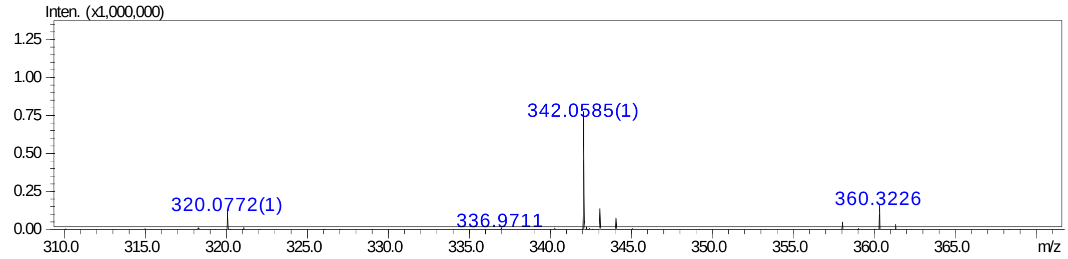

(ID-9): HRMS (ESI, *m/z*) calcd. for $C_{17}H_{19}NO_2S_2$ $(M+Na)^+$ 356.0749, found 356.0751.

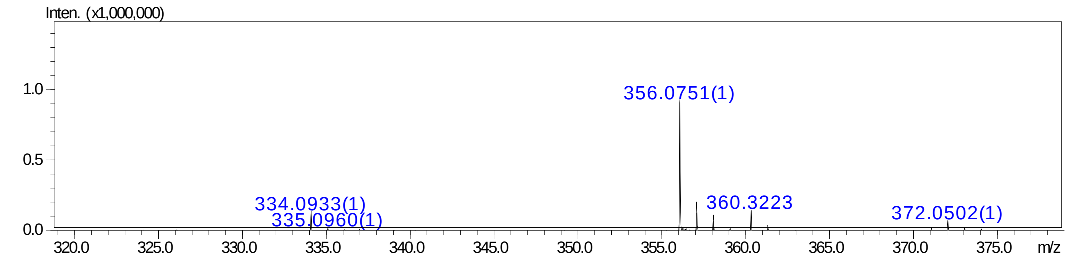

(ID-10): HRMS (ESI, *m/z*) calcd. for $C_{15}H_{15}NO_3S_2$ $(M+Na)^+$ 344.0386, found 344.0391.

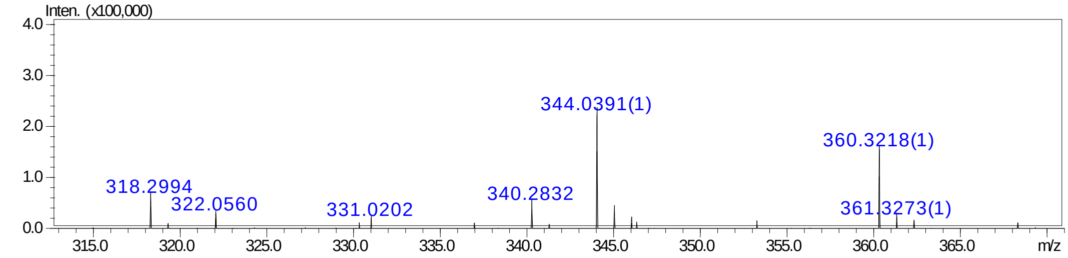

(ID-11): HRMS (ESI, *m/z*) calcd. for $C_{14}H_{10}N_2O_2S_2$ $(M+Na)^+$ 325.0076, found 325.0070.

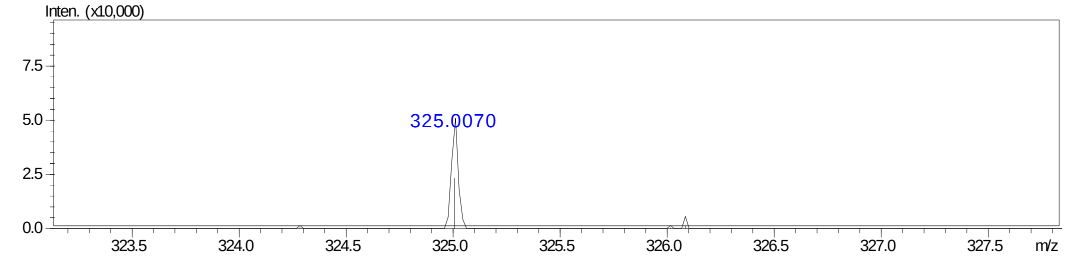

(ID-12): HRMS (ESI, *m/z*) calcd. for $C_{16}H_{18}N_2O_2S_2$ $(M+Na)^+$ 357.0702, found 357.0709.

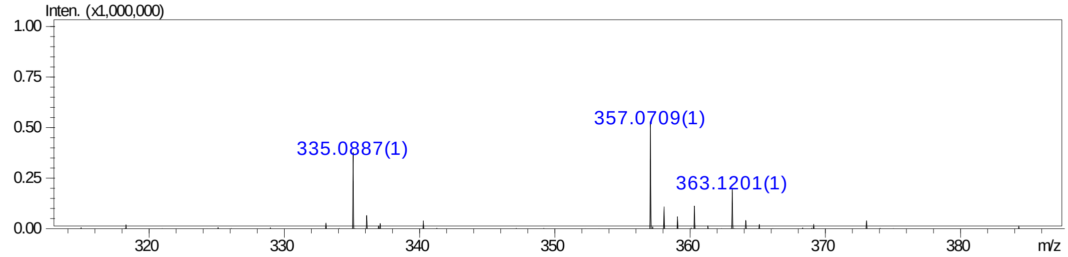

(ID-13): HRMS (ESI, *m/z*) calcd. for $C_{18}H_{22}N_2O_2S_2$ $(M+H)^+$ 363.1195, found 363.1201.

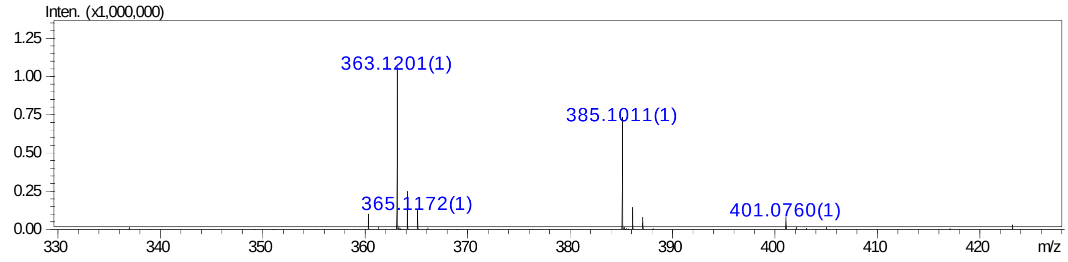

(ID-14): HRMS (ESI, *m/z*) calcd. for $C_{19}H_{24}N_2O_2S_2$ $(M+H)^+$ 377.1352, found 377.1349.

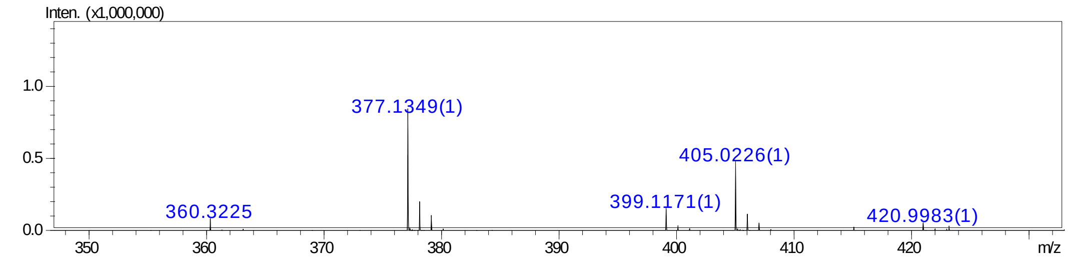

(ID-15): HRMS (ESI, *m/z*) calcd. for $C_{17}H_{20}N_2O_3S_2$ $(M+Na)^+$ 387.0808, found 387.0808.

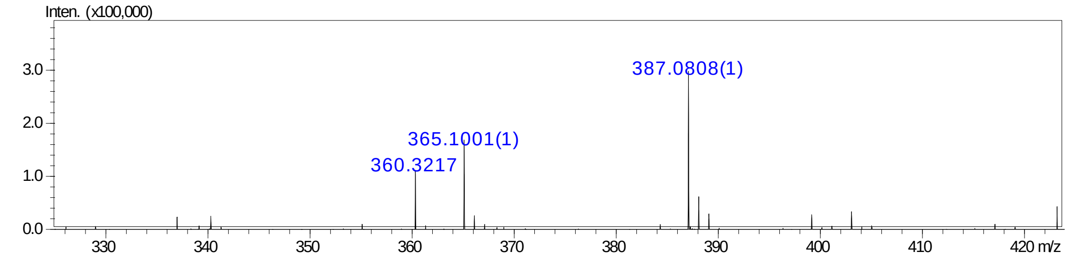

(ID-16): HRMS (ESI, *m/z*) calcd. for $C_{21}H_{26}N_2O_2S_2$ $(M+H)^+$ 403.1508, found 403.1509.

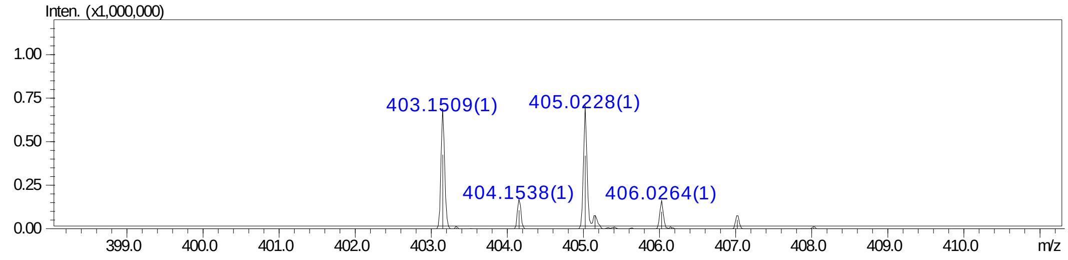

(ID-17): HRMS (ESI, *m/z*) calcd. for $C_{21}H_{20}N_2O_3S_2$ $(M-H)^-$ 411.0843, found 411.0860.

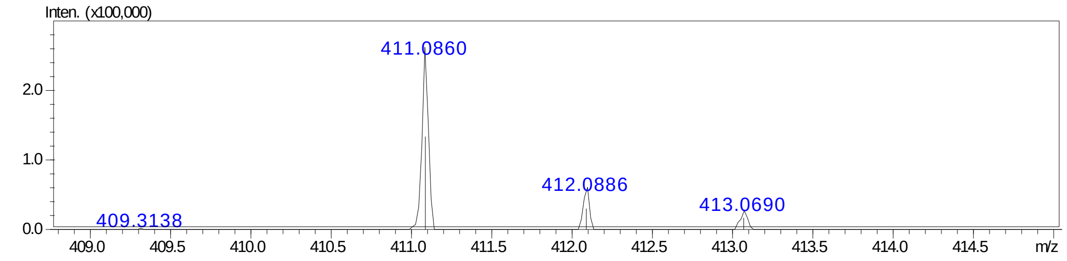

(ID-18): HRMS (ESI, *m/z*) calcd. for $C_{22}H_{19}N_3O_2S_2$ $(M+Na)^+$ 444.0811, found 444.0827.

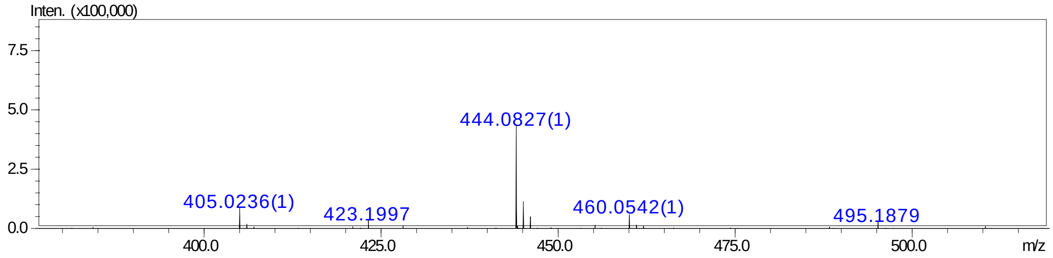

(ID-19): HRMS (ESI, *m/z*) calcd. for $C_{22}H_{21}NO_2S_2$ $(M+Na)^+$ 418.0906, found 418.0910.

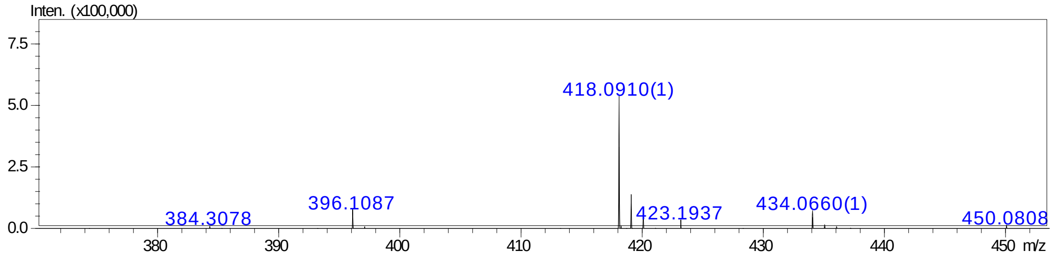

(ID-20): HRMS (ESI, *m/z*) calcd. for $C_{21}H_{27}N_3O_2S_2$ $(M+H)^+$ 418.1617, found 418.1630.

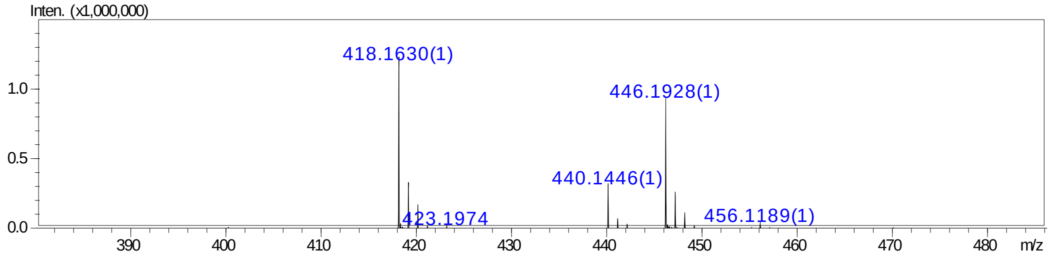

Supplement: Supplemental Material [file IENZ_A_1875458_SM2562.pdf]
